# An Attempt to Understand Kidney's Protein Handling Function by Comparing Plasma and Urine Proteomes

**DOI:** 10.1371/journal.pone.0005146

**Published:** 2009-04-20

**Authors:** Lulu Jia, Ling Zhang, Chen Shao, Eli Song, Wei Sun, Mingxi Li, Youhe Gao

**Affiliations:** 1 Department of Physiology and Pathophysiology, National Key Laboratory of Medical Molecular Biology, Institute of Basic Medical Sciences, Chinese Academy of Medical Sciences, Beijing, China; 2 Department of Nephrology, Peking Union Medical College Hospital, Chinese Academy of Medical Sciences, Beijing, China; Deutsches Krebsforschungszentrum, Germany

## Abstract

**Background:**

With the help of proteomics technology, the human plasma and urine proteomes, which closely represent the protein compositions of the input and output of the kidney, respectively, have been profiled in much greater detail by different research teams. Many datasets have been accumulated to form “reference profiles” of the plasma and urine proteomes. Comparing these two proteomes may help us understand the protein handling aspect of kidney function in a way, however, which has been unavailable until the recent advances in proteomics technology.

**Methodology/Principal Findings:**

After removing secreted proteins downstream of the kidney, 2611 proteins in plasma and 1522 in urine were identified with high confidence and compared based on available proteomic data to generate three subproteomes, the plasma-only subproteome, the plasma-and-urine subproteome, and the urine-only subproteome, and they correspond to three groups of proteins that are handled in three different ways by the kidney. The available experimental molecular weights of the proteins in the three subproteomes were collected and analyzed. Since the functions of the overrepresented proteins in the plasma-and-urine subproteome are probably the major functions that can be routinely regulated by excretion from the kidney in physiological conditions, Gene Ontology term enrichment in the plasma-and-urine subproteome versus the whole plasma proteome was analyzed. Protease activity, calcium and growth factor binding proteins, and coagulation and immune response-related proteins were found to be enriched.

**Conclusion/Significance:**

The comparison method described in this paper provides an illustration of a new approach for studying organ functions with a proteomics methodology. Because of its distinctive input (plasma) and output (urine), it is reasonable to predict that the kidney will be the first organ whose functions are further elucidated by proteomic methods in the near future. It can also be anticipated that there will be more applications for proteomics in organ function research.

## Introduction

A large volume of plasma (350–400 mL/100 g of tissue per min) is filtered by the kidney to generate about 150–180 L/per day ultrafiltrate, and then most components in the ultrafiltrate are selectively reabsorbed until less than 1% of the ultrafiltrating volume is excreted as urine [1]. The physiological processing of these substances by the kidney is composed of filtration, reabsorption, and secretion. There have been extensive studies pertaining to the processing of small molecules by the kidney, such as glucose, amino acid, sodium, chloride, and water. However, knowledge about the protein handling function of the kidney was very limited in the number of proteins studied, which include lysozyme [2], λ-L chain [3], IgG [3], and albumin [4]. A systematic study of how the kidney handles plasma proteins is not available yet. With the help of proteomics technology, it can be studied now with the black box method by comparing the input and output proteomes, which are well represented by the plasma and urine proteomes.

Before the proteomics era, proteins in plasma and urine were identified by enzyme activity experiments, antibody detection, and microsequencing technology, etc[5], [6]. All of the above methods were time consuming and inefficient. As protein identification was greatly restricted by technological constraints at that time, it was very hard to study kidney function by comparing such a limited number of protein data. Due to advances in mass spectrometry technology, proteomics methods prominently improved the ability to identify constituent proteins in complex mixtures. The plasma and urine proteomes have been profiled in much greater detail by different research teams [7]–[14]. Many datasets have been accumulated to form “reference profiles” of the plasma and urine proteomes. It is possible now to at least attempt to systematically study the protein handling part of kidney function by comparing the plasma and urine proteomes.

**Figure 1:**
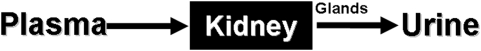
Kidney function analysis by black box method.

In this work, the kidney was regarded as a black box with distinct input and output proteomes. The plasma proteome could be regarded as the input proteome. However, the urine proteome could not be simply regarded as the kidney output proteome due to its complicated protein sources. Urine proteins are derived largely from kidney filtration and secretion, but they are also derived from other sources downstream of the kidney as well, including secretion or shedding from glands and the urine tract [15], [16]. Proteins from these latter sources might complicate the kidney output. For black box analysis, all input should go into the black box, and all output should come directly out of the black box. Anything that can potentially bypass the box should be removed from the system data. Therefore, for analysis of the protein handling portion of kidney function, available proteins secreted into the urine downstream of kidney were subtracted from the plasma and urine proteomes to form the effective input and output proteomes, respectively (Figure 1).

By comparing these modified kidney input and output proteomes, this paper first aims to find which proteins are blocked or permitted to pass through and which proteins are secreted or shed from the kidney. Because these different protein handling pathways in the kidney are closely related to the form and size of individual proteins, the experimental molecular weights (MWs) of proteins are therefore important for understanding the mechanisms of protein handling by the kidney. Then the experimental MWs of the proteins in plasma and urine are compared. Although much work has been done to identify the plasma and urine proteins using strategies including one/two-dimensional gel electrophoresis separation and mass spectrometry, investigators usually reported protein identifications with only theoretical MWs without comparing those values with the corresponding experimental values. There are only a limited number of proteins with experimental MWs published so far in plasma and urine [10], [11], [17]. In the future, it is very important to collect as many experimental data as possible to study kidney functions accurately and comprehensively, such as experimental molecular weight/isoelectric point, protein quantities, and posttranslational modifications.

Data on the plasma and urine proteins identified and analyzed in this study were obtained from different experiments and from different human samples. Two assumptions were implied therein when two proteomes were compared. 1) The plasma and urine samples procured from different individuals at different physiology statuses were comparable. To study functions of a particular kidney, the plasma and urine samples should be acquired from that individual at that particular time point. If we need to draw a conclusion from this individual to the general population, we should assume that the kidney functions of healthy people are similar. If we assume the kidney functions of healthy people are similar sooner or later, we think it is reasonable to compare samples from different healthy individuals from the beginning. 2) The sensitivity of various plasma and urine protein identification methods was comparable even though the plasma proteome is far more complex than the urine proteome. There are certain risks in comparing the two proteomes. Potential problems of the hypothesis and solutions will be discussed.

## Materials and Methods

### Datasets

The Human Proteome Organization Plasma Proteome Project (HUPO PPP) data were obtained directly from the project website at the University of Michigan at Ann Arbor (http://www.bioinformatics.med.umich.edu/hupo/ppp). The 3020 proteins of the core dataset were downloaded. Another plasma proteome dataset of 889 proteins with high confidence were obtained from David J States et al[18]. The plasma protein identifications with experimental molecular masses were collected by Jin Young Kim et al [17].

A total of 1543 urinary proteins identified by Matthias Mann with high confidence were downloaded from http//proteome.biochem.mpg.de/ [14]. The urinary protein identifications with experimental molecular masses were obtained from Rember Pieper et al [10] and Jisum Oh et al [11].

The 114 human prostatic secretion proteins were acquired from Biaoyang Lin et al [19].

### Protein sequence database

All protein identifiers presented here were derived from version 3.24 of the International Protein Index (IPI) database [20] downloaded from ftp://ftp.ebi.ac.uk/pub/database/IPI/old/HUMAN.

### IPI accession number conversion algorithm

Using the IPI history file from ftp://ftp.ebi.ac.uk/pub/databases/IPI/current/ipi.HUMAN.history.gz, all reported IPI identifiers from previous versions were converted to IPI version 3.24. The algorithm employed kept track of multiple chained propagations and only halted at deletions [21].

Using the human.xrefs files for IPI 3.24 from ftp://ftp.ebi.ac.uk/pub/databases/GO/goa/HUMAN/, the accession numbers of urinary proteins other than IPI were converted to IPI version 3.24 accession numbers.

### Calculation of theoretical molecular mass

The protein sequences were extracted from IPI 3.24 according to their accession numbers and then submitted to the website (http://www.expasy.org/tools/pi_tool.html) to utilize the Compute pI/MW tool to calculate their theoretical molecular masses.

### Enrichment analysis of Gene Ontology categories

BiNGO, a plugin of Cytoscape, was used for Gene Ontology (GO) category enrichment analysis [22]. A custom GO annotation file for the input-plasma proteome as the reference dataset was created with instructions on the BiNGO webpage by extracting the GO annotations available for input-plasma identifiers from EBI Gene Ontology Annotation (GOA) Human 62.0 release (http://www.ebi.ac.uk/GOA/). The plasma-and-urine subproteome dataset was tested against the input-plasma proteome dataset (the reference dataset) for the enrichment analysis. The analysis was done using the ‘hyper geometric test’, and all GO terms that were significant with P<0.001 (after correcting for multiple term testing by Benjamini and Hochberg false discovery rate corrections) were selected as overrepresented.

## Results

### Kidney input proteome and output proteome

The plasma proteome project (PPP) initiated by HUPO in 2002 is an international collaboration to catalog the protein composition of human blood plasma and serum by analyzing standardized aliquots of reference serum and plasma specimens [7]. It resulted in 9504 proteins identified by at least one peptide and in 3020 proteins identified by at least two distinct matching peptides [23]. Recently, there was another research team using a thorough statistical analysis based on the chances of random matching to a protein to reduce this list to a set of 889 proteins that can be considered identified at a confidence level of at least 95%[18]. Considering the confidence and comprehensiveness of the data, the ‘core dataset’ of 3020 proteins and the high confidence 889 proteins were pooled together to generate 3269 proteins which was selected as the plasma proteome in this study. It was notable that all of the proteins had no experimental MWs.

A total of 292 proteins in plasma were identified by Jin Young Kim et al using both SEQUEST searching[24] and a protein data filtration method based on correlation (MWcorr) between the experimental (1-DE) and the theoretical MW[17], although more than half of the assignments were based on single peptide identification. Because the potential for erroneous identification based on single peptide identification is greatly increased, proteins not present in the plasma proteome selected in this study were removed from the 292 proteins. Finally, 184 proteins were acquired as the plasma proteins with experimental MW information [17].

Matthias Mann and his colleagues provided a high-accuracy proteome map of urine with high-accuracy mass spectrometry. A total of 1543 proteins were identified with a high degree of overlap with a total of about 800 proteins reported before his work [14]. None of the identified proteins had experimental MW information.

The experimental MW information of urinary proteins was collected from other studies. More than 150 proteins from Rember Pieper et al [10] and 100 proteins from Jisun Oh et al [11] were incorporated as the urine proteome with experimental MW information. Finally, the identifiers from Matthias Mann, Rember Pieper, and Jisun Oh were pooled together to represent the urine proteome in this study.

IPI 3.24 was selected as the standard protein database to make protein accession numbers comparable. All of the previous protein accession numbers were converted to IPI version 3.24.

After conversion, the whole plasma proteome was reduced from 3269 to 2666 with 1946 unchanged accession numbers, 745 recovered as propagated entries, 578 deleted, and 25 identifiers lost due to merging. For the 184 proteins of plasma proteome with experimental MWs, 168 proteins were collected with 147 unchanged, 22 recovered as propagated entries, 15 deleted, and 1 identifier lost due to merging.

For urinary proteins without experimental MWs from Matthias Mann, 1503 identifiers were recovered with 1453 unchanged identifiers, 58 propagated, 32 deleted, and 8 lost due to merging. For urinary proteins with experimental MWs, 97 unique proteins were obtained from Jisun Oh, and 131 proteins were obtained from Rember Pieper, after converting their accession numbers from Swiss-Prot to IPI 3.24. Since 19 proteins were shared in both datasets, overall 209 unique proteins were adopted from these two datasets. After removing all of the redundant proteins, protein identifiers from Matthias Mann, Rember Pieper, and Jisun Oh were pooled together to generate 1607 proteins as the urine proteome in this study.

The prostate is a secretory gland downstream of the kidney that might secrete prostatic fluid into urine, which would influence the kidney output proteome. Human prostatic secretion proteins identified by proteomics methods were hence removed from the urine proteome to generate a valid output proteome [19]. These prostatic secretion proteins present in urine were also removed from plasma proteome to generate a valid input proteome, because it is unclear whether those proteins could be filtered through the kidney or blocked by kidney and then secreted into the urine downstream of the kidney. Other proteins that are possibly incorporated in urine downstream of kidney were temporarily ignored due to limited knowledge at this time.

Data from Biaoyang Lin et al were the only prostatic secretion proteome available at this stage [19]. One hundred fourteen prostatic secretion proteins were converted to a total of 111 proteins with IPI 3.24 accession numbers. Eighty-five of them were shared in the 1607 urinary-protein dataset, and 27 were present in the 209 urinary proteins with experimental MWs. All of these shared proteins were removed from the urinary proteins. The rest of the proteins were regarded as the kidney urine-output proteome, which were 1522 proteins for the whole urinary proteome and a subset of 182 proteins with experimental MWs, respectively.

There were 55 proteins shared by the 111-protein prostatic secretion proteome, 1607-protein urine proteome, and 2666-protein plasma proteome datasets. It was difficult to judge if the 55 proteins went through the kidney or not because the prostate gland might secrete them into urine. For the purpose of studying the protein handling aspect of kidney function, all of these proteins were removed from the plasma proteome, with the rest of the proteins regarded as the effective plasma-input proteome. It resulted in 2611 proteins for the whole plasma proteome and 142 proteins for the plasma proteome with experimental MWs.

Based on analysis above, kidney plasma-input proteome and urine-output proteome were generated. The description of preparing the two datasets is summarized as table 1.

**Table 1:** Summary of the preparation of the plasma-input proteome and urine-output proteome for comparing.

	Plasma-input proteome Without(with) experimental MW	Urine-output proteome Without(with) experimental MW	Human prostatic secretion proteins
Before conversion	3269(184)	1543(150[1]+100[2])	114
Converted to IPI V3.24	2666(168)	1503(131[1]+97[2])	111
Sum	2666(168)	1607(209)	
Removing prostatic secretion proteins	2611(142)	1522(182)	

[1] Pieper R, Gatlin CL, McGrath AM, Makusky AJ, Mondal M, et al. (2004) Characterization of the human urinary proteome: a method for high-resolution display of urinary proteins on two-dimensional electrophoresis gels with a yield of nearly 1400 distinct protein spots. Proteomics 4: 1159–1174.

[2] Oh J, Pyo JH, Jo EH, Hwang SI, Kang SC, et al. (2004) Establishment of a near-standard two-dimensional human urine proteomic map. Proteomics 4: 3485–3497.

### Three subproteomes from comparing kidney input with output proteomes

**Figure 2:**
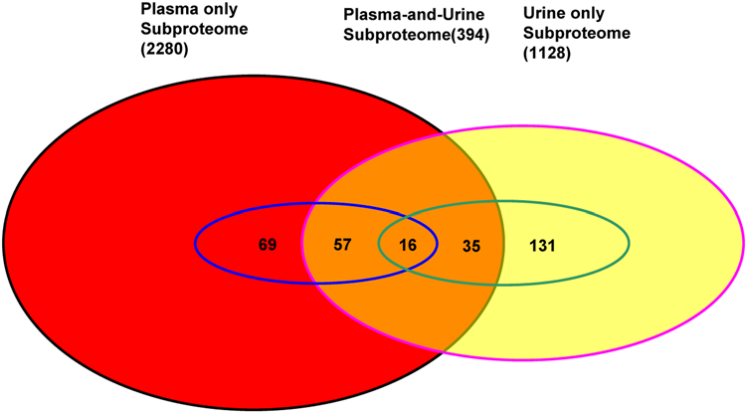
Diagram of proteins found in the three subproteome datasets. There are 2280 proteins in the plasma-only subproteome (red), 394 proteins in the plasma-and-urine subproteome (orange), and 1128 proteins in the urine-only subproteome (yellow). The proteins in blue circles (142 proteins) and green circles (182 proteins) represent effective input-plasma proteins with experimental MWs and effective output-urine proteins with experimental MWs, respectively. Numbers represent the number of shared proteins in the respective overlapping areas.

Three subproteomes were generated after comparing the kidney input and output proteomes, namely, the plasma-only subproteome, the plasma-and-urine subproteome, and the urine-only subproteome. Then, each protein in the urine-only subproteome was further manually searched on the plasma proteome website (http://www.plasmaproteomedatabase.org/query) to determine whether it is present in plasma. In total, 63 proteins were retrieved as present both in plasma and urine. Finally, the plasma-only subproteome consisted of 2280 proteins exclusively in the plasma, the plasma-and-urine subproteome consisted of 394 proteins common in both plasma and urine, and the urine-only subproteome consisted of 1128 proteins exclusively found in urine (Figure 2, Tables S1, S2, and S3).

In the 142 effective plasma-input proteins with experimental MWs, there were 69 proteins present exclusively in plasma and 73 proteins present both in plasma and urine. In the 182 effective urine-output proteins with experimental MWs, there were 131 proteins found only in urine and 51 proteins found both in plasma and urine. In the plasma-and-urine subproteome composed of 394 proteins described above, there were 16 proteins that had experimental MWs both in plasma and urine, 57 proteins only in plasma, and 35 proteins only in urine (Figure 2, Tables S1, S2, and S3).

### Kidney function described in proteomics language

The function of the kidney can be described in itemized proteomics language as whether a particular protein is blocked, permitted to pass, or secreted/shed from the kidney. These three groups of proteins correspond to the three subproteomes generated by comparing kidney input and output with the kidney considered as a black box.

As shown in Figure 2, there were 2280 proteins in the plasma-only subproteome. Currently, there was no evidence that they were present in urine based on available proteomic data. These proteins are supposed to be difficult to pass through the kidney black box. Experimental MWs of 69 proteins were available, ranging from 6 kDa to 137 kDa, with 11 of them below 30 kDa. Thirty-three of these 69 proteins had experimental MWs within 20% variation from the theoretical values, 15 proteins were larger than 120% of the theoretical MWs, and the remaining 21 were smaller than 80% of the theoretical values. This included proteins that could not be filtered at the glomerular capillaries or filtered but reabsorbed completely back into the blood from the tubules. Changes in this subproteome may reflect changes in glomerular and tubule functions.

There were 394 proteins that existed in both plasma and urine. They may pass the kidney black box in various forms. There were only 16 proteins with experimental MW information available from both plasma and urine, which range from 11 kDa to 133 kDa in plasma and 11 kDa to 77 kDa in urine. Comparing their MWs in plasma and in urine, six proteins were within 20% variation, suggesting they may pass through the kidney in an intact form; five had MWs 20% higher in plasma than in urine, and five had MWs 20% lower in plasma than in urine. These differences reflect functions of the kidney. Thirty-five of these 394 proteins had experimental MWs available only in urine, ranging from 15 kDa to 82 kDa, of which 20 were within 20% variation of theoretical values, 3 were larger than 120%, and 11 were smaller than 80% of theoretical MWs, and one with multiple fragments and therefore omitted. Fifty-seven proteins in the plasma-and-urine subproteome had experimental MWs available only in plasma, ranging from 6 kDa to 136 kDa, 25 of which were within 20% variation of theoretical values, 18 were larger than 120%, and 14 were smaller than 80% of theoretical MWs. At this stage, we could not distinguish proteins filtered at the glomerular capillaries from those that were blocked at the glomerular capillaries but also secreted or shed from the kidney into the urine.

It is believed that proteins with a MW of <15 kDa are freely filtered in the glomeruli; proteins up to 45 kDa are quite rapidly filtered and proteins between 45 to 60 kDa only restrictedly. Plasma proteins larger than 60 kDa are not filtered through the kidney[25]. We found some proteins with experimental MW<45 kDa exist in the plasma but have not been identified in the urine proteomic data until now. There were some possible mechanisms. For example they might bind to larger carrier proteins or there might be some unknown mechanisms for them to be retained for an extended period in the plasma. We have also found that some proteins with experimental MW>60 kDa had been identified both in plasma and urine such as IPI00020996 (experimental molecular weight 80 kDa in plasma and 77 kDa in urine) and IPI00291866 (experimental molecular weight 91 kDa in plasma and 75 kDa in urine). These proteins might be secreted but the passing through the glomeruli could not be ruled out. This is worth of further study.

One thousand one hundred twenty-eight proteins were identified only in urine, but not in plasma, by proteomics methods. Proteins secreted or shed from the kidney are thought to be included in this group. Since the plasma proteomic data were filtered with high stringency, there were potentially a lot of false negative proteins. In other words, some of the 1128 proteins might exist in plasma but were missed because of the stringent filtering criteria, so they may have been determined to belong to plasma-and-urine subproteome instead of urine-only one. One hundred thirty-one of these proteins had experimental MWs available, ranging from 10 kDa to 120 kDa, in which 97 were within 20% variation of their theoretical values, 7 were larger than 120%, and 25 were smaller than 80% of the theoretical MWs, and 2 were omitted due to the existence of multiple fragments. Changes in the urine-only subproteome may reflect functional changes of the kidney directly.

**Figure 3:**
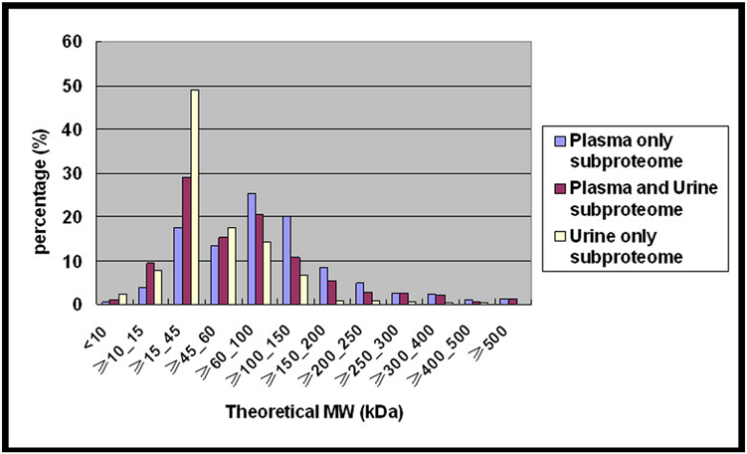
Theoretical molecular weight distributions for the three subproteomes, plasma-only subproteome, plasma-and-urine subproteome, and urine-only subproteome.

In the urine data, there were a total of 182 proteins with experimental MWs available. One hundred twenty-six of them had experimental and theoretical MWs within 20% difference, suggesting that a high percentage of urinary proteins probably existed in intact forms in urine (data not shown). The distribution of theoretical MWs for the three subproteomes is shown in Figure 3. Because the variance between theoretical and experimental MWs was not very big in the urine data with experimental MWs available, the theoretical MW distributions might roughly correlate to the true MW distributions of these three subproteomes. It was notable that many small proteins in the plasma-only subproteome were blocked from passing the kidney. Since post-translational modifications generally increase the actual MW of a protein by no more than 10–40% [17], blockage of those small proteins lower than 30 kDa in the plasma-only subproteome may involve mechanisms other than molecular weight, such as molecular charge, molecule shape, or interaction with other proteins.

### Gene Ontology term enrichment analysis for plasma-and-urine subproteome against Input-plasma proteome

The kidney has been shown to regulate the concentration of many biologically active proteins in the plasma and to play an important role in the disposal of circulating small proteins [26]–[30]. We believe that the functions of the overrepresented proteins in the plasma-and-urine subproteome are the major functions that can be routinely regulated by excretion from the kidney in physiological conditions. The Gene Ontology project provides a controlled vocabulary for describing a protein in terms of its molecular function, biological process, or subcellular localization [31]. We used the Biological Networks Gene Ontology (BiNGO) program package to search for GO terms statistically overrepresented in the plasma-and-urine subproteome versus the whole plasma proteome [22]. The plasma proteins (2674 proteins) were regarded as the reference dataset in the enrichment analysis. A custom GO annotation file for the reference dataset was created with the instructions on the BiNGO webpage (http://www.psb.ugent.be/cbd/papers/BiNGO).

**Figure 4:**
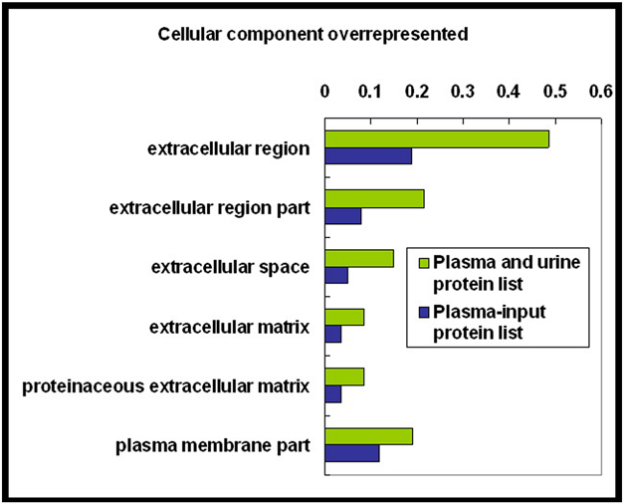
Significantly overrepresented GO cellular component terms for the set of plasma-and-urine proteins. The plasma-and-urine proteins were compared with the entire list of plasma proteins (2674 proteins), and significantly over-represented GO terms (P<0.001) are shown. The ratio shown is the number of plasma-and-urine proteins and total plasma proteins annotated for each GO term divided by the number of plasma-and-urine proteins and total plasma proteins linked to at least one annotated term within the indicated GO cellular component, molecular function, and biological process categories. GO, Gene Ontology.

**Figure 5:**
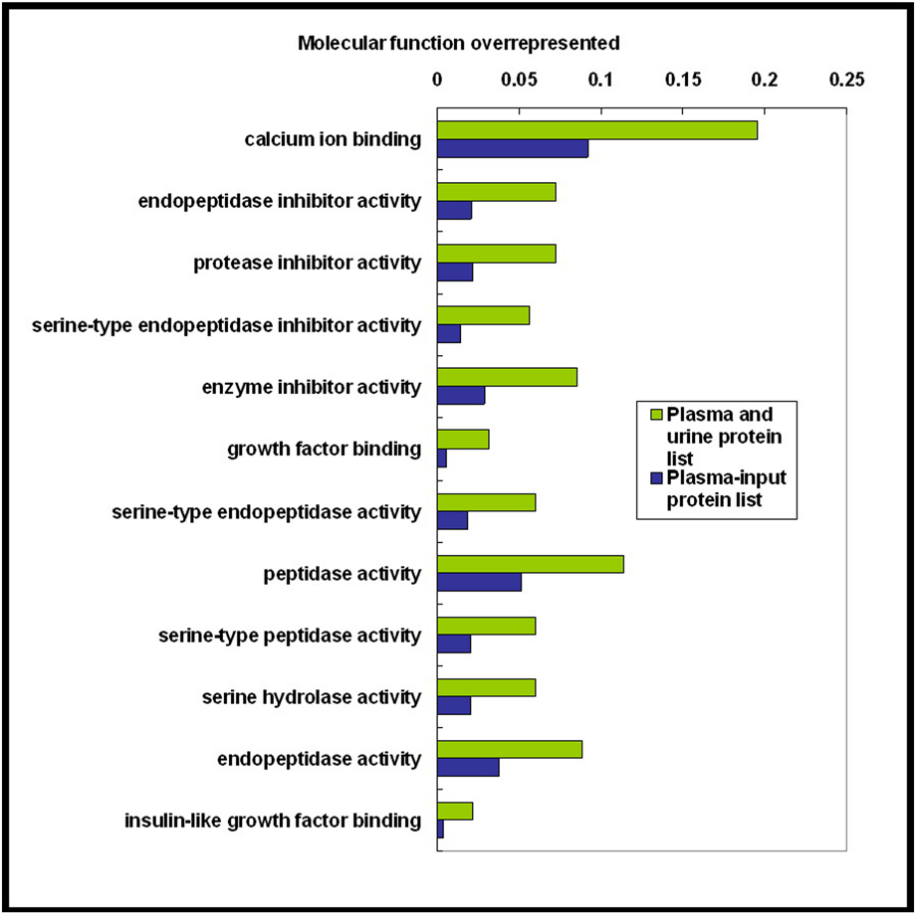
Significantly overrepresented GO molecular function terms for the set of plasma-and-urine proteins. Each term was selected as described in Figure 4. GO, Gene Ontology.

**Figure 6:**
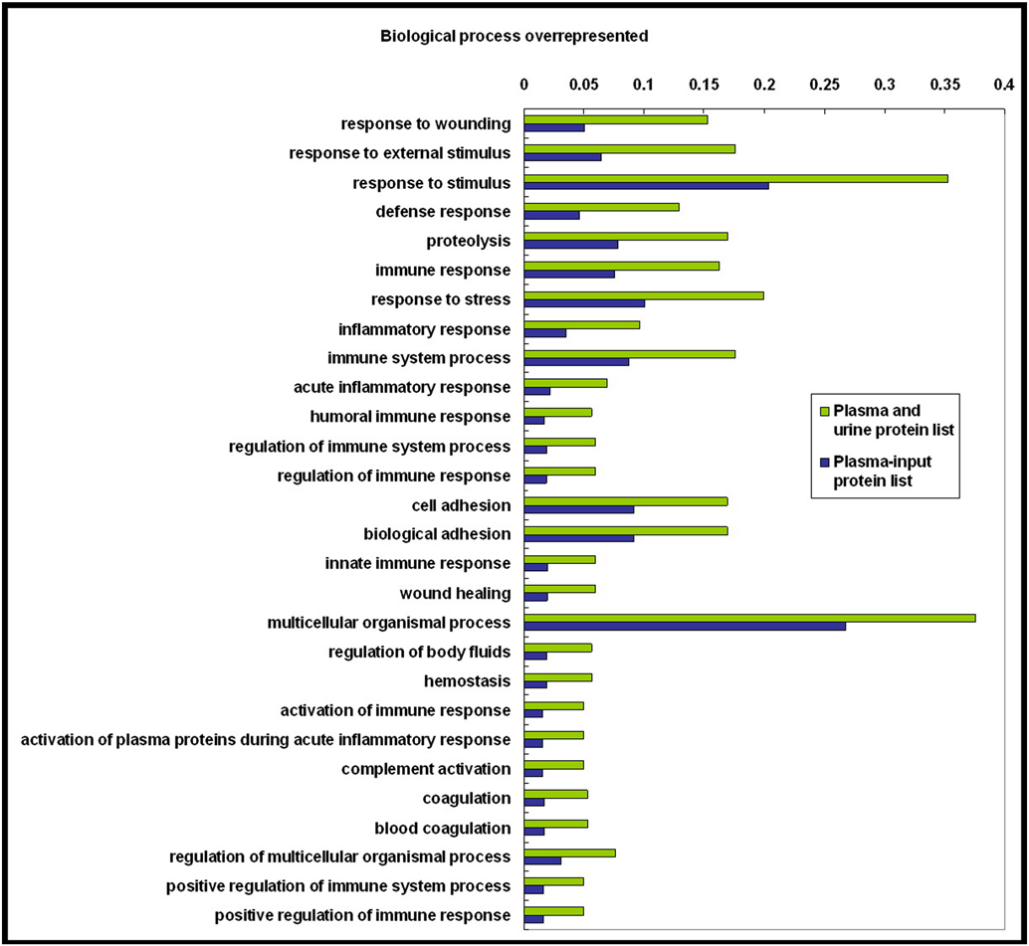
Significantly overrepresented GO biological process terms for the set of plasma-and-urine proteins. Each term was selected as described in Figure 4. GO, Gene Ontology.

As shown in Figure 4, in the cellular component category, six GO terms were overrepresented, they were the extracellular region, the extracellular region part, the extracellular space, the extracellular matrix, the proteinaceous extracellular matrix, and the plasma membrane part. All of them were enriched in the ‘core dataset’ plasma proteome at the same significance level [32]. This suggests that many extracellular proteins and plasma membrane proteins in plasma pass through the kidney into the urine and that these proteins were probably eliminated by excretion into the urine. On the other hand, many proteins whose functions were associated with the nucleus, cytoskeleton, intermediate filaments, and collagen were overrepresented in the ‘core dataset’ of the plasma proteome [32] but were not enriched in the plasma-and-urine proteome. In the molecular function category (Figure 5), 12 GO terms were enriched in the plasma-and-urine proteome, with nine terms emphasizing the enzyme activity or enzyme inhibitor activity categories and three that include calcium ion binding, growth factor binding, and insulin-like growth factor binding. The GO terms enrichment analysis suggested that many proteins related to enzyme regulation, calcium ion binding, and growth factor binding in plasma were also excreted. This could be a mechanism for regulation of the functions of these proteins in the human body. For terms related to biological processes (Figure 6), 28 terms were overrepresented in the plasma-and-urine proteome, including those associated with response to stimulus, the immune system, stress responses, inflammation, wound repair, and coagulation. These biological processes probably could be regulated and affected by kidney function too.

There were many proteins enriched in the plasma-and-urine proteome. There might be a regulatory mechanism performed by the kidney to quickly and precisely regulate the quantity of these proteins within a narrow range in plasma. The regulation may be vital to the survival of the body. Therefore, the quantity of these proteins in the urine can vary to keep homeostasis in the plasma. Since their quantities probably change constantly under physiological conditions, they might not be good candidates for disease biomarkers.

### Discussion and perspective

Since mass spectrometry-based proteomics was founded, body fluid proteomes, such as plasma, urine, tear, and cerebrospinal fluid, have been profiled by many groups. However, all of the body fluids interact with each other and many organs and collectively contribute to form a dynamic system in the body. For instance, plasma may influence most other body fluids, such as urine, cerebrospinal fluid, and tears. It is important to analyze the proteomes of various body fluids in the context of plasma. Normal urinary proteins were considered to reflect normal kidney physiology because the urinary proteome contains not only plasma proteins but also kidney proteins [8]–[14], [33]. Pisitkun et al thought Urinary proteins include glomerular filtrated plasma proteins, soluble proteins secreted by epithelial cells, and solid phase elements of epithelial cells and other cell sources and exosomes.[15] Another study of urine collected from normal human adult subjects indicated that ∼48% of the total urinary proteins excreted was contained in sediments, 49% was soluble, and the remaining 3% was in exosomes [34]. Based on the analysis above, the plasma-only subproteome was derived mainly from the soluble proteins that could not pass the kidney and solid phase components in the plasma; the plasma-and-urine subproteome was derived mainly from glomerular filtration of plasma proteins and epithelial cell secretion of soluble proteins; the urine-only subproteome was derived mainly from the epithelial cell secretion of soluble proteins and solid phase components in urine.

Proteins in the plasma-and-urine subproteome were excreted into urine, which constitutes a loss of substance. As an organ that serves to keep the homeostasis of the internal environment, the kidney's function of excreting some proteins from plasma is well conserved during evolution. By Gene Ontology enrichment analysis, we found that many enzymes, enzyme inhibitors, calcium and growth factor binding proteins, and proteins involved in other biological processes were excreted by the kidney. This suggests that plasma levels and/or plasma half-lives of these proteins can be regulated by the kidney. For instance, proteases are important to biological functions in the plasma. Both excess and deficiency of them are vital to the survival of the body. They need to be regulated quickly and precisely in the plasma, even at the cost of substance loss.

Though proteomics has been improving rapidly, it is probably still far from being capable of exhaustively identifying proteins in plasma and urine. Here, the comparison method described in this paper provides an illustration of a new approach for studying organ functions with a proteomics methodology. In the future, plasma and urine samples from one individual at the same time point can be characterized for the study of an individual's kidney function. Sex specific proteins, presumably coming from sex specific glands, can be identified if the male and female proteomes are profiled separately and they should be removed from the kidney output proteome for the black box study of kidney function. It would be better to compare the two proteomes using unbiased quantitative proteomics techniques. However, such studies are not available yet. With further development of proteomics technologies, i.e., quantitative-MS-based proteomics, top-down strategy proteomics, and antibody arrays, and improvement in the data quality, such comparisons will presumably result in more meaningful and valid conclusions. More detailed descriptions of kidney functions can be obtained by comparing two or more proteomes with more exhaustive and reliable protein information, such as complete MWs, pIs, posttranslational modifications, and quantitation. Because of its distinctive input (plasma) and output (urine), it is reasonable to predict that the kidney will be the first organ whose functions are further elucidated by proteomic methods in the near future. It can also be anticipated that there will be more applications for proteomics in organ function research.

## Supporting Information

Table S1 Plasma-only subproteome
(0.93 MB PDF)

Table S2 Plasma-and-Urine subproteome
(0.22 MB PDF)

Table S3 Urine-only subproteome
(0.49 MB PDF)
