# Supplementary material for: An Attempt to Understand Kidney's Protein Handling Function by Comparing Plasma and Urine Proteomes
Source: PLoS One. 2009 Apr 20;4(4):e5146. doi: 10.1371/journal.pone.0005146 (PMC2668176; doi:10.1371/journal.pone.0005146)
Supplement: Table S1 — Plasma-only subproteome (0.93 MB PDF) [file pone.0005146.s001.pdf]

Table S1 Plasma-only subproteome

IPI3.24	TheoMW(kD)	ExpMW_pla(kD)	(ExpMW_pla-TheoMW)/TheoMW	Proteins Name
IPI00000026	53.1	51.2	-3.58%	Isoform 1 of Calcium/calmodulin-dependent protein kinase type 1
IPI00004373	26.1	26.3	0.77%	Mannose-binding protein C precursor
IPI00004433	114	127.6	11.93%	Contactin-6 precursor
IPI00005439	42.1	51.5	22.33%	Fetuin-B precursor
IPI00006154	30.7	35	14.01%	Isoform Long of Complement factor H-related protein 2 precursor
IPI00007199	55.1	70.8	28.49%	Protein Z-dependent protease inhibitor precursor
IPI00008616	119.1	112.1	-5.88%	Isoform 1 of Solute carrier family 12 member 7
IPI00010295	52.3	42.5	-18.74%	Carboxypeptidase N catalytic chain precursor
IPI00011252	65.2	58.7	-9.97%	Complement component C8 alpha chain precursor
IPI00011933	113.4	135.2	19.22%	Isoform 1 of Centrosomal protein of 110 kDa
IPI00014572	34.6	38.8	12.14%	SPARC precursor
IPI00016076	56.4	80.1	42.02%	Growth-arrest-specific protein 8
IPI00017303	104.7	102.3	-2.29%	DNA mismatch repair protein Msh2
IPI00019576	54.7	47.1	-13.89%	Coagulation factor X precursor
IPI00020019	26.4	26.3	-0.38%	Adiponectin precursor
IPI00020416	138.2	117.8	-14.76%	Tripeptidyl-peptidase 2
IPI00021727	67	65.1	-2.84%	C4b-binding protein alpha chain precursor
IPI00021817	52.1	38.8	-25.53%	Vitamin K-dependent protein C precursor
IPI00021857	10.9	8.4	-22.94%	Apolipoprotein C-III precursor
IPI00022392	26	26.3	1.15%	Complement C1q subcomponent subunit A precursor
IPI00022394	25.8	17.4	-32.56%	Complement C1q subcomponent subunit C precursor
IPI00022445	13.9	6	-56.83%	Platelet basic protein precursor
IPI00022733	44.8	65.1	45.31%	45 kDa protein
IPI00023876	33.3	51.6	54.95%	Isoform Alpha of Caspase-6 precursor
IPI00024214	55.6	91.1	63.85%	Isoform 1 of Telomeric repeat-binding factor 2

IPI00025426	163.8	51.6	-68.50%	Pregnancy zone protein precursor
IPI00025862	28.4	42.5	49.65%	C4b-binding protein beta chain precursor
IPI00027235	158.5	118.1	-25.49%	Isoform 1 of Attractin precursor
IPI00028413	100	113.1	13.10%	Inter-alpha-trypsin inhibitor heavy chain H3 precursor
IPI00029702	115.9	65.1	-43.83%	Isoform 1 of Protein tyrosine kinase 2 beta
IPI00031131	46.5	42.5	-8.60%	Adipocyte plasma membrane-associated protein
IPI00031747	55.8	86.5	55.02%	Isoform 1 of RAC-gamma serine/threonine-protein kinase
IPI00032187	166.6	101	-39.38%	nischarin
IPI00032258	192.8	74.1	-61.57%	Complement C4-A precursor
IPI00032311	53.4	58.8	10.11%	Lipopolysaccharide-binding protein precursor
IPI00041065	62.7	72.7	15.95%	Hyaluronan-binding protein 2 precursor
IPI00064667	56.8	58.7	3.35%	Carnosine dipeptidase 1
IPI00102377	117	90.3	-22.82%	ankyrin repeat domain 27
IPI00107113	88	112.1	27.39%	Isoform 1 of U3 small nucleolar RNA-associated protein 14 homolog A
IPI00152815	37.1	47.8	28.84%	VPPP1921
IPI00165125	84.2	127.6	51.54%	Isoform 1 of Uncharacterized protein C14orf37 precursor
IPI00167941	632.8	20.8	-96.71%	Midasin
IPI00168816	55.1	51.6	-6.35%	Protein FAM44A
IPI00169294	48.3	79	63.56%	Isoform 1 of Ras association domain-containing protein 8
IPI00170865	162.9	93.1	-42.85%	Membrane-associated guanylate kinase-related 3
IPI00217630	129.5	136.6	5.48%	Probable ATP-dependent RNA helicase DHX37
IPI00218055	102	65.1	-36.18%	Isoform 1 of Myocardin
IPI00218732	39.6	42.4	7.07%	Serum paraoxonase/arylesterase 1
IPI00232705	16.2	10.8	-33.33%	similar to H3 histone, family 3B
IPI00239405	796.4	80	-89.95%	Isoform 1 of Nesprin-2
IPI00290332	89.4	102.4	14.54%	Rho GTPase activating protein 10
IPI00293057	40.9	51.5	25.92%	Isoform 2 of Carboxypeptidase B2 precursor
IPI00293925	32.9	28.4	-13.68%	Isoform 1 of Ficolin-3 precursor
IPI00294004	75.1	74.1	-1.33%	Vitamin K-dependent protein S precursor

IPI00294744	73.7	58.9	-20.08%	Poly(A)-specific ribonuclease PARN
IPI00296176	51.8	47.1	-9.07%	Coagulation factor IX precursor
IPI00297241	254.4	39.1	-84.63%	Nucleolar preribosomal-associated protein 1
IPI00299778	39.6	38.8	-2.02%	Serum paraoxonase/lactonase 3
IPI00302151	96.4	118.1	22.51%	Coiled-coil domain containing 87
IPI00303963	83.3	86.5	3.84%	Complement C2 precursor (Fragment)
IPI00305868	47.9	47.1	-1.67%	GRAM domain-containing protein 3
IPI00307549	97.8	70.9	-27.51%	ArAchidonAte lipoxygenAse 3
IPI00328409	115.7	112.1	-3.11%	Armadillo repeat-containing protein 4
IPI00329650	34.8	38.8	11.49%	Nucleoporin NUP53
IPI00337696	169.6	118	-30.42%	Isoform 3 of Rap guanine nucleotide exchange factor 6
IPI00398994	213.7	136.5	-36.13%	KIAA1305 protein
IPI00431656	24	29.2	21.67%	Isoform 2 of Alpha-1-antichymotrypsin precursor
IPI00654888	71.7	80.5	12.27%	Kallikrein B, plasma (Fletcher factor) 1
IPI00015614	32.5	20.8	-36%	Isoform A of Trypsin-3 precursor
IPI00000015	56.7	×	×	Splicing factor, arginine/serine-rich 4
IPI00000030	70	×	×	Isoform Delta-1 of Serine/threonine-protein phosphatase 2A 56 kDa regulatory subunit delta isoform
IPI00000075	44.3	×	×	Transforming growth factor beta-1 precursor
IPI00000137	34	×	×	N-acetylglucosamine-1-phosphotransferase subunit gamma precursor
IPI00000138	50.9	×	×	Alpha-1,3-mannosyl-glycoprotein 2-beta-N-acetylglucosaminyltransferase
IPI00000149	55.4	×	×	Isoform 1 of Caspase-8 precursor
IPI00000156	106	×	×	ligase III, DNA, ATP-dependent isoform beta precursor
IPI00000264	53.3	×	×	similar to growth inhibition and differentiation related protein 86
IPI00000390	54.9	×	×	Isoform 1 of Noelin-3 precursor
IPI00000425	98.2	×	×	Vacuolar proton translocating ATPase 116 kDa subunit a isoform 2

IPI00000459	24.9	×	×	Transmembrane gamma-carboxyglutamic acid protein 1 precursor
IPI00000514	68.9	×	×	Ankyrin repeat domain 30B
IPI00000643	23.8	×	×	BAG family molecular chaperone regulator 2
IPI00000725	121.5	×	×	Isoform 1 of Zinc finger protein 451
IPI00000728	112.4	×	×	Isoform 1 of Ubiquitin carboxyl-terminal hydrolase 15
IPI00000731	65.1	×	×	Acyloxyacyl hydrolase precursor
IPI00000784	55.9	×	×	Isoform MEF2DAB of Myocyte-specific enhancer factor 2D
IPI00000807	215.4	×	×	Isoform 1 of Myosin-5A
IPI00000812	78.8	×	×	Cyclic nucleotide-gated cation channel alpha 3
IPI00000839	101.9	×	×	Metabotropic glutamate receptor 4 precursor
IPI00000856	77.9	×	×	Isoform 1 of Pleckstrin homology domain-containing family C member 1
IPI00000897	219	×	×	Probable helicase with zinc-finger domain
IPI00000948	49.8	×	×	Transducin beta-like 2 protein
IPI00000958	31.9	×	×	OTTHUMP00000031568
IPI00001022	15.4	×	×	myofibrillogenesis regulator 1 isoform 3
IPI00001091	88.5	×	×	AFG3-like protein 2
IPI00001120	68.1	×	×	OTTHUMP00000021174
IPI00001159	292.9	×	×	GCN1-like protein 1
IPI00001233	23.7	×	×	Isoform 4 of Tripartite motif-containing protein 7
IPI00001245	140.9	×	×	Isoform 1 of Contactin-associated protein-like 3 precursor
IPI00001429	87.3	×	×	Protocadherin beta 4 precursor
IPI00001433	86.3	×	×	Protocadherin beta 15 precursor
IPI00001458	250.7	×	×	Kinetochore-associated protein 1
IPI00001461	21.5	×	×	Putative testis protein CYorf15A
IPI00001527	17.5	×	×	Isoform 1 of Protein FAM107A
IPI00001557	99.7	×	×	Sodium/hydrogen exchanger 5
IPI00001690	191.2	×	×	Cullin-7
IPI00001712	99.8	×	×	Isoform 1 of Catenin alpha-3

IPI00001726	113.4	×	×	similar to Ubiquitin carboxyl-terminal hydrolase 35 (Ubiquitin thioesterase 35) (Ubiquitin-specific-processing protease 35) (Deubiquitinating enzyme 35) isoform 1
IPI00001730	126.4	×	×	FH1/FH2 domain-containing protein
IPI00001735	274.7	×	×	nuclear receptor co-repressor 2 isoform 1
IPI00001758	80.9	×	×	ODF2 protein
IPI00001780	93.6	×	×	Ubiquitin carboxyl-terminal hydrolase 16
IPI00001790	47.7	×	×	CDNA FLJ12906 fis, clone NT2RP2004373
IPI00001835	105.1	×	×	Zinc finger and BTB domain-containing protein 4
IPI00001871	36.6	×	×	PRKC apoptosis WT1 regulator protein
IPI00001885	52.6	×	×	Sorting nexin-8
IPI00001890	98.4	×	×	98 kDa protein
IPI00001893	116.1	×	×	Isoform A of Protocadherin-7 precursor
IPI00002070	94.2	×	×	Leucine-rich repeat-containing protein 8A
IPI00002127	496.4	×	×	Heat shock regulated-1
IPI00002135	90.4	×	×	Transforming acidic coiled-coil-containing protein 3
IPI00002180	100.3	×	×	Protein KIAA1383
IPI00002221	231.1	×	×	KIAA0467 protein
IPI00002236	43.1	×	×	Lactadherin precursor
IPI00002255	319.2	×	×	Lipopolysaccharide-responsive and beige-like anchor protein
IPI00002265	47.3	×	×	Isoform 1 of Homeobox-containing protein 1
IPI00002270	51.2	×	×	UPF0364 protein C6orf211
IPI00002272	187.3	×	×	similar to CG18631-PA isoform 2
IPI00002313	115.9	×	×	Caspase recruitment domain-containing protein 10
IPI00002335	347.9	×	×	Huntingtin
IPI00002347	78.1	×	×	IMP dehydrogenase/GMP reductase family protein
IPI00002349	76.1	×	×	Nuclear fragile X mental retardation-interacting protein 2
IPI00002353	147.5	×	×	147 kDa protein
IPI00002466	28.2	×	×	Hypothetical protein LQFBS-1
IPI00002478	87.2	×	×	Isoform B of Endothelin-converting enzyme 1

IPI00002502	20.6	×	×	Isoform 1 of Cyclic AMP-dependent transcription factor ATF-3
IPI00002545	52.4	×	×	Paraneoplastic neuronal antigen MA3
IPI00002606	80.5	×	×	Isoform 1 of Adseverin
IPI00002649	67	×	×	Isoform 2 of Pinin
IPI00002689	112.1	×	×	Isoform Alpha of Short transient receptor potential channel 4
IPI00002803	104	×	×	Serine/threonine-protein kinase N1
IPI00002804	112	×	×	Serine/threonine-protein kinase N2
IPI00002976	38.9	×	×	Cyclin-G2
IPI00003057	75.8	×	×	MICAL-like 2 isoform 2
IPI00003091	125.8	×	×	Isoform 1 of Protein phosphatase 1 regulatory subunit 3A
IPI00003166	60.9	×	×	Cytosolic phospholipase A2 gamma precursor
IPI00003323	131.2	×	×	dentin sialophosphoprotein preproprotein
IPI00003351	60.7	×	×	Extracellular matrix protein 1 precursor
IPI00003367	41.5	×	×	Transcription factor NF-E2 45 kDa subunit
IPI00003384	329.5	×	×	Isoform 1 of Cadherin EGF LAG seven-pass G-type receptor 1 precursor
IPI00003391	305.2	×	×	305 kDa protein
IPI00003515	227.6	×	×	Thyroid receptor-interacting protein 11
IPI00003531	115.1	×	×	Immunoglobulin superfamily member 2 precursor
IPI00003627	47.5	×	×	Isoform 1 of Actin-like protein 6A
IPI00003783	44.4	×	×	Dual specificity mitogen-activated protein kinase kinase 2
IPI00003843	134	×	×	Isoform A1 of Tight junction protein ZO-2
IPI00003847	61.1	×	×	Zinc finger protein 324
IPI00003848	37.8	×	×	DnaJ homolog subfamily B member 4
IPI00003909	53.9	×	×	Solute carrier family 2, facilitated glucose transporter member 3
IPI00003926	32.8	×	×	Protein CLN8
IPI00003951	14.9	×	×	15 kDa protein
IPI00003964	290.5	×	×	ubiquitin specific protease 9, X-linked isoform 4
IPI00003983	51.1	×	×	T-cell surface glycoprotein CD4 precursor

IPI00004065	35.9	×	×	Ecto-ADP-ribosyltransferase 4 precursor
IPI00004068	247.3	×	×	Mediator of RNA polymerase II transcription subunit 12
IPI00004092	114	×	×	Isoform SERCA3B of Sarcoplasmic/endoplasmic reticulum calcium ATPase 3
IPI00004121	43.8	×	×	Isoform 2 of Protein Wnt-2b precursor
IPI00004233	358.7	×	×	Isoform Long of Antigen KI-67
IPI00004288	51.1	×	×	Isoform 1 of Sialic acid-binding Ig-like lectin 7 precursor
IPI00004325	66.2	×	×	Isoform 1 of TAF5-like RNA polymerase II p300/CBP-associated factor-associated factor 65 kDa subunit 5L
IPI00004344	127.5	×	×	Isoform 1 of AF4/FMR2 family member 4
IPI00004389	76.1	×	×	Gamma-tubulin complex component 4
IPI00004450	42.8	×	×	testes-specific heterogenous nuclear ribonucleoprotein G-T
IPI00004457	84.5	×	×	Membrane copper amine oxidase
IPI00004489	49.7	×	×	Molybdenum cofactor synthesis protein 3
IPI00004494	89.2	×	×	Semaphorin-3E precursor
IPI00004497	142.8	×	×	Breakpoint cluster region protein
IPI00004527	116	×	×	Protein KIAA0355
IPI00004533	85.1	×	×	Kinesin-like protein KIF3B
IPI00004560	82.2	×	×	Isoform 2 of Serine/threonine-protein kinase DCAMKL1
IPI00004566	71.8	×	×	Tyrosine-protein kinase ITK/TSK
IPI00004670	72.6	×	×	Isoform 1 of Polypeptide N-acetylgalactosaminyltransferase 3
IPI00004671	376.1	×	×	Golgin subfamily B member 1
IPI00004859	159	×	×	Bloom syndrome protein
IPI00004957	53.6	×	×	Angiopoietin-related protein 3 precursor
IPI00004970	318.4	×	×	Small subunit processome component 20 homolog
IPI00005004	187.9	×	×	Zinc finger protein 142
IPI00005030	107.5	×	×	SH3-domain binding protein 4
IPI00005031	112.7	×	×	Isoform 7 of Protocadherin-11 X-linked precursor
IPI00005039	81.3	×	×	Translation initiation factor IF-2, mitochondrial precursor
IPI00005055	25.6	×	×	CDNA: FLJ22965 fis, clone KAT10418

IPI00005087	39.6	×	×	Tropomodulin-3
IPI00005089	39.6	×	×	Tropomodulin-2
IPI00005094	93.7	×	×	Isoform 1 of Protein FAM35A
IPI00005240	115.2	×	×	Isoform 2 of Nuclear factor of activated T-cells, cytoplasmic 3
IPI00005270	58	×	×	KDEL motif-containing protein 1 precursor
IPI00005516	72.7	×	×	Leucine-rich repeat-containing protein 4 precursor
IPI00005530	22.3	×	×	FMRFamide-related peptides precursor
IPI00005577	116.7	×	×	Isoform 1 of Interferon-induced helicase C domain-containing protein 1
IPI00005607	88.8	×	×	Isoform 1 of Protein FAM5A precursor
IPI00005631	184.7	×	×	Isoform 1 of GRIP and coiled-coil domain-containing protein 2
IPI00005634	175.5	×	×	Tetratricopeptide repeat protein KIAA0372
IPI00005648	107.5	×	×	Scaffold attachment factor B2
IPI00005667	100.4	×	×	Nedd4 binding protein 1
IPI00005686	56.8	×	×	Isoform 1 of Endothelial lipase precursor
IPI00005698	135.3	×	×	Isoform 1 of Regulating synaptic membrane exocytosis protein 2
IPI00005715	146.2	×	×	Isoform 1 of Ubiquitin conjugation factor E4 B
IPI00005743	95.5	×	×	Isoform a of Ataxin-7
IPI00005751	62.9	×	×	Serine palmitoyltransferase 2
IPI00005760	64.4	×	×	TNF receptor-associated factor 5
IPI00005774	105.7	×	×	Isoform 1 of Low-density lipoprotein receptor-related protein 8 precursor
IPI00005793	119.1	×	×	AP-3 complex subunit beta-2
IPI00005811	163.7	×	×	Isoform 1 of DNA mismatch repair protein Mlh3
IPI00005826	527.5	×	×	HECT domain and RCC1-like domain-containing protein 2
IPI00005858	126.9	×	×	Isoform 1 of Serine/threonine-protein kinase LATS1
IPI00005859	59.6	×	×	Cytokeratin type II
IPI00005861	77.5	×	×	Isoform 1 of U4/U6 small nuclear ribonucleoprotein Prp3

IPI00005979	57.6	×	×	Isoform 1 of Translation initiation factor eIF-2B subunit delta
IPI00006014	104.4	×	×	Isoform 1 of Protein KIAA0562
IPI00006025	109.9	×	×	Isoform 1 of Squamous cell carcinoma antigen recognized by T-cells 3
IPI00006056	47.5	×	×	Guanine nucleotide-binding protein-like 1
IPI00006073	63.7	×	×	Isoform Long of Tumor necrosis factor receptor superfamily member 8 precursor
IPI00006084	96.7	×	×	Cyclin M2
IPI00006093	240.9	×	×	Mib
IPI00006096	169.3	×	×	Protein KIAA0586
IPI00006108	108.1	×	×	Mastermind-like protein 1
IPI00006122	91	×	×	Isoform MEA6 of Cutaneous T-cell lymphoma-associated antigen 5
IPI00006143	121	×	×	KIAA0013 protein (Fragment)
IPI00006167	59.3	×	×	Protein phosphatase 2C isoform gamma
IPI00006196	236.5	×	×	Isoform 2 of Nuclear mitotic apparatus protein 1
IPI00006379	59.6	×	×	Nucleolar protein NOP5
IPI00006451	82.7	×	×	Vesicle-fusing ATPase
IPI00006543	67.4	×	×	Complement factor H-related 5
IPI00006620	57.6	×	×	Monocarboxylate transporter 7
IPI00006631	77.4	×	×	Synaptic vesicle glycoprotein 2B
IPI00006640	46.1	×	×	Serpin I2 precursor
IPI00006645	188.5	×	×	similar to DENN/MADD domain containing 4B isoform 1
IPI00006663	56.4	×	×	Aldehyde dehydrogenase, mitochondrial precursor
IPI00006674	169.3	×	×	Isoform 3 of Canalicular multispecific organic anion transporter 2
IPI00006675	149.5	×	×	Multidrug resistance-associated protein 4
IPI00006680	116.9	×	×	family with sequence similarity 13, member A1 isoform a
IPI00006714	275.6	×	×	Isoform 3 of Tyrosine-protein phosphatase non-receptor type 13
IPI00006737	38.9	×	×	Melanoma-associated antigen B4

IPI00006854	128.6	×	×	Ras GTPase-activating protein nGAP
IPI00006863	32.8	×	×	Sperm acrosomal protein
IPI00006914	39.6	×	×	Cysteinyl leukotriene receptor 2
IPI00006987	96.3	×	×	ATP-dependent RNA helicase DDX24
IPI00007032	112.2	×	×	Sal-like protein 4
IPI00007043	71.4	×	×	Zinc finger protein 221
IPI00007122	152.8	×	×	Chromosome 6 open reading frame 84
IPI00007127	53.4	×	×	Isoform 1 of Dipeptidase 2 precursor
IPI00007133	127.9	×	×	Isoform 3 of Cordon-bleu protein-like 1
IPI00007193	148.3	×	×	Isoform 2 of Ankyrin repeat domain-containing protein 26
IPI00007240	75.5	×	×	Coagulation factor XIII B chain precursor
IPI00007253	160.9	×	×	Isoform 1 of Protein TRS85 homolog
IPI00007277	82.2	×	×	Isoform 1 of Leucine-rich repeat flightless-interacting protein 2
IPI00007287	55.3	×	×	Isoform 1 of CASP8 and FADD-like apoptosis regulator precursor
IPI00007293	164.3	×	×	centrosomal protein 164kDa
IPI00007343	38.8	×	×	RING finger protein 113A
IPI00007404	78	×	×	Isoform 1 of Autophagy-related protein 7
IPI00007425	93.8	×	×	desmocollin 1 isoform Dsc1b preproprotein
IPI00007577	31.5	×	×	Isoform 1 of Tumor necrosis factor ligand superfamily member 6
IPI00007692	109.7	×	×	ADAMTS-7 precursor
IPI00007810	47.9	×	×	Serine/threonine-protein kinase H1
IPI00007812	56.5	×	×	Vacuolar ATP synthase subunit B, brain isoform
IPI00007818	77.5	×	×	Cleavage and polyadenylation specificity factor 73 kDa subunit
IPI00007834	430.3	×	×	Isoform 1 of Ankyrin-2
IPI00007856	223	×	×	Myosin heavy chain, skeletal muscle, adult 2
IPI00007899	12.2	×	×	Single chain Fv (Fragment)
IPI00007917	30.7	×	×	collectin sub-family member 10
IPI00007928	273.6	×	×	Pre-mRNA-processing-splicing factor 8
IPI00007940	38.9	×	×	SPFH domain-containing protein 1 precursor

IPI00008085	94.1	×	×	Solute carrier family 39 (Zinc transporter), member 10
IPI00008087	95.8	×	×	Follistatin-related protein 5 precursor
IPI00008091	176.8	×	×	Putative DNA helicase INO80 complex homolog 1
IPI00008135	78.2	×	×	Novel protein
IPI00008173	155.4	×	×	Pleckstrin homology domain-containing family G member 1
IPI00008176	164.9	×	×	Novel protein
IPI00008219	39.6	×	×	UV excision repair protein RAD23 homolog A
IPI00008225	61.6	×	×	Gamma-aminobutyric-acid receptor subunit alpha-4 precursor
IPI00008254	74.1	×	×	Isoform OATP1a of Solute carrier organic anion transporter family member 1A2
IPI00008332	122.3	×	×	Myelin transcription factor 1
IPI00008338	164.9	×	×	Isoform 2 of Multidrug resistance-associated protein 1
IPI00008453	53.2	×	×	Coronin-1C
IPI00008455	146	×	×	Isoform 2 of Myosin-6
IPI00008522	220.6	×	×	Sodium channel protein type 10 subunit alpha
IPI00008524	70.7	×	×	Isoform 1 of Polyadenylate-binding protein 1
IPI00008533	67	×	×	Isoform Long of Matrix metalloproteinase-17 precursor
IPI00008556	70.1	×	×	Isoform 1 of Coagulation factor XI precursor
IPI00008665	14.3	×	×	14 kDa protein
IPI00008692	52.2	×	×	Keratin, type I cuticular Ha6
IPI00008708	55	×	×	Ribosomal L1 domain-containing protein 1
IPI00008726	105	×	×	Iron-responsive element-binding protein 2
IPI00008732	42.3	×	×	Isoform 1 of TraB domain-containing protein
IPI00008756	372.2	×	×	Isoform 1 of Bullous pemphigoid antigen 1 isoforms 1/2/3/4/5/8 (Fragment)
IPI00008770	25.6	×	×	glutathione S-transferase M4 isoform 1
IPI00008821	116.8	×	×	HECT domain and RCC1-like domain-containing protein 5
IPI00008867	81	×	×	Glycogen [starch] synthase, liver
IPI00008868	270.6	×	×	Microtubule-associated protein 1B
IPI00008887	94.6	×	×	Toll-like receptor 10 precursor

IPI00008917	52.8	×	×	Isoform 1 of Doublecortin domain-containing protein 2
IPI00008918	85.2	×	×	Isoform Beta of LIM domain and actin-binding protein 1
IPI00008943	53.9	×	×	Isoform 1 of ATP-dependent RNA helicase DDX19B
IPI00008961	44.3	×	×	Telomeric repeat-binding factor 2-interacting protein 1
IPI00009019	25.7	×	×	Putative insulin-like growth factor 2 antisense gene protein
IPI00009243	132.5	×	×	Isoform B of Leptin receptor precursor
IPI00009301	29.6	×	×	Isoform 1 of COP9 signalosome complex subunit 7b
IPI00009303	65.3	×	×	DNA-binding protein RFX5
IPI00009329	394.5	×	×	Utrophin
IPI00009371	56	×	×	Inositol-pentakisphosphate 2-kinase
IPI00009410	104.7	×	×	ER degradation-enhancing alpha-mannosidase-like 3
IPI00009415	26.1	×	×	Isoform 1 of Rab-like protein 2B
IPI00009447	84.1	×	×	phosphatidylinositol glycan anchor biosynthesis, class Q isoform 1
IPI00009524	55	×	×	Isoform Alpha of Tripartite motif-containing protein 10
IPI00009631	38.6	×	×	Isoform 1 of CMP-N-acetylneuraminate-beta-galactosamide-alpha-2,3-sialyltransferase
IPI00009747	83.3	×	×	Lanosterol synthase
IPI00009792	13	×	×	Ig heavy chain V-I region V35 precursor
IPI00009865	59.5	×	×	Keratin, type I cytoskeletal 10
IPI00009866	49.6	×	×	Isoform 1 of Keratin, type I cytoskeletal 13
IPI00009867	62.4	×	×	Keratin, type II cytoskeletal 5
IPI00009904	72.9	×	×	Protein disulfide-isomerase A4 precursor
IPI00009917	76.8	×	×	Isoform 3 of LAS1-like protein
IPI00009946	34.6	×	×	Mitochondrial import receptor subunit TOM34
IPI00009958	37.4	×	×	COP9 signalosome complex subunit 5
IPI00010134	78.7	×	×	Solute carrier organic anion transporter family member 1C1
IPI00010195	78.1	×	×	axotrophin
IPI00010196	126.9	×	×	Nuclear receptor-interacting protein 1
IPI00010200	160.2	×	×	YTH domain containing 2

IPI00010208	31	×	×	Uncharacterized bone marrow protein BM044
IPI00010360	161.7	×	×	Isoform 1 of Collagen alpha-3(IV) chain precursor
IPI00010365	88.2	×	×	similar to Ubiquitin carboxyl-terminal hydrolase 22 (Ubiquitin thioesterase 22) (Ubiquitin-specific-processing protease 22) (Deubiquitinating enzyme 22) isoform 1
IPI00010369	315.4	×	×	Testis-expressed sequence 15 protein
IPI00010448	73.4	×	×	Rho GTPase activating protein 24 isoform 2
IPI00010486	127.4	×	×	Isoform 1 of Hermansky-Pudlak syndrome 5 protein
IPI00010540	57.3	×	×	Isoform 1 of Sestrin-3
IPI00010544	61.8	×	×	Isoform 1 of Tight junction-associated protein 1
IPI00010575	53.5	×	×	Ribonuclease H domain containing protein
IPI00010590	97.1	×	×	Isoform 1 of Lymphoid-specific helicase
IPI00010604	259.9	×	×	Isoform 1 of 1-phosphatidylinositol-4,5-bisphosphate phosphodiesterase epsilon 1
IPI00010692	131.9	×	×	Period circadian protein homolog 3
IPI00010728	202.9	×	×	203 kDa protein
IPI00010779	28.5	×	×	Tropomyosin 4
IPI00010800	176.7	×	×	Nestin
IPI00010855	20.2	×	×	Putative Metabotropic Glutamate Receptor 8
IPI00010920	9	×	×	RU2AS
IPI00010951	553.1	×	×	Epiplakin
IPI00011031	39.3	×	×	Protein Wnt-7b precursor
IPI00011138	54.7	×	×	Isoform 1 of Regulator of nonsense transcripts 3A
IPI00011268	32.6	×	×	RNA binding protein (Fragment)
IPI00011283	171.8	×	×	Isoform 1 of Collagen alpha-2(XI) chain precursor
IPI00011307	37.9	×	×	Bifunctional methylenetetrahydrofolate dehydrogenase/cyclohydrolase, mitochondrial precursor
IPI00011365	60	×	×	Isoform 1 of Tripartite motif-containing protein 39
IPI00011370	9.8	×	×	PRO1102
IPI00011400	177.6	×	×	T-lymphoma invasion and metastasis-inducing protein 1

IPI00011569	265.6	×	×	Acetyl-CoA carboxylase 1
IPI00011603	61	×	×	26S proteasome non-ATPase regulatory subunit 3
IPI00011633	133.4	×	×	133 kDa protein
IPI00011635	52.7	×	×	Isoform 2 of Bcl-2-like 13 protein
IPI00011652	58.8	×	×	Isoform Efs1 of Embryonal Fyn-associated substrate
IPI00011685	66.2	×	×	Collagen alpha-1(X) chain precursor
IPI00011692	68.5	×	×	Involucrin
IPI00011749	177.9	×	×	similar to human immunodeficiency virus type I enhancer binding protein 3
IPI00011832	24.3	×	×	Secreted phosphoprotein 24 precursor
IPI00011875	65.6	×	×	Protein Red
IPI00011938	130.6	×	×	Isoform 1 of Adenylate cyclase type 6
IPI00011981	64.4	×	×	Solute carrier family 13 member 2
IPI00012074	70.9	×	×	Heterogeneous nuclear ribonucleoprotein R
IPI00012190	61.3	×	×	Putative dimethylaniline monooxygenase [N-oxide-forming] 6
IPI00012213	113.3	×	×	Caspase recruitment domain-containing protein 14
IPI00012347	312.3	×	×	Matrix-remodelling-associated protein 5 precursor
IPI00012363	132.4	×	×	Isoform Alpha of Metabotropic glutamate receptor 1 precursor
IPI00012391	17.8	×	×	APC protein (Fragment)
IPI00012463	33.6	×	×	TatD DNase domain containing 1
IPI00012465	41.6	×	×	Testis-specific serine/threonine-protein kinase 1
IPI00012495	47.8	×	×	Homeobox even-skipped homolog protein 2
IPI00012500	140.9	×	×	Isoform 1 of Fanconi anemia group J protein
IPI00012510	115.6	×	×	EMILIN-2 precursor
IPI00012541	89.2	×	×	Isoform 2 of Protocadherin gamma A6 precursor
IPI00012578	57.9	×	×	Importin alpha-4 subunit
IPI00012622	132.6	×	×	Rho GTPase activating protein 20
IPI00012733	41.5	×	×	C-X-C chemokine receptor type 7
IPI00012749	50.8	×	×	Isoform 1 of Protein C20orf54 precursor
IPI00012792	87.5	×	×	Cadherin-5 precursor

IPI00012829	42.2	×	×	DNA repair protein RAD51 homolog 3
IPI00012837	109.7	×	×	Kinesin heavy chain
IPI00012851	143.7	×	×	Probable phospholipid-transporting ATPase IC
IPI00012857	96.7	×	×	Potassium voltage-gated channel subfamily KQT member 3
IPI00012912	73.8	×	×	Carnitine O-palmitoyltransferase 2, mitochondrial precursor
IPI00013010	98.9	×	×	NEDD4-like E3 ubiquitin-protein ligase WWP2
IPI00013076	43.1	×	×	Isoform 1 of FGFR1 oncogene partner
IPI00013079	106.7	×	×	EMILIN-1 precursor
IPI00013096	164.3	×	×	Isoform 1 of Receptor-type tyrosine-protein phosphatase T precursor
IPI00013146	41.3	×	×	Mitochondrial 28S ribosomal protein S22
IPI00013193	254.4	×	×	Isoform 1 of Myosin-7A
IPI00013200	28.2	×	×	C->U-editing enzyme APOBEC-1
IPI00013215	97.3	×	×	Origin recognition complex subunit 1
IPI00013236	30.2	×	×	Isoform 2 of Synaptobrevin-like protein 1
IPI00013272	261.1	×	×	Isoform 1 of Golgin subfamily A member 4
IPI00013303	37.4	×	×	Limbic system-associated membrane protein precursor
IPI00013398	45.6	×	×	EF-hand calcium binding domain 4B
IPI00013409	68.4	×	×	Baculoviral IAP repeat-containing protein 3
IPI00013411	35.7	×	×	Similar to RING finger protein 18
IPI00013467	122.5	×	×	123 kDa protein
IPI00013475	49.9	×	×	Tubulin beta-2A chain
IPI00013721	117	×	×	Serine/threonine-protein kinase PRP4 homolog
IPI00013769	49.5	×	×	Alpha-enolase, lung specific
IPI00013864	25.3	×	×	GRB2-related adapter protein
IPI00013871	90.1	×	×	Ribonucleoside-diphosphate reductase large subunit
IPI00013909	40.5	×	×	Isoform 1 of Apoptosis-inducing factor-like mitochondrion-associated inducer of death
IPI00013949	34.1	×	×	Small glutamine-rich tetratricopeptide repeat-containing protein A

IPI00013976	198.1	×	×	Laminin beta-1 chain precursor
IPI00013986	36.9	×	×	XIAP associated factor-1
IPI00013988	171.6	×	×	Rho-GTPase-activating protein 5
IPI00014235	111.3	×	×	Similar to RAB3 GTPase-activating protein
IPI00014266	79.5	×	×	Isoform 1 of Bromodomain-containing protein 3
IPI00014287	27.9	×	×	folate receptor 3 precursor
IPI00014310	89.7	×	×	Cullin-1
IPI00014374	160.6	×	×	Protein patched homolog 1
IPI00014444	74.1	×	×	Serine active site containing 1
IPI00014491	38.2	×	×	Zinc finger protein 73
IPI00014513	44.7	×	×	Transcriptional repressor protein YY1
IPI00014629	33.3	×	×	Protein kinase-like protein SgK493
IPI00014802	42	×	×	Probable G-protein coupled receptor 85
IPI00014829	322.2	×	×	Cadherin protein
IPI00014845	514.7	×	×	OTTHUMP00000017871
IPI00014852	55.6	×	×	Phosphoglucomutase-like protein 5
IPI00015049	47.5	×	×	Isoform 2 of Repulsive guidance molecule A precursor
IPI00015102	65.1	×	×	Isoform 1 of CD166 antigen precursor
IPI00015133	27.9	×	×	Chymotrypsinogen B precursor
IPI00015145	69.7	×	×	Isoform Alpha of Nuclear receptor coactivator 4
IPI00015170	28.8	×	×	Hypothetical protein (Fragment)
IPI00015180	176.4	×	×	Apical-like protein
IPI00015181	114.6	×	×	Integrin alpha-9 precursor
IPI00015213	52.5	×	×	Death-associated protein kinase 3
IPI00015286	215.4	×	×	Dedicator of cytokinesis protein 1
IPI00015345	358.3	×	×	Cadherin EGF LAG seven-pass G-type receptor 3 precursor
IPI00015346	317.5	×	×	Cadherin EGF LAG seven-pass G-type receptor 2 precursor
IPI00015593	200.9	×	×	201 kDa protein
IPI00015595	60.4	×	×	Isoform 1 of Coiled-coil domain-containing protein 102B
IPI00015696	41.4	×	×	Triggering receptor expressed on myeloid cells-like 2

IPI00015793	160.5	×	×	Isoform 1 of Telomerase-binding protein EST1A
IPI00015826	79.1	×	×	ATP-binding cassette sub-family B member 10, mitochondrial precursor
IPI00015836	79.7	×	×	DCC-interacting protein 13 alpha
IPI00015864	83.5	×	×	2-5A-dependent ribonuclease
IPI00015869	247.2	×	×	Trichohyalin
IPI00015916	86.4	×	×	Bone-derived growth factor (Fragment)
IPI00015963	127.4	×	×	Transient receptor potential cation channel subfamily A member 1
IPI00015974	109.7	×	×	Serine/threonine-protein kinase/endoribonuclease IRE1 precursor
IPI00015980	221.6	×	×	Isoform 2 of Multiple PDZ domain protein
IPI00016007	202.8	×	×	Myosin-5C
IPI00016408	20.4	×	×	CDNA FLJ20457 fis, clone KAT05844
IPI00016472	184.9	×	×	zinc finger CCCH-type containing 13
IPI00016639	68.3	×	×	Protein kinase C, iota type
IPI00016697	55.8	×	×	CDNA FLJ20641 fis, clone KAT02782
IPI00016701	39	×	×	P2Y purinoceptor 14
IPI00016736	148.5	×	×	1-phosphatidylinositol-4,5-bisphosphate phosphodiesterase gamma 1
IPI00016743	61.5	×	×	mannosyl (alpha-1,3-)-glycoprotein beta-1,4-N-acetylglucosaminyltransferase, isoenzyme A
IPI00016758	60.1	×	×	Isoform Alpha-1 of A/G-specific adenine DNA glycosylase
IPI00016780	189	×	×	Predicted retinoblastoma binding protein (RIZ) family protein
IPI00016810	51	×	×	Isoform 1 of Tryptophan 5-hydroxylase 1
IPI00016814	44.6	×	×	Zinc finger protein 670
IPI00016868	91.7	×	×	KIAA0683
IPI00016949	112.3	×	×	Isoform 4 of Electrogenic sodium bicarbonate cotransporter 1
IPI00017030	233.5	×	×	234 kDa protein
IPI00017094	429.3	×	×	Isoform 3 of Lysosomal-trafficking regulator

IPI00017163	175.8	×	×	HECT, C2 and WW domain containing E3 ubiquitin protein ligase 2
IPI00017297	94.6	×	×	Matrin-3
IPI00017305	82.7	×	×	Ribosomal protein S6 kinase alpha-1
IPI00017330	66	×	×	Isoform GRK6A of G protein-coupled receptor kinase 6
IPI00017422	28	×	×	CDNA FLJ13544 fis, clone PLACE1006815
IPI00017423	28.9	×	×	Isoform 2 of Centrosomal protein of 135 kDa
IPI00017443	51	×	×	absent in melanoma 1-like
IPI00017476	55.1	×	×	Isoform EPOR-F of Erythropoietin receptor precursor
IPI00017538	197.6	×	×	Isoform 2 of Separin
IPI00017551	33.3	×	×	Regucalcin
IPI00017592	83.4	×	×	Leucine zipper-EF-hand-containing transmembrane protein 1, mitochondrial precursor
IPI00017603	267	×	×	Coagulation factor VIII precursor
IPI00017640	167.7	×	×	Isoform 1 of Slit homolog 3 protein precursor
IPI00017648	218.6	×	×	Lactase-phlorizin hydrolase precursor
IPI00017800	191.4	×	×	ATP-binding cassette sub-family A member 3
IPI00017841	55.4	×	×	Isoform 1 of Noelin precursor
IPI00017878	16	×	×	CDNA: FLJ20972 fis, clone ADSU01569
IPI00017910	82	×	×	Isoform 8 of Rho-GTPase-activating protein 8
IPI00017972	58.2	×	×	Isoform 1 of Zinc finger protein 703
IPI00018001	28.5	×	×	CDNA: FLJ22378 fis, clone HRC07430
IPI00018002	65.7	×	×	Novel protein
IPI00018077	44.9	×	×	melanoma antigen family A, 11 isoform b
IPI00018087	134.6	×	×	HBV DNAPTP1-transactivated protein B
IPI00018090	69.2	×	×	Intraflagellar transport 74 homolog
IPI00018109	69	×	×	Prostaglandin G/H synthase 2 precursor
IPI00018149	62.4	×	×	Prostate cancer antigen T21
IPI00018219	74.7	×	×	Transforming growth factor-beta-induced protein ig-h3 precursor

IPI00018240	79.9	×	×	CDNA FLJ10498 fis, clone NT2RP2000328
IPI00018258	48.2	×	×	Sperm-associated antigen 4 protein
IPI00018274	134.3	×	×	Isoform 1 of Epidermal growth factor receptor precursor
IPI00018294	218	×	×	Zinc finger protein Rlf
IPI00018349	96.6	×	×	DNA replication licensing factor MCM4
IPI00018352	24.8	×	×	Ubiquitin carboxyl-terminal hydrolase isozyme L1
IPI00018363	192.6	×	×	OTTHUMP00000017471
IPI00018370	200.8	×	×	Isoform 2 of Supervillin
IPI00018803	29.8	×	×	homeobox D12
IPI00018808	194.9	×	×	hypothetical protein LOC55196
IPI00018813	52.7	×	×	Isoform 2 of COP9 signalosome complex subunit 2
IPI00018823	93.5	×	×	Isoform 1 of Enhancer of polycomb homolog 1
IPI00018829	289	×	×	Isoform 1 of Spectrin beta chain, brain 3
IPI00018837	89	×	×	Pleckstrin homology domain containing, family G (With RhoGef domain) member 6
IPI00018861	28	×	×	NKG2D ligand 1 precursor
IPI00018903	30.9	×	×	Ventral anterior homeobox 2
IPI00018946	86	×	×	Pantothenate kinase 4
IPI00018950	66.5	×	×	Hypothetical protein DKFZp667P0924
IPI00019090	115.2	×	×	Collagen alpha-1
IPI00019148	39.2	×	×	Immunoglobulin-binding protein 1
IPI00019172	39.3	×	×	Isoform 1 of SH3-containing GRB2-like protein 3
IPI00019209	85.2	×	×	Semaphorin-3C precursor
IPI00019223	453.7	×	×	Isoform 1 of A-kinase anchor protein 9
IPI00019228	34.8	×	×	Homeobox protein Nkx-3.2
IPI00019242	75.8	×	×	Matrix metalloproteinase-15 precursor
IPI00019359	62.1	×	×	Keratin, type I cytoskeletal 9
IPI00019427	102	×	×	Sec3 isoform 1
IPI00019432	56.6	×	×	Isoform 1 of NGFI-A-binding protein 2
IPI00019472	56.6	×	×	Neutral amino acid transporter B

IPI00019490	80	×	×	Transcription factor E3
IPI00019491	52	×	×	Neuropeptide Y receptor type 5
IPI00019642	115.8	×	×	Isoform 1 of Cytoplasmic linker protein 2
IPI00019977	49.5	×	×	Transcription initiation factor IIE subunit alpha
IPI00019992	87.4	×	×	Myb-related protein A
IPI00019996	115.4	×	×	modulator of estrogen induced transcription isoform b
IPI00020036	70	×	×	Neuronal acetylcholine receptor protein subunit alpha-4 precursor
IPI00020060	90.8	×	×	Sodium/hydrogen exchanger 1
IPI00020134	153	×	×	Son of sevenless homolog 2
IPI00020153	416.4	×	×	Protein bassoon
IPI00020197	40.5	×	×	Alpha-N-acetylneuraminide alpha-2,8-sialyltransferase
IPI00020265	94	×	×	Ankyrin repeat domain-containing protein 20A1
IPI00020356	331.3	×	×	331 kDa protein
IPI00020435	46.7	×	×	Transcription factor SOX-11
IPI00020501	227.3	×	×	Myosin-11
IPI00020546	332.8	×	×	Probable histone-lysine N-methyltransferase ASH1L
IPI00020587	12.9	×	×	PRO2266
IPI00020618	11	×	×	H4 histone family, member L
IPI00020772	30.7	×	×	Isoform 2 of Vascular non-inflammatory molecule 3 precursor
IPI00020899	78.2	×	×	78 kDa protein
IPI00020918	133.7	×	×	AF4/FMR2 family member 3
IPI00021048	234.7	×	×	Isoform 1 of Myoferlin
IPI00021057	120.6	×	×	Isoform 1 of Solute carrier family 12 member 4
IPI00021092	62.7	×	×	Isoform 1 of Protein downstream neighbor of Son
IPI00021146	39.9	×	×	Isoform 1 of Pre-mRNA-splicing factor 18
IPI00021258	41.7	×	×	Isoform B of Arfaptin-1
IPI00021274	111	×	×	Ephrin type-A receptor 8 precursor
IPI00021304	65.9	×	×	Keratin, type II cytoskeletal 2 epidermal
IPI00021363	195.8	×	×	Jumonji/ARID domain-containing protein 1A

IPI00021364	51.3	×	×	Properdin precursor
IPI00021388	239.2	×	×	Thyroid hormone receptor-associated protein complex 240 kDa component
IPI00021428	42.1	×	×	Actin, alpha skeletal muscle
IPI00021435	48.5	×	×	26S protease regulatory subunit 7
IPI00021443	73.8	×	×	Isoform 1 of Polycystic kidney disease 2-like 2 protein
IPI00021537	73.3	×	×	Isoform 1 of Opioid growth factor receptor
IPI00021685	21.1	×	×	ADP-ribosylation factor-like protein 6
IPI00021739	89.7	×	×	PERQ amino acid rich with GYF domain protein 1
IPI00021753	202.7	×	×	Kinesin-like protein KIF13B
IPI00021770	97.5	×	×	Isoform 1 of 3-hydroxy-3-methylglutaryl-coenzyme A reductase
IPI00021786	73.1	×	×	RAF proto-oncogene serine/threonine-protein kinase
IPI00021907	33.1	×	×	Isoform 1 of Myelin basic protein
IPI00021954	206.4	×	×	Golgi-specific brefeldin A-resistance guanine nucleotide exchange factor 1
IPI00022022	103.6	×	×	SAC domain-containing protein 3
IPI00022043	129.8	×	×	Hamartin
IPI00022055	93	×	×	Histone acetyltransferase PCAF
IPI00022086	40.7	×	×	KIAA1325 protein (Fragment)
IPI00022164	173.3	×	×	Isoform 1 of Rho guanine nucleotide exchange factor 12
IPI00022200	343.7	×	×	alpha 3 type VI collagen isoform 1 precursor
IPI00022215	123.6	×	×	Activity-dependent neuroprotector
IPI00022256	49.7	×	×	AP-2 complex subunit mu-1
IPI00022296	109.9	×	×	Mast/stem cell growth factor receptor precursor
IPI00022333	173.5	×	×	Brain-specific angiogenesis inhibitor 1 precursor
IPI00022370	154.2	×	×	Homeobox protein cut-like 2
IPI00022373	47.7	×	×	Similar to Phosphorylation regulatory protein HP-10
IPI00022388	61.9	×	×	Dihydropyrimidinase-related protein 4
IPI00022389	25	×	×	Isoform 1 of C-reactive protein precursor
IPI00022443	68.7	×	×	Alpha-fetoprotein precursor

IPI00022446	10.8	×	×	Platelet factor 4 precursor
IPI00022449	211.9	×	×	Isoform 1 of Dedicator of cytokinesis protein 2
IPI00022450	89	×	×	TBC1 domain family member 5
IPI00022462	84.9	×	×	Transferrin receptor protein 1
IPI00022471	124.6	×	×	minor histocompatibility antigen HA-1
IPI00022479	532.2	×	×	guanine nucleotide exchange factor p532
IPI00022496	124.1	×	×	similar to Protein KIAA0226
IPI00022543	45.3	×	×	GPI-anchor transamidase precursor
IPI00022648	49.2	×	×	Eukaryotic translation initiation factor 5
IPI00022697	40.7	×	×	Developmentally-regulated GTP-binding protein 2
IPI00022731	14.6	×	×	Apolipoprotein C-IV precursor
IPI00022744	110.4	×	×	Isoform 1 of Exportin-2
IPI00022883	47	×	×	AP-3 complex subunit mu-2
IPI00022890	12.4	×	×	Ig lambda chain V region 4A precursor
IPI00022970	265.9	×	×	Nucleoprotein TPR
IPI00023006	42	×	×	Actin, alpha cardiac muscle 1
IPI00023051	239.4	×	×	Alpha-tectorin precursor
IPI00023109	200.6	×	×	Chromodomain-helicase-DNA-binding protein 2
IPI00023151	83.6	×	×	N-acetylated-alpha-linked acidic dipeptidase 2
IPI00023188	66.1	×	×	Ectoderm-neural cortex 1 protein
IPI00023217	564.5	×	×	Isoform 1 of Ryanodine receptor 2
IPI00023234	71.2	×	×	Ubiquitin-like 1-activating enzyme E1B
IPI00023314	38.2	×	×	Inhibin beta C chain precursor
IPI00023339	265.4	×	×	CREB-binding protein
IPI00023340	225.1	×	×	Histone acetyltransferase MYST3
IPI00023344	141.1	×	×	Isoform 1 of Symplekin
IPI00023407	128.2	×	×	Nck-associated protein 1-like
IPI00023410	117.4	×	×	Integrin alpha-8 precursor
IPI00023603	66.8	×	×	Orphan nuclear receptor NR1D1
IPI00023617	118.8	×	×	Zinc finger protein 197

IPI00023635	31.3	×	×	Isoform 1 of Inositol monophosphatase 2
IPI00023664	54.4	×	×	Serine/threonine-protein kinase Chk1
IPI00023672	85.6	×	×	86 kDa protein
IPI00023711	231.6	×	×	Envoplakin
IPI00023736	59.8	×	×	Coronin-2A
IPI00023757	113.4	×	×	Isoform 1 of X-linked retinitis pigmentosa GTPase regulator
IPI00023868	174.2	×	×	Canalicular multispecific organic anion transporter 1
IPI00024007	99.5	×	×	Isoform B of Smoothelin
IPI00024067	191.6	×	×	clathrin heavy chain 1
IPI00024071	57.3	×	×	Isoform Long of Heat shock factor protein 1
IPI00024111	45.4	×	×	RIB43A domain with coiled-coils 2
IPI00024138	12.8	×	×	Ig kappa chain V-III region VH precursor (Fragment)
IPI00024143	59.6	×	×	Aladin
IPI00024252	25	×	×	Fibroblast growth factor 11
IPI00024278	174.3	×	×	Isoform SUR2A of ATP-binding cassette transporter sub-family C member 9
IPI00024295	84.8	×	×	Zinc finger X-linked protein ZXDA
IPI00024299	41.1	×	×	Snurportin-1
IPI00024330	102.6	×	×	Potassium voltage-gated channel subfamily B member 2
IPI00024387	49.3	×	×	Hepatocyte nuclear factor 3-alpha
IPI00024539	83.7	×	×	Protein kinase C epsilon type
IPI00024547	32.9	×	×	Protein C2orf25, mitochondrial precursor
IPI00024568	153	×	×	Glioma tumor suppressor candidate region gene 1 protein
IPI00024579	56.2	×	×	Postreplication repair protein RAD18
IPI00024661	118.3	×	×	Protein transport protein Sec24C
IPI00024704	47.7	×	×	Uronyl 2-sulfotransferase
IPI00024714	156.4	×	×	Isoform 1 of Regulator of G-protein signaling 12
IPI00024726	105.1	×	×	Peripheral plasma membrane protein CASK
IPI00024766	175.7	×	×	Plexin-C1 precursor
IPI00024776	70	×	×	Calmegin precursor

IPI00024801	35.9	×	×	RING finger protein 41
IPI00024802	206.9	×	×	TATA-binding protein-associated factor 172
IPI00024818	120.4	×	×	Hypothetical protein D13S106
IPI00024821	34.6	×	×	26S proteasome non-ATPase regulatory subunit 14
IPI00024970	106.1	×	×	Inner centromere protein
IPI00024975	160.2	×	×	Kinesin-like protein 2
IPI00025087	43.7	×	×	Isoform 1 of Cellular tumor antigen p53
IPI00025158	144.4	×	×	Cohesin subunit SA-1
IPI00025190	243.9	×	×	adenomatosis polyposis coli 2
IPI00025243	53.8	×	×	Isoform Alpha-3L of Glycine receptor subunit alpha-3 precursor
IPI00025279	70.4	×	×	Guanylate cyclase soluble subunit beta-2
IPI00025307	60.2	×	×	Cytochrome P450 27, mitochondrial precursor
IPI00025311	61.7	×	×	Isoform 1 of Breast carcinoma amplified sequence 1
IPI00025327	15.4	×	×	Plasminogen
IPI00025409	89.4	×	×	CDNA FLJ34103 fis, clone FCBBF3007859, moderately similar to Human putative protein B2 mRNA
IPI00025646	70.5	×	×	Actin-related protein 8
IPI00025679	85.2	×	×	85 kDa protein
IPI00025683	87.8	×	×	Isoform 1 of Transforming acidic coiled-coil-containing protein 1
IPI00025702	169.3	×	×	PR domain zinc finger protein 15
IPI00025815	44.7	×	×	TAR DNA-binding protein 43
IPI00025819	94.3	×	×	Isoform I of Calpain-3
IPI00025879	223.1	×	×	Myosin-1
IPI00025880	223.1	×	×	Myosin heavy chain, cardiac muscle beta isoform
IPI00025976	20.8	×	×	Isoform 4 of Receptor-interacting serine/threonine-protein kinase 5
IPI00026058	66.6	×	×	leucine zipper, putative tumor suppressor 1
IPI00026089	145.8	×	×	Splicing factor 3B subunit 1
IPI00026119	56.9	×	×	Ubiquitin-activating enzyme E1

IPI00026197	19.5	×	×	Similar to Ig kappa chain V-IV region STH
IPI00026305	84.6	×	×	Hook homolog 1
IPI00026320	309.4	×	×	Ubiquitin-protein ligase EDD1
IPI00026328	19.2	×	×	Thioredoxin domain-containing protein 12 precursor
IPI00026524	86.2	×	×	Zinc finger protein 700
IPI00026546	25.6	×	×	Platelet-activating factor acetylhydrolase IB subunit beta
IPI00026602	40.5	×	×	HLA class I histocompatibility antigen, B-41 alpha chain precursor
IPI00026665	89.7	×	×	Glutaminyl-tRNA synthetase
IPI00026673	164.3	×	×	Isoform 1 of Homeobox protein cut-like 1
IPI00026697	148.1	×	×	zinc finger protein 258
IPI00026828	79.2	×	×	Isoform 1 of Tripartite motif-containing protein 9
IPI00026900	12.5	×	×	ORF 114
IPI00026904	54.9	×	×	Isoform 1 of Adenylosuccinate lyase
IPI00026940	50.1	×	×	Nucleoporin 50 kDa
IPI00026969	111.1	×	×	Isoform 1 of SEC23-interacting protein
IPI00027034	72.8	×	×	SGT1 protein
IPI00027078	152.9	×	×	Carboxypeptidase D precursor
IPI00027096	33.5	×	×	39S ribosomal protein L19, mitochondrial precursor
IPI00027107	49.9	×	×	Tu translation elongation factor, mitochondrial
IPI00027157	367.6	×	×	Centromere protein F
IPI00027172	161	×	×	Isoform Long of Restin
IPI00027178	87.1	×	×	AP-1 complex subunit gamma-2
IPI00027194	38.7	×	×	Syntaxin-18
IPI00027195	66.9	×	×	Isoform 1 of NADPH oxidase 4
IPI00027202	90.5	×	×	Zinc finger Y-chromosomal protein
IPI00027228	61.9	×	×	Probable glutamyl-tRNA(Gln) amidotransferase subunit B, mitochondrial precursor
IPI00027242	120.1	×	×	Retinal guanylyl cyclase 1 precursor
IPI00027250	105.8	×	×	Gamma-aminobutyric acid type B receptor, subunit 2 precursor

IPI00027280	183.3	×	×	Isoform Beta-2 of DNA topoisomerase 2-beta
IPI00027378	33.3	×	×	Isoform 1 of SAPK substrate protein 1
IPI00027385	77	×	×	Isoform 1 of Regulator of G-protein signaling 9
IPI00027415	114.8	×	×	Isoform 1 of Probable ATP-dependent RNA helicase DHX36
IPI00027507	37.3	×	×	Complement factor H-related protein 3 precursor
IPI00027721	122.7	×	×	Isoform 1 of Alpha platelet-derived growth factor receptor precursor
IPI00027734	170.7	×	×	similar to Cat eye syndrome critical region protein 2 isoform 1
IPI00027778	52.1	×	×	Peroxisomal NADH pyrophosphatase NUDT12
IPI00027801	114	×	×	Hypothetical protein DKFZp434O1826
IPI00027803	96.3	×	×	Isoform 1 of Poly [ADP-ribose] polymerase 9
IPI00027834	64.1	×	×	heterogeneous nuclear ribonucleoprotein L isoform a
IPI00028031	75.2	×	×	Isoform 1 of Very-long-chain specific acyl-CoA dehydrogenase, mitochondrial precursor
IPI00028051	98.5	×	×	Isoform Long of Vacuolar protein sorting-associated protein 41 homolog
IPI00028065	42.9	×	×	Cytoplasmic protein NCK1
IPI00028189	43	×	×	hypothetical protein LOC80000
IPI00028199	27.9	×	×	Homeobox protein GSH-1
IPI00028262	66.9	×	×	KIAA1754 protein (Fragment)
IPI00028264	81.8	×	×	Exocyst complex component 8
IPI00028275	225.5	×	×	Cytoskeleton-associated protein 5
IPI00028347	60.6	×	×	F-box only protein 31
IPI00028493	200.7	×	×	Isoform 1 of Tuberin
IPI00028539	39.1	×	×	Cylicin-2
IPI00028561	109.5	×	×	Kinesin heavy chain isoform 5C
IPI00028564	67.9	×	×	Interferon-induced guanylate-binding protein 1
IPI00028739	83.3	×	×	Isoform 1 of Nephrocystin-1
IPI00028786	462.4	×	×	Isoform 3 of Polycystin-1 precursor

IPI00028915	259.2	×	×	Isoform 1 of Voltage-dependent T-type calcium channel subunit alpha-1H
IPI00028954	218.4	×	×	80 kDa MCM3-associated protein
IPI00028980	170.6	×	×	Protein KIAA0133
IPI00029045	80.5	×	×	Inhibitor of nuclear factor kappa-B kinase epsilon subunit
IPI00029061	42.7	×	×	Selenoprotein P precursor
IPI00029079	76.7	×	×	GMP synthase
IPI00029107	162.5	×	×	Werner syndrome ATP-dependent helicase
IPI00029196	81.9	×	×	Isoform 1 of Protein kinase C theta type
IPI00029343	148.2	×	×	Isoform 1 of Contactin-associated protein-like 2 precursor
IPI00029372	69.7	×	×	Hypothetical protein DKFZp686I1868
IPI00029400	38.2	×	×	Isoform ZIS-1 of Zinc finger Ran-binding domain-containing protein 2
IPI00029403	51.9	×	×	Sorting nexin-4
IPI00029485	141.7	×	×	Isoform p150 of Dynactin-1
IPI00029507	55.1	×	×	Potassium channel subfamily K member 5
IPI00029643	51.6	×	×	Isoform 2 of Mitogen-activated protein kinase kinase kinase MLT
IPI00029665	27.4	×	×	Cob
IPI00029728	86.9	×	×	TFIIH basal transcription factor complex helicase subunit
IPI00029730	34.2	×	×	Syntaxin-4
IPI00029769	57.3	×	×	Isoform p59-HCK of Tyrosine-protein kinase HCK
IPI00029822	188.1	×	×	SMARCA4 isoform 2
IPI00030045	14.3	×	×	Isoform 1 of Prokineticin-2 precursor
IPI00030104	128.1	×	×	Myosin-binding protein C, fast-type
IPI00030115	48	×	×	Bone morphogenetic protein 10 precursor
IPI00030267	180.8	×	×	Hypothetical protein DKFZp434P0316
IPI00030274	66.4	×	×	Coiled-coil domain-containing protein 55
IPI00030275	80.1	×	×	Heat shock protein 75 kDa, mitochondrial precursor
IPI00030278	55.9	×	×	Isoform 1 of TNF receptor-associated factor 2

IPI00030339	53.2	×	×	FOXD4
IPI00030363	45.2	×	×	Acetyl-CoA acetyltransferase, mitochondrial precursor
IPI00030397	31.1	×	×	Isoform 3 of Vesicle transport protein SEC20
IPI00030702	39.6	×	×	Isoform 1 of Isocitrate dehydrogenase [NAD] subunit alpha, mitochondrial precursor
IPI00030741	76.5	×	×	Protein C21orf13
IPI00030757	134.7	×	×	Isoform LpNPI of ADAMTS-2 precursor
IPI00030781	87.3	×	×	Isoform Alpha of Signal transducer and activator of transcription 1-alpha/beta
IPI00030794	79	×	×	erythrocyte protein band 4.1-like 4
IPI00030828	90.2	×	×	90 kDa protein
IPI00030876	139	×	×	Protein diaphanous homolog 1
IPI00030915	127.5	×	×	Ubiquitin carboxyl-terminal hydrolase 8
IPI00031023	144.8	×	×	Protein flightless-1 homolog
IPI00031036	84.5	×	×	Chloride anion exchanger
IPI00031055	73	×	×	hypothetical protein LOC84224
IPI00031100	63.3	×	×	Synaptotagmin-3
IPI00031386	124.4	×	×	Phosphatidylinositol-4,5-bisphosphate 3-kinase catalytic subunit alpha isoform
IPI00031388	122.8	×	×	Phosphatidylinositol-4,5-bisphosphate 3-kinase catalytic subunit beta isoform
IPI00031410	288.9	×	×	FKBP12-rapamycin complex-associated protein
IPI00031411	507	×	×	Cadherin-related tumor suppressor homolog precursor
IPI00031423	46.2	×	×	Keratin, type I cuticular Ha3-II
IPI00031425	72.7	×	×	Histidine ammonia-lyase
IPI00031456	50.1	×	×	Isoform 1 of Equilibrative nucleoside transporter 2
IPI00031490	28.7	×	×	collectin sub-family member 11 isoform a
IPI00031506	98.9	×	×	Potassium/sodium hyperpolarization-activated cyclic nucleotide-gated channel 1
IPI00031517	92.9	×	×	DNA replication licensing factor MCM6

IPI00031519	189.6	×	×	Isoform 1 of DNA (cytosine-5)-methyltransferase 1
IPI00031545	308.3	×	×	Isoform Long of Inositol 1,4,5-trisphosphate receptor type 2
IPI00031554	82.6	×	×	ATP-dependent RNA helicase DDX50
IPI00031615	15.2	×	×	Chromosome 12 open reading frame 31
IPI00031627	217.4	×	×	217 kDa protein
IPI00031670	60.7	×	×	Family with sequence similarity 114, member A1
IPI00031683	106.3	×	×	Isoform 1 of Short transient receptor potential channel 6
IPI00031773	48.3	×	×	Hepatocyte nuclear factor 3-beta
IPI00032230	120.7	×	×	Isoform A of Band 4.1-like protein 3
IPI00032316	96.9	×	×	Zinc finger protein 95 homolog
IPI00032342	225.5	×	×	TRIP12 protein
IPI00032374	82.2	×	×	Isoform 2 of Protein KIAA0179
IPI00032393	124.8	×	×	Isoform 2 of Protein diaphanous homolog 2
IPI00032491	100	×	×	Inner nuclear membrane protein Man1
IPI00032513	47.2	×	×	Keratin, type I cuticular Ha1
IPI00032541	55.8	×	×	Keratin, type II cuticular Hb5
IPI00032680	47	×	×	Apolipoprotein-L5
IPI00032905	70.6	×	×	Vacuolar protein sorting-associated protein 33B
IPI00032955	25.7	×	×	Zinc finger protein 313
IPI00032959	38.4	×	×	Glycerol-3-phosphate dehydrogenase 1-like
IPI00032970	83.6	×	×	Oxysterol-binding protein-related protein 11
IPI00032988	115	×	×	Teashirt homolog 2
IPI00033019	95.9	×	×	Potassium voltage-gated channel subfamily B member 1
IPI00033025	50.8	×	×	Septin-7
IPI00033102	52.3	×	×	Isoform 1 of Acidic mammalian chitinase precursor
IPI00033151	55.9	×	×	Polymerase kappa isoform 2
IPI00033217	102.1	×	×	Alpha-aminoadipic semialdehyde synthase, mitochondrial precursor
IPI00033322	117.4	×	×	CTCL tumor antigen se89-1
IPI00033494	19.8	×	×	Myosin regulatory light chain

IPI00033516	103.6	×	×	Isoform 1 of Gamma-tubulin complex component 3
IPI00033553	113.6	×	×	Transmembrane protein 16B
IPI00033907	216.5	×	×	Anaphase-promoting complex subunit 1
IPI00034015	52.1	×	×	Isoform 1 of CUG triplet repeat RNA-binding protein 1
IPI00036578	177.7	×	×	ADAM metallopeptidase with thrombospondin type 1 motif, 12 preproprotein
IPI00038139	136.3	×	×	similar to loss of heterozygosity, 11, chromosomal region 2, gene A homolog
IPI00043069	152.7	×	×	Ankyrin repeat domain-containing protein 30A
IPI00043302	78.2	×	×	WD repeat protein 65
IPI00043370	111.7	×	×	CDNA FLJ31910 fis, clone NT2RP7004687, weakly similar to VEGETATIBLE INCOMPATIBILITY PROTEIN HET-E-1
IPI00043810	31.4	×	×	Isoform 1 of Proline-rich transmembrane protein 1
IPI00043990	94.1	×	×	Isoform 1 of Probable ATP-dependent RNA helicase DDX31
IPI00044461	89.4	×	×	Isoform 1 of Membrane metallo-endopeptidase-like 1
IPI00044608	134.4	×	×	similar to plasma membrane associated protein, S3-12
IPI00044749	143.1	×	×	Isoform 1 of Serine/threonine-protein kinase Nek1
IPI00044751	210.6	×	×	M-phase phosphoprotein 1
IPI00044891	79.2	×	×	Adaptor molecule-1
IPI00045423	141.7	×	×	Isoform 7 of Partitioning-defective 3 homolog
IPI00045460	35.8	×	×	Hypothetical protein gs78
IPI00045478	31.3	×	×	Ras-related protein Rab-40C
IPI00045550	89.3	×	×	Neurabin-2
IPI00045914	402.2	×	×	Msx2-interacting protein
IPI00045922	81.9	×	×	Isoform 1 of Probable histone-lysine N-methyltransferase, H3 lysine-9 specific
IPI00045946	86.5	×	×	Isoform 1 of ATP-dependent metalloprotease YME1L1
IPI00046309	141.8	×	×	Diacylglycerol kinase kappa
IPI00054042	112.4	×	×	Isoform 1 of General transcription factor II-I
IPI00054598	11.3	×	×	family with sequence similarity 24, member A

IPI00056310	25.7	×	×	Secretory carrier-associated membrane protein 4
IPI00056487	41.6	×	×	F-box only protein 32
IPI00056507	63.9	×	×	Isoform 1 of F-box/WD repeat protein 5
IPI00059055	42	×	×	BarH-like 2 homeobox protein
IPI00059164	49	×	×	Galactose-3-O-sulfotransferase 3
IPI00059369	94.9	×	×	Isoform 1 of Protein KIAA0555
IPI00059930	62.8	×	×	BTB (POZ) domain containing 14A
IPI00060201	76.3	×	×	Endocrine transmitter regulatory protein
IPI00060310	55.6	×	×	phospholipase D family, member 4
IPI00060379	48.8	×	×	Integrator complex subunit 12
IPI00060544	21.7	×	×	DNA-damage-inducible transcript 4-like
IPI00060549	156.9	×	×	G-protein coupled receptor-associated sorting protein 1
IPI00061009	124.2	×	×	Isoform 1 of WD repeat protein 67
IPI00061148	115.2	×	×	sodium bicarbonate transporter 4 isoform d
IPI00061354	210.5	×	×	Isoform 2 of Bromodomain adjacent to zinc finger domain 2B
IPI00061356	70.2	×	×	Coiled-coil domain containing 99
IPI00061977	54.2	×	×	IGHA1 protein
IPI00062213	63.9	×	×	Kelch-like protein 23
IPI00062467	74.2	×	×	Isoform 1 of Zinc finger protein 274
IPI00062809	87.1	×	×	myosin head domain containing 1 isoform 1
IPI00063213	20.3	×	×	Thyroid hormone receptor-associated protein 6
IPI00063219	53.7	×	×	Hypothetical protein LOC92345
IPI00063523	277.9	×	×	similar to Temporarily Assigned Gene name family member
IPI00063679	22.1	×	×	22 kDa protein
IPI00063878	152.2	×	×	Isoform 1 of Multidrug resistance-associated protein 9
IPI00064158	142.7	×	×	Tau-tubulin kinase 1
IPI00064200	125.2	×	×	mastermind-like 2
IPI00064202	121.7	×	×	Mastermind-like protein 3
IPI00064241	64.1	×	×	Zinc finger transcription factor Eos
IPI00064323	92.5	×	×	piggyBac transposable element derived 1

IPI00064742	78.1	×	×	Similar to leishmanolysin-like
IPI00064745	62.6	×	×	Isoform 1 of Phosphatase and actin regulator 3
IPI00064885	57.7	×	×	Zinc finger protein 3 homolog
IPI00064935	201.3	×	×	Alpha-protein kinase 3
IPI00065049	55.9	×	×	CDNA FLJ25471 fis, clone TST09553
IPI00065058	33.7	×	×	spermatogenesis associated, serine-rich 1
IPI00065250	82.7	×	×	Putative adenylate kinase 7
IPI00065315	39.4	×	×	Protein C10orf27
IPI00065356	64.4	×	×	CDNA FLJ33167 fis, clone UTERU2000569
IPI00065378	80.7	×	×	Novel gene
IPI00065457	81.7	×	×	OTTHUMP00000018353
IPI00065491	41.5	×	×	Isoform 1 of SH3 and cysteine-rich domain-containing protein 3
IPI00065675	62.3	×	×	Zinc finger protein 570
IPI00066288	69.5	×	×	Isoform 1 of Pannexin-2
IPI00066458	58.1	×	×	Isoform 1 of Cyclin-L2
IPI00066817	142	×	×	Intraflagellar transport 122 homolog
IPI00069507	72.3	×	×	Isoform 2 of F-box only protein 21
IPI00069694	106.1	×	×	Isoform 1 of General transcription factor II-I repeat domain-containing protein 1
IPI00070943	231.3	×	×	Isoform 1 of Phosphatidylinositol 4-kinase alpha
IPI00071318	43.7	×	×	Isoform 1 of Putative RNA-binding protein Luc7-like 1
IPI00071509	82.9	×	×	Isoform 2 of Plakophilin-1
IPI00071824	77	×	×	Isoform 1 of Cytoskeleton-associated protein 2
IPI00072735	57.7	×	×	Isoform 1 of Cytochrome P450 3A43
IPI00073110	90.6	×	×	FYN binding protein (FYB-120/130) isoform 1
IPI00073357	44.1	×	×	Isoform 1 of F-box only protein 4
IPI00073730	101.2	×	×	Methyl-CpG-binding domain protein 6
IPI00073779	36.8	×	×	28S ribosomal protein S35, mitochondrial precursor
IPI00074258	97.3	×	×	Isoform 1 of Microtubule-associated serine/threonine-protein kinase-like

IPI00074605	74.4	×	×	Isoform 1 of Peptide-N(4)-(N-acetyl-beta-glucosaminyl)asparagine amidase
IPI00074876	115.4	×	×	Isoform 1 of PHD finger protein 20
IPI00086860	13.9	×	×	hypothetical protein LOC150383 isoform 1
IPI00088953	84.3	×	×	Phosphoinositide 3-kinase regulatory subunit 6
IPI00090327	65.1	×	×	Vacuolar protein sorting-associated protein 45
IPI00094507	29.3	×	×	UBX domain-containing protein 4
IPI00095032	56.8	×	×	57 kDa protein
IPI00095592	255.1	×	×	similar to FRAS1-related extracellular matrix protein 3 precursor
IPI00096066	46.5	×	×	Succinyl-CoA ligase [GDP-forming] beta-chain, mitochondrial precursor
IPI00097490	87.1	×	×	CDNA FLJ32660 fis, clone TESTI1000051, weakly similar to CYTADHERENCE HIGH MOLECULAR WEIGHT PROTEIN 2
IPI00097492	64.6	×	×	Zinc finger protein 512
IPI00098902	115.9	×	×	oxoglutarate (alpha-ketoglutarate) dehydrogenase (lipoamide) isoform 1 precursor
IPI00099522	133.7	×	×	Isoform 1 of Homeodomain-interacting protein kinase 3
IPI00099834	121.5	×	×	Ubinuclein
IPI00099977	39	×	×	platelet-derived growth factor C precursor
IPI00100151	108.6	×	×	Isoform 1 of 5'-3' exoribonuclease 2
IPI00100247	39	×	×	Thioredoxin domain-containing protein 13 precursor
IPI00100291	188.2	×	×	Isoform 2 of BCoR protein
IPI00100369	70.8	×	×	Ubiquilin-3
IPI00100399	62.9	×	×	Isoform 1 of THAP domain-containing protein 4
IPI00100798	222.7	×	×	CASP8-associated protein 2
IPI00100867	207.3	×	×	Transcription initiation factor TFIID 210 kDa subunit
IPI00101163	48	×	×	Isoform 1 of mTERF domain-containing protein 1, mitochondrial precursor
IPI00101186	143.7	×	×	Isoform 1 of Protein KIAA0690

IPI00101877	58.3	×	×	Phosphoinositol 4-phosphate adaptor protein-2
IPI00101923	278.9	×	×	hypothetical protein LOC80208
IPI00102107	296.7	×	×	Isoform 1 of Histone-lysine N-methyltransferase, H3 lysine-36 and H4 lysine-20 specific
IPI00102118	101.2	×	×	MLX interacting protein
IPI00102358	38.7	×	×	Neurogenic differentiation factor 6
IPI00102425	35.7	×	×	Cyclin-dependent kinase inhibitor-related protein
IPI00102575	207.6	×	×	ATP(GTP)-binding protein
IPI00102670	71.2	×	×	Isoform 2 of Formin-binding protein 1
IPI00102678	258.7	×	×	Isoform 1 of Pecanex-like protein 1
IPI00103013	65.3	×	×	SH3 and PX domain containing 3
IPI00103059	21.7	×	×	N-acetyltransferase 14
IPI00103089	24.8	×	×	MHC class I HLA-J antigen (Fragment)
IPI00103253	27	×	×	PYHIN1 protein
IPI00103415	89.9	×	×	Signal transducer and activator of transcription 5B
IPI00103430	147.6	×	×	Isoform 1 of Potassium channel subfamily T member 1
IPI00103433	56.1	×	×	TCP11b protein
IPI00103484	24.8	×	×	Isoform A of Ectodysplasin A receptor-associated adapter protein
IPI00103487	118.4	×	×	NALP7 protein
IPI00103516	126.7	×	×	Caskin-2
IPI00103525	58.7	×	×	paraspeckle protein 1 isoform alpha
IPI00103530	63.5	×	×	Atlastin
IPI00103536	183.3	×	×	MAP-kinase activating death domain-containing protein isoform d
IPI00103552	0	×	×	Mucin-16
IPI00103595	350.9	×	×	Centrosome-associated protein 350
IPI00103599	27.8	×	×	BRI3-binding protein
IPI00103655	139	×	×	Isoform Long of Autism susceptibility gene 2 protein
IPI00103685	35.1	×	×	Isoform 3 of Probable histone acetyltransferase MYST1

IPI00103762	22.6	×	×	Hypothetical protein
IPI00103869	181.1	×	×	Cortactin-binding protein 2
IPI00103980	31.8	×	×	Novel protein
IPI00103994	134.5	×	×	Leucyl-tRNA synthetase, cytoplasmic
IPI00105353	168.6	×	×	180 kDa transmembrane PLA2 receptor
IPI00105518	55.9	×	×	TEB4 protein
IPI00106552	136.2	×	×	KIAA1736 protein (Fragment)
IPI00106698	43.4	×	×	Protein pelota homolog
IPI00106847	120.6	×	×	Isoform GTBP-alt of DNA mismatch repair protein MSH6
IPI00107188	32	×	×	coiled-coil domain containing 106
IPI00107312	83	×	×	Isoform 1 of Solute carrier family 26 member 6
IPI00107625	23.3	×	×	Isoform SNAP-25a of Synaptosomal-associated protein 25
IPI00107719	74.4	×	×	bubblegum related protein
IPI00107754	98	×	×	ubiquitin protein ligase E3A isoform 1
IPI00107855	130.8	×	×	Hephaestin precursor
IPI00141561	109	×	×	Conserved oligomeric Golgi complex component 1
IPI00142538	303	×	×	Isoform 1 of Probable helicase senataxin
IPI00142768	345.2	×	×	dystonin isoform 1eB precursor
IPI00144293	84.7	×	×	Isoform 1 of YTH domain-containing protein 1
IPI00145593	96.3	×	×	Nucleolar protein with MIF4G domain 1
IPI00145799	70.1	×	×	F-box/LRR-repeat protein 4
IPI00148768	74	×	×	TRIO and F-actin binding protein isoform 1
IPI00149650	73.6	×	×	Peptidylprolyl isomerase domain and WD repeat-containing protein 1
IPI00149680	30.3	×	×	tescalcin
IPI00150554	39.4	×	×	39 kDa protein
IPI00150961	78.4	×	×	membrane component chromosome 11 surface marker 1 isoform 1
IPI00151360	65.8	×	×	Isoform 1 of GTP-binding protein 2
IPI00151710	106.2	×	×	Transmembrane protein 16F
IPI00151988	144.9	×	×	KIAA1629 protein (Fragment)

IPI00152022	90.6	×	×	Protein FAM47A
IPI00152127	15.6	×	×	Putative uncharacterized protein C11orf45 precursor
IPI00152139	63.4	×	×	Zinc finger protein 671
IPI00152310	38.3	×	×	Isoform 1 of Fibronectin type 3 and ankyrin repeat domains protein 1
IPI00152380	395.2	×	×	Myosin-15
IPI00152462	470.8	×	×	Axonemal heavy chain dynein type 3
IPI00152527	124.1	×	×	DNA helicase HEL308
IPI00152536	102.6	×	×	Isoform 1 of Transmembrane channel-like protein 2
IPI00152538	150.1	×	×	Isoform 2 of ATP-binding cassette transporter sub-family C member 11
IPI00152542	339.8	×	×	Protein DmX-like 2
IPI00152647	81.5	×	×	Synaptotagmin-like protein 5
IPI00152653	529	×	×	Ciliary dynein heavy chain 5
IPI00152661	315.4	×	×	Isoform 1 of Polycystic kidney disease 1-like 1 protein
IPI00152665	146.9	×	×	SH3 domain and tetratricopeptide repeats-containing protein 1
IPI00152705	79.3	×	×	SH3 domain containing ring finger 2
IPI00152708	58.4	×	×	U3 small nucleolar RNA-associated protein 15 homolog
IPI00152827	22.8	×	×	similar to WAS protein homology region 2 domain containing 1 isoform 1
IPI00152849	158	×	×	Isoform 1 of G2/mitotic-specific cyclin-B3
IPI00152998	68.2	×	×	Leucine-rich repeat-containing protein 40
IPI00153036	36.4	×	×	transmembrane protein 19
IPI00154283	102.2	×	×	Hypothetical protein DKFZp762J0112
IPI00154473	83.6	×	×	84 kDa protein
IPI00154515	45.8	×	×	Smad nuclear-interacting protein 1
IPI00154528	126.3	×	×	SMC6 protein
IPI00154664	109.8	×	×	Piwi-like protein 2
IPI00154668	73.2	×	×	Coiled-coil domain-containing protein 93
IPI00154755	230.6	×	×	Down syndrome cell adhesion molecule like 1

IPI00154813	78.6	×	×	Interleukin 13 receptor alpha 1-binding protein-1
IPI00154944	226.6	×	×	Isoform 1 of Sodium channel protein type 9 subunit alpha
IPI00155647	280.8	×	×	Isoform 2 of PDZ domain-containing protein 2
IPI00155729	206.8	×	×	Plexin-B3 precursor
IPI00156032	43.5	×	×	HIV-1 Rev-binding protein 2
IPI00156452	27.2	×	×	Chromosome 6 open reading frame 148
IPI00157442	167.7	×	×	Rho guanine nucleotide exchange factor 11
IPI00157757	117.9	×	×	Isoform 1 of NEDD9-interacting protein with calponin homology and LIM domains
IPI00157790	223.7	×	×	similar to Proteasome-associated protein ECM29 homolog
IPI00158296	215.1	×	×	hypothetical protein LOC23078 isoform a
IPI00158615	174.1	×	×	Isoform 1 of THO complex subunit 2
IPI00158804	56.8	×	×	basic, immunoglobulin-like variable motif containing
IPI00159899	128.5	×	×	ankyrin repeat and FYVE domain containing 1 isoform 1
IPI00160131	292.7	×	×	Myosin-IXa
IPI00160265	182.1	×	×	Isoform 2 of Trinucleotide repeat-containing gene 6A protein
IPI00160290	149.3	×	×	B-cell lymphoma 9 protein
IPI00160348	151.1	×	×	claspin
IPI00160622	281.1	×	×	Isoform 1 of Centrosome-associated protein CEP250
IPI00160716	89.4	×	×	DEP domain containing 1
IPI00161966	165.5	×	×	OTTHUMP00000022414
IPI00162547	139.4	×	×	Isoform 2 of Latrophilin-3 precursor
IPI00162563	113.7	×	×	Isoform 1 of Ubiquitin-protein ligase BRE1B
IPI00163446	63	×	×	IGHD protein
IPI00163496	136.1	×	×	136 kDa protein
IPI00163718	109.4	×	×	Isoform 4 of Vacuolar protein sorting-associated protein 54
IPI00163742	69.4	×	×	Isoform 2 of PEX5-related protein
IPI00163775	50.5	×	×	Isoform 2 of Zinc finger protein 596
IPI00163851	186.8	×	×	Isoform 1 of Eukaryotic translation initiation factor 2-alpha kinase 4

IPI00163985	101.2	×	×	Isoform 1 of Protocadherin gamma C4 precursor
IPI00164005	86.3	×	×	Conserved oligomeric Golgi complex component 7
IPI00164066	134.3	×	×	NAG6 protein
IPI00164104	195.8	×	×	Isoform 1 of Deleted in lung and esophageal cancer protein 1
IPI00164354	98.1	×	×	minichromosome maintenance protein 10 isoform 2
IPI00164861	196.1	×	×	Isoform 3 of Kinesin-like protein KIF13A
IPI00164935	76.4	×	×	Isoform 2 of TSC22 domain family protein 2
IPI00165004	120.9	×	×	Isoform 2 of Jouberin
IPI00165121	18.3	×	×	Novel protein
IPI00165138	87.8	×	×	GRIP and coiled-coil domain-containing protein 1
IPI00165189	79.7	×	×	Isoform CDV-1R of Intraflagellar transport 81
IPI00165454	122.6	×	×	WD repeat protein 60
IPI00165477	62.3	×	×	Elongation protein 3 homolog
IPI00165547	89.8	×	×	Isoform 2 of Phosphatidylinositol 4-kinase beta
IPI00165927	94.8	×	×	KIAA1608 protein (Fragment)
IPI00165934	692.7	×	×	Isoform 1 of G-protein coupled receptor 98 precursor
IPI00165981	220.2	×	×	Isoform 1 of NFX1-type zinc finger-containing protein 1
IPI00166002	58.1	×	×	ADP-ribosylation factor guanine nucleotide factor 6 isoform b
IPI00166009	132.8	×	×	Isoform 1 of JmjC domain-containing histone demethylation protein 1A
IPI00166078	62	×	×	Chromosome 1 open reading frame 87
IPI00166080	44.4	×	×	Olfactory receptor OR7-3
IPI00166085	62.3	×	×	Leucine-rich repeat LGI family member 2 precursor
IPI00166119	43.1	×	×	Actin-binding Rho-activating protein
IPI00166161	68.2	×	×	Protein SIX6OS1
IPI00166164	18.9	×	×	CDNA FLJ40719 fis, clone THYMU2028379
IPI00166165	13.5	×	×	CDNA FLJ40712 fis, clone THYMU2027249
IPI00166454	25	×	×	Hypothetical protein DKFZp666E157
IPI00166646	81.5	×	×	Isoform 1 of Junctophilin-3
IPI00166711	76.1	×	×	FLJ00369 protein (Fragment)

IPI00166892	91.2	×	×	DPPY splice variant c
IPI00166965	26.1	×	×	Putative zinc finger protein (Fragment)
IPI00166969	66.6	×	×	Solute carrier family 5 (Iodide transporter), member 8
IPI00166976	54.8	×	×	lactation elevated 1
IPI00166980	211.5	×	×	similar to CG14535-PA
IPI00166985	27.2	×	×	immunoglobulin superfamily, member 1 isoform 2
IPI00167162	31.9	×	×	CDNA FLJ25861 fis, clone CBR01776
IPI00167191	20.4	×	×	CDNA FLJ25707 fis, clone TST04879
IPI00167267	13.3	×	×	CDNA FLJ40473 fis, clone TESTI2042806
IPI00167290	37.2	×	×	CDNA FLJ40125 fis, clone TESTI2010872, weakly similar to PROTEIN PHOSPHATASE 2C BETA ISOFORM
IPI00167368	101.5	×	×	Isoform 1 of NMDA receptor-regulated 1-like protein
IPI00167447	165.6	×	×	similar to forkhead-associated (FHA) phosphopeptide binding domain 1 isoform 8
IPI00167498	142.3	×	×	Isoform 2 of Uncharacterized protein C9orf93
IPI00167619	41.2	×	×	CDNA FLJ38291 fis, clone FCBBF3009069, weakly similar to Homo sapiens HT017 mRNA
IPI00167658	21.1	×	×	Isoform 3 of Uncharacterized protein C18orf37
IPI00167663	43.8	×	×	CDNA FLJ38159 fis, clone DFNES2001404
IPI00167713	56.9	×	×	Phosphatidylinositol-glycan biosynthesis class W protein
IPI00167763	43.9	×	×	CDNA FLJ36879 fis, clone BGGI11000193
IPI00167808	69.7	×	×	CDNA FLJ36564 fis, clone TRACH2009851
IPI00167829	79.1	×	×	CDNA FLJ36144 fis, clone TESTI2025022, weakly similar to TRICHOHYALIN
IPI00167850	53.4	×	×	Isoform 1 of Zinc finger protein 610
IPI00167953	69.8	×	×	BTB and kelch domain containing 3
IPI00168056	134.3	×	×	Zinc finger and BTB domain-containing protein 38
IPI00168111	13.1	×	×	CDNA FLJ34302 fis, clone FEBRA2007620
IPI00168280	117.8	×	×	Isoform 1 of NACHT, LRR and PYD-containing protein 11
IPI00168340	63.8	×	×	Hypothetical protein FLJ90709

IPI00168347	89.7	×	×	IMP dehydrogenase/GMP reductase family protein
IPI00168459	136.9	×	×	Isoform 2 of Pleckstrin homology-like domain family B member 2
IPI00168529	109.1	×	×	hypothetical protein LOC126859 isoform 2
IPI00168560	58.4	×	×	Zinc finger protein 439
IPI00168607	151	×	×	TRP domain containing protein
IPI00168608	100.6	×	×	Hypothetical protein LOC222967
IPI00168611	65	×	×	Vesicular glutamate transporter 3
IPI00168663	53.6	×	×	54 kDa protein
IPI00168698	128.6	×	×	PDZ domain-containing protein 8
IPI00168723	12.8	×	×	FLJ00387 protein (Fragment)
IPI00168728	56.1	×	×	FLJ00385 protein (Fragment)
IPI00168806	541.3	×	×	Isoform 1 of Myeloid/lymphoid or mixed-lineage leukemia protein 3 homolog
IPI00168819	84.6	×	×	Ankyrin repeat and protein kinase domain-containing protein 1
IPI00168877	123.3	×	×	helicase (DNA) B
IPI00168878	51.3	×	×	Torsin-1A-interacting protein 2
IPI00168885	155.6	×	×	Isoform 1 of Putative ATP-dependent RNA helicase DHX57
IPI00168913	139.9	×	×	Isoform 2 of Limbin
IPI00168920	175.5	×	×	collagen, type XXIV, alpha 1
IPI00168964	40.9	×	×	Seven transmembrane helix receptor
IPI00168974	35.5	×	×	Olfactory receptor 4E2
IPI00169113	35.6	×	×	Olfactory receptor 13C4
IPI00169261	58	×	×	Tubulin--tyrosine ligase-like protein 11
IPI00169325	105.3	×	×	WD repeat protein 36
IPI00169345	72	×	×	Protein NEDD1
IPI00169377	96.5	×	×	Isoform 1 of GAS2-like protein 2
IPI00169426	61.9	×	×	Isoform 2 of Cytosolic 5'-nucleotidase 1B
IPI00170429	63.2	×	×	2-oxoglutarate and iron-dependent oxygenase domain containing 1
IPI00170551	111.7	×	×	Isoform 2 of Semaphorin-6D precursor

IPI00170597	74.9	×	×	Isoform 1 of Protein TSPEAR precursor
IPI00170800	128.5	×	×	Isoform 1 of Otoancorin precursor
IPI00171134	216	×	×	Girdin
IPI00171230	114.1	×	×	Isoform 2 of ELKS/RAB6-interacting/CAST family member 1
IPI00171274	129.4	×	×	Isoform C of Myotubularin-related protein 3
IPI00171444	62.3	×	×	G protein-coupled receptor 177 isoform 1
IPI00171475	45.8	×	×	nuclear factor of activated T-cells, cytoplasmic, calcineurin-dependent 2 interacting protein
IPI00171480	70	×	×	IMP dehydrogenase/GMP reductase family protein
IPI00171491	111.9	×	×	Isoform 1 of Potassium voltage-gated channel subfamily H member 5
IPI00171494	493.4	×	×	similar to dynein, cytoplasmic, heavy chain 2 isoform 1
IPI00171516	50.6	×	×	Plasmalemma vesicle-associated protein
IPI00171636	202.5	×	×	Neuron navigator 1
IPI00171678	69.1	×	×	dopamine beta-hydroxylase precursor
IPI00171925	78.9	×	×	Pentatricopeptide repeat protein 1
IPI00171928	40	×	×	Angiopoietin-related protein 7 precursor
IPI00172452	56.9	×	×	calcium/calmodulin-dependent protein kinase II gamma isoform 6
IPI00173347	179.6	×	×	NEDD4-like ubiquitin-protein ligase 1
IPI00173549	136.3	×	×	Hypothetical protein SYNPO2 (Fragment)
IPI00174025	177.3	×	×	Isoform 1 of Dynamin-binding protein
IPI00174345	251.7	×	×	PF6
IPI00174391	131.9	×	×	similar to chromosome 9 open reading frame 36
IPI00174574	103.4	×	×	hypothetical protein LOC159686
IPI00174756	66.4	×	×	Ankyrin repeat domain-containing protein 21
IPI00174775	58.9	×	×	Keratin 6 irs3
IPI00174853	59.1	×	×	59 kDa protein
IPI00174902	70.3	×	×	similar to Zinc finger protein ZFP-36
IPI00175212	23	×	×	similar to ribosomal protein L19 isoform 3
IPI00175313	94.3	×	×	Novel protein

IPI00175416	190.2	×	×	Phospholipase C-eta1b
IPI00175420	43	×	×	HPMSR2
IPI00176454	17.3	×	×	Protein SSX8
IPI00176466	79	×	×	hypothetical protein
IPI00176482	131.2	×	×	CDNA FLJ42404 fis, clone ASTRO3000301, highly similar to Human G2 protein
IPI00177437	100.6	×	×	Isoform 1 of Crooked neck-like protein 1
IPI00177476	69	×	×	aarF domain containing kinase 2
IPI00177743	251.1	×	×	251 kDa protein
IPI00178014	13.6	×	×	CDNA FLJ11122 fis, clone PLACE1006159
IPI00178072	124.6	×	×	Cytospin-A
IPI00178185	93.5	×	×	Isoform 1 of Cytoskeleton-like bicaudal D protein homolog 2
IPI00178187	140	×	×	Hypothetical protein DKFZp566N1646
IPI00178675	240.3	×	×	240 kDa protein
IPI00178743	461.2	×	×	ALMS1
IPI00179071	219.1	×	×	Nuclear receptor coactivator 6
IPI00179109	43.2	×	×	Isoform 1 of NAD-dependent deacetylase sirtuin-2
IPI00179169	146.7	×	×	Isoform 1 of X-linked retinitis pigmentosa GTPase regulator-interacting protein 1
IPI00179337	89.3	×	×	Isoform 1 of B-cell scaffold protein with ankyrin repeats
IPI00179357	3734.8	×	×	Isoform 7 of Titin
IPI00180161	99.5	×	×	99 kDa protein
IPI00180305	185.4	×	×	Retinoblastoma-associated factor 600
IPI00180325	100.3	×	×	CDNA FLJ37794 fis, clone BRHIP3000488
IPI00180384	461.1	×	×	Ciliary dynein heavy chain 7
IPI00180404	80.7	×	×	Nexilin
IPI00180408	224.6	×	×	similar to Myosin heavy chain, cardiac muscle alpha isoform
IPI00180559	61.9	×	×	Isoform 2 of Neuroepithelial cell-transforming gene 1 protein
IPI00180615	18.6	×	×	Similar to E2F transcription factor 7
IPI00180764	70.6	×	×	Histone acetyltransferase MYST2

IPI00180922	80.1	×	×	Isoform 2 of Protein FAM48A
IPI00180926	56.1	×	×	NHE domain-containing 1 protein
IPI00181116	33.8	×	×	U3 small nucleolar ribonucleoprotein protein IMP4
IPI00181231	78.6	×	×	79 kDa protein
IPI00181285	60.5	×	×	Testis-specific chromodomain protein Y 2
IPI00181376	70.1	×	×	Isoform 1 of Tumor-associated hydroquinone oxidase
IPI00181641	204.9	×	×	Isoform 2 of YLP motif-containing protein 1
IPI00181741	25.9	×	×	protein tyrosine phosphatase, non-receptor type 20A isoform 2
IPI00181743	131.9	×	×	Isoform 1 of BAI1-associated protein 3
IPI00182048	42.5	×	×	Sphingosine kinase 1
IPI00182194	307.7	×	×	Teneurin-2
IPI00182560	62	×	×	Cytochrome P450, family 2, subfamily E, polypeptide 2 homolog
IPI00182655	53.7	×	×	Keratin, type II cuticular Hb6
IPI00182739	111.4	×	×	junction-mediating and regulatory protein
IPI00182757	103	×	×	Isoform 2 of Protein KIAA1967
IPI00182833	88.7	×	×	Coiled-coil alpha-helical rod protein 1
IPI00183015	91.1	×	×	Isoform 2 of Mucosa-associated lymphoid tissue lymphoma translocation protein 1
IPI00183054	110.1	×	×	E3 ubiquitin-protein ligase MIB1
IPI00183706	93.2	×	×	KIF19 protein (Fragment)
IPI00183938	96.8	×	×	CDNA FLJ14664 fis, clone NT2RP2002880
IPI00184090	61.2	×	×	CDNA FLJ34563 fis, clone KIDNE2002798
IPI00184330	101.9	×	×	DNA replication licensing factor MCM2
IPI00184851	38.2	×	×	Type 2 lactosamine alpha-2,3-sialyltransferase
IPI00184876	117.5	×	×	solute carrier family 4, sodium bicarbonate cotransporter, member 8 isoform b
IPI00185027	172.4	×	×	Isoform 1 of Arginine-glutamic acid dipeptide repeats protein
IPI00185037	89	×	×	Isoform 1 of Serine/threonine-protein kinase MARK1
IPI00185191	41.7	×	×	42 kDa protein
IPI00185459	51.3	×	×	51 kDa protein

IPI00185519	108.6	×	×	MYCBP associated protein
IPI00185769	108.9	×	×	Isoform Beta of ATP-dependent DNA helicase Q5
IPI00185919	123.5	×	×	Isoform 1 of La-related protein 1
IPI00186525	53.7	×	×	Isoform 2 of IQ calmodulin-binding motif-containing protein 1
IPI00186690	52.6	×	×	Isoform 2 of ARF GTPase-activating protein GIT2
IPI00186946	65.5	×	×	Isoform 1 of Collagen alpha-1(XXV) chain
IPI00186966	64.7	×	×	Isoform IIA of Myc box-dependent-interacting protein 1
IPI00187002	137.9	×	×	Novel protein
IPI00187091	47.4	×	×	Isoform 2 of MAP kinase-interacting serine/threonine-protein kinase 1
IPI00187146	68	×	×	Hypothetical protein DKFZp667K087
IPI00215722	145.6	×	×	Isoform JM-B of Receptor tyrosine-protein kinase erbB-4 precursor
IPI00215724	68.5	×	×	Isoform 4 of Fragile X mental retardation 1 protein
IPI00215763	42.2	×	×	Isoform 2 of MAP kinase-activated protein kinase 2
IPI00215869	54.2	×	×	Progressive ankylosis protein homolog
IPI00215873	66.3	×	×	Isoform 3 of Dual specificity tyrosine-phosphorylation-regulated kinase 1B
IPI00215876	22.9	×	×	CDNA FLJ46732 fis, clone TRACH3019807, weakly similar to Antigen LY- 9
IPI00215890	25.9	×	×	Zinc finger protein 22
IPI00215943	518	×	×	Isoform 3 of Plectin-1
IPI00215948	100.1	×	×	Isoform 1 of Catenin alpha-1
IPI00215963	136.2	×	×	Isoform 1 of Genetic suppressor element 1
IPI00215999	92.7	×	×	Isoform SV9 of PITSLRE serine/threonine-protein kinase CDC2L1
IPI00216043	77.5	×	×	Isoform sFlt1 of Vascular endothelial growth factor receptor 1 precursor
IPI00216046	122.6	×	×	Isoform 1 of Probable global transcription activator SNF2L1
IPI00216363	85.1	×	×	Isoform 2 of Granulocyte colony-stimulating factor receptor precursor

IPI00216412	83.1	×	×	Isoform 2 of Coiled coil-containing protein C6orf97
IPI00216651	57.7	×	×	Isoform 1 of Interleukin-28 receptor alpha chain precursor
IPI00216750	53.5	×	×	Isoform 1 of Developmentally-regulated RNA-binding protein 1
IPI00216850	37.6	×	×	Hypothetical protein MGC34713
IPI00216882	43.6	×	×	mannan-binding lectin serine protease 1 isoform 3
IPI00216915	40.2	×	×	GRAM domain containing 2
IPI00216990	138.5	×	×	KIAA2026
IPI00217010	57.9	×	×	Acetylcholine receptor protein subunit gamma precursor
IPI00217018	184.3	×	×	Isoform 1 of Sterile alpha motif domain-containing protein 9
IPI00217051	255.6	×	×	Steerin3 protein
IPI00217052	268.2	×	×	neuron navigator 2 isoform 1
IPI00217108	57	×	×	Uncharacterized protein C2orf13
IPI00217116	48.1	×	×	Similar to tumor necrosis factor receptor superfamily, member 1B (Fragment)
IPI00217146	94.3	×	×	SLIT and NTRK-like protein 4 precursor
IPI00217225	161.2	×	×	Isoform 1 of Nitric-oxide synthase, brain
IPI00217283	123.2	×	×	similar to pleckstrin homology domain containing, family M (with RUN domain) member 1
IPI00217287	118.4	×	×	Isoform 2 of Protein AL017
IPI00217355	110.5	×	×	Isoform 1 of Zinc finger CCCH-type domain-containing protein 7A
IPI00217393	124.7	×	×	Isoform 1 of Centaurin-gamma 1
IPI00217405	200.2	×	×	Isoform 1 of E3 ubiquitin-protein ligase UBR1
IPI00217412	529	×	×	AGO2
IPI00217413	155.2	×	×	Putative ATP-dependent RNA helicase DHX29
IPI00217437	184.6	×	×	Tau-tubulin kinase
IPI00217438	53.1	×	×	Isoform 4 of Zinc finger protein 690
IPI00217439	134	×	×	Isoform 1 of Codanin-1
IPI00217465	21.2	×	×	Histone H1.2

IPI00217499	281	×	×	Isoform BI-1(V2) of Voltage-dependent P/Q-type calcium channel subunit alpha-1A
IPI00217511	56.4	×	×	Seven transmembrane helix receptor
IPI00217513	100.8	×	×	Isoform 1 of Neuronal PAS domain-containing protein 3
IPI00217600	146.2	×	×	neuropathy target esterase
IPI00217605	57.4	×	×	Protein C6orf146
IPI00217620	111.5	×	×	Hypothetical protein FLJ25416
IPI00217687	103.5	×	×	Chromosome 16 open reading frame 50
IPI00217688	27.6	×	×	Chromosome 12 open reading frame 60
IPI00217691	110.1	×	×	Chromosome 17 open reading frame 57
IPI00217710	165.8	×	×	Isoform C of Nuclear factor of activated T-cells 5
IPI00217735	59.2	×	×	Isoform BMAL1F of Aryl hydrocarbon receptor nuclear translocator-like protein 1
IPI00217756	41.8	×	×	Isoform 1 of Uncharacterized protein C18orf54 precursor
IPI00217792	51.2	×	×	WD repeat domain 40B
IPI00217802	103.8	×	×	Uncharacterized protein C12orf25
IPI00217809	62.8	×	×	Zinc finger protein 100
IPI00217810	13.6	×	×	Transcription elongation factor A protein-like 8
IPI00217865	74.9	×	×	Isoform 1 of FYVE, RhoGEF and PH domain-containing protein 2
IPI00217872	63.8	×	×	Phosphoglucomutase 1
IPI00217911	58.7	×	×	CDNA FLJ42455 fis, clone BRACE2015314
IPI00217923	86.8	×	×	DAZ interacting protein 1-like
IPI00217957	256.3	×	×	Isoform 2 of AT-hook-containing transcription factor 1
IPI00217963	51.1	×	×	Keratin, type I cytoskeletal 16
IPI00217972	186.9	×	×	Collagen XXVII proalpha 1 chain precursor
IPI00217976	80.9	×	×	Microtubule-associated protein tau
IPI00217985	233.1	×	×	Dedicator of cytokinesis protein 3
IPI00218000	116.3	×	×	Isoform 1 of Probable G-protein coupled receptor 113 precursor
IPI00218013	144.2	×	×	Isoform 2 of Shugoshin-like 2
IPI00218052	395.3	×	×	Isoform 1 of WD repeat and FYVE domain-containing protein 3

IPI00218081	140.3	×	×	hypothetical protein LOC92126
IPI00218087	124.5	×	×	Isoform 1 of SLIT-ROBO Rho GTPase-activating protein 3
IPI00218097	145.4	×	×	Isoform 2 of C-jun-amino-terminal kinase-interacting protein 4
IPI00218130	97	×	×	Glycogen phosphorylase, muscle form
IPI00218132	57	×	×	F-box-like/WD repeat protein TBL1X
IPI00218136	137.4	×	×	137 kDa protein
IPI00218189	63.5	×	×	Zinc finger protein 14 homolog
IPI00218229	38.2	×	×	Isoform B of LIM/homeobox protein Lhx6.1
IPI00218236	37.1	×	×	Serine/threonine-protein phosphatase PP1-beta catalytic subunit
IPI00218497	145.8	×	×	Isoform 1 of Misshapen-like kinase 1
IPI00218521	221.5	×	×	Isoform 2 of Sodium channel protein type 3 subunit alpha
IPI00218539	182.5	×	×	Isoform B of Collagen alpha-1(XI) chain precursor
IPI00218563	77.9	×	×	Isoform 2 of SH3 adapter protein SPIN90
IPI00218618	250.4	×	×	Isoform B of SON protein
IPI00218638	124.8	×	×	Myosin If
IPI00218718	80.6	×	×	acyl-CoA synthetase long-chain family member 6 isoform b
IPI00218725	343.9	×	×	laminin alpha 2 subunit precursor
IPI00218767	52.1	×	×	Isoform 2 of Glucokinase
IPI00218795	43.6	×	×	L-selectin precursor
IPI00218823	293.7	×	×	Isoform 1 of Myeloid/lymphoid or mixed-lineage leukemia protein 4
IPI00218845	133.2	×	×	Nitric-oxide synthase, endothelial
IPI00218922	87.9	×	×	Translocation protein SEC63 homolog
IPI00218925	24.1	×	×	Peroxisomal membrane protein 4
IPI00218982	207.7	×	×	Breast cancer type 1 susceptibility protein
IPI00218987	126.9	×	×	Integrin alpha-D precursor
IPI00219078	114.8	×	×	Isoform SERCA2B of Sarcoplasmic/endoplasmic reticulum calcium ATPase 2
IPI00219103	22.3	×	×	Neuron-specific calcium-binding protein hippocalcin

IPI00219168	417.2	×	×	Spectrin beta chain, brain 4
IPI00219173	94.1	×	×	Isoform 1 of Exonuclease 1
IPI00219179	104.9	×	×	105 kDa protein
IPI00219257	127.1	×	×	Isoform ZA of Plasma membrane calcium-transporting ATPase 3
IPI00219299	271.8	×	×	272 kDa protein
IPI00219358	46.7	×	×	mannose-6- phosphate isomerase
IPI00219417	121.3	×	×	Isoform 1 of Tyrosine-protein kinase JAK3
IPI00219420	141.5	×	×	Structural maintenance of chromosome 3
IPI00219477	28.2	×	×	Isoform 2 of Beta-1,3-N-acetylglucosaminyltransferase lunatic fringe
IPI00219561	124.7	×	×	NACHT, LRR and PYD-containing protein 14
IPI00219568	44.7	×	×	Phosphoglycerate kinase, testis specific
IPI00219601	88.4	×	×	mitogen-activated protein kinase 7 isoform 1
IPI00219613	117.5	×	×	Isoform 1 of Presequence protease, mitochondrial precursor
IPI00219696	177	×	×	ATP-binding cassette, sub-family C, member 8
IPI00219740	44.6	×	×	Isoform 2 of DNA replication licensing factor MCM7
IPI00219774	45.4	×	×	cAMP-dependent protein kinase type II-alpha regulatory subunit
IPI00219798	180.9	×	×	Isoform 1 of Roundabout homolog 1 precursor
IPI00219983	57.9	×	×	Isoform 2 of Voltage-dependent L-type calcium channel subunit beta-1
IPI00220109	278.2	×	×	Isoform 3 of Transcriptional regulator ATRX
IPI00220113	102.9	×	×	Isoform 2 of Microtubule-associated protein 4
IPI00220160	52.4	×	×	52 kDa protein
IPI00220259	27.5	×	×	CDNA FLJ13947 fis, clone Y79AA1000985, highly similar to Human centrosomal protein kendrin mRNA
IPI00220266	33.1	×	×	Isoform 2 of Asialoglycoprotein receptor 2
IPI00220317	165.9	×	×	DNA polymerase alpha catalytic subunit
IPI00220391	565.4	×	×	565 kDa protein
IPI00220477	53	×	×	Isoform 2 of Microspherule protein 1
IPI00220490	134.9	×	×	SH3 and multiple ankyrin repeat domains 2 isoform 1

IPI00220621	82.1	×	×	Isoform PDE4B3 of cAMP-specific 3',5'-cyclic phosphodiesterase 4B
IPI00220637	58.6	×	×	Seryl-tRNA synthetase, cytoplasmic
IPI00220644	58.1	×	×	pyruvate kinase 3 isoform 2
IPI00220737	83.8	×	×	Isoform N-CAM 120 of Neural cell adhesion molecule 1, 120 kDa isoform precursor
IPI00220741	281.2	×	×	Spectrin alpha chain, erythrocyte
IPI00220813	53.7	×	×	Isoform 2 of EGF-containing fibulin-like extracellular matrix protein 1 precursor
IPI00220901	146.7	×	×	TBC1 domain family member 4
IPI00221040	136.7	×	×	137 kDa protein
IPI00221091	14.7	×	×	40S ribosomal protein S15a
IPI00221106	100.2	×	×	splicing factor 3B subunit 2
IPI00221113	89.3	×	×	Isoform 2 of Protocadherin alpha 5 precursor
IPI00221118	54.5	×	×	Isoform Long of NADPH:adrenodoxin oxidoreductase, mitochondrial precursor
IPI00221211	48	×	×	Isoform 3 of Transmembrane protease, serine 4
IPI00221325	358.2	×	×	Ran-binding protein 2
IPI00232262	51.5	×	×	similar to tripartite motif protein 17
IPI00232492	63.8	×	×	Isoform Beta of Tripartite motif-containing protein 29
IPI00232812	8	×	×	8 kDa protein
IPI00232818	69.5	×	×	similar to WD repeat domain 42B
IPI00234154	85.7	×	×	ankyrin repeat domain 56
IPI00234252	122.8	×	×	SWI/SNF-related matrix-associated actin-dependent regulator of chromatin subfamily C member 1
IPI00234337	141.7	×	×	similar to Probable cation-transporting ATPase 13A3 (ATPase family homolog up-regulated in senescence cells 1) isoform 3
IPI00234446	54.5	×	×	activating transcription factor 2
IPI00235481	119.1	×	×	PMFBP1 protein
IPI00236901	106.7	×	×	Isoform 1 of Ubiquitin carboxyl-terminal hydrolase 33

IPI00240812	164.7	×	×	Hypothetical protein
IPI00241562	388.2	×	×	Isoform 2 of Reelin precursor
IPI00241841	57.8	×	×	keratin 6L
IPI00242962	174.9	×	×	Isoform 1 of Protein AL017
IPI00243451	76.3	×	×	Liver-specific organic anion transporter 3TM12
IPI00244391	146.3	×	×	Xanthine dehydrogenase/oxidase
IPI00247110	106.4	×	×	similar to ankyrin repeat domain 26 isoform 1
IPI00247295	1005.2	×	×	Isoform 4 of Nesprin-1
IPI00247309	99.8	×	×	Isoform B of Nuclear factor of activated T-cells, cytoplasmic 2
IPI00247439	139	×	×	Isoform 2 of STE20-like serine/threonine-protein kinase
IPI00248384	21.1	×	×	CDNA FLJ36947 fis, clone BRACE2005681
IPI00248639	36.9	×	×	37 kDa protein
IPI00248651	352.8	×	×	DNA polymerase zeta catalytic subunit
IPI00248911	40.4	×	×	similar to Guanine nucleotide-binding protein G(t), alpha-3 subunit
IPI00251344	351.2	×	×	351 kDa protein
IPI00251559	114	×	×	114 kDa protein
IPI00256611	65.7	×	×	CDNA FLJ35445 fis, clone SMINT2003551, weakly similar to Drosophila melanogaster La related protein (larp) mRNA (Fragment)
IPI00256638	40.6	×	×	CDNA FLJ90286 fis, clone NT2RP1000740
IPI00256861	620.4	×	×	Isoform 2 of Microtubule-actin cross-linking factor 1, isoforms 1/2/3/5
IPI00257216	22.5	×	×	similar to Ssu72 RNA polymerase II CTD phosphatase homolog
IPI00257731	39.6	×	×	Isoform 2 of Tumor suppressor candidate 3
IPI00257910	76.3	×	×	Novel protein similar to multidomain presynaptic cytomatrix protein Piccolo; piccolo
IPI00260715	53.4	×	×	Fus-like protein (Fragment)
IPI00261031	145.6	×	×	similar to hephaestin isoform 1

IPI00288958	154	×	×	CDNA FLJ13755 fis, clone PLACE3000363
IPI00288964	136	×	×	Isoform 1 of Transmembrane protein 16H
IPI00288965	264	×	×	Proto-oncogene tyrosine-protein kinase ROS precursor
IPI00288988	126.6	×	×	Novel protein
IPI00289029	94.8	×	×	KIAA1602 protein (Fragment)
IPI00289034	184.8	×	×	Isoform 1 of Histone-lysine N-methyltransferase, H3 lysine-79 specific
IPI00289131	84.7	×	×	Transmembrane protein 8 precursor
IPI00289258	237.4	×	×	Myosin-10
IPI00289301	81.5	×	×	Nucleoporin NYD-SP7
IPI00289342	108.3	×	×	Ephrin type-B receptor 4 precursor
IPI00289344	270.2	×	×	Isoform 1 of Nuclear receptor corepressor 1
IPI00289435	61.7	×	×	Isoform 1 of Putative Rho-GTPase-activating protein FLJ46335 precursor
IPI00289462	70.1	×	×	Isoform 5 of Molybdenum cofactor biosynthesis protein 1 B
IPI00289608	125.4	×	×	Isoform 2 of AP-3 complex subunit delta-1
IPI00289649	104	×	×	Tyrosine-protein phosphatase non-receptor type 3
IPI00289746	61.6	×	×	Isoform 2 of Serine/threonine-protein kinase PAK 1
IPI00289776	510.2	×	×	Isoform 1 of Probable E3 ubiquitin-protein ligase MYCBP2
IPI00289795	51.6	×	×	Isoform 1 of Sushi repeat-containing protein SRPX precursor
IPI00289801	55.1	×	×	CDNA FLJ43079 fis, clone BRTHA3016917, weakly similar to Valyl-tRNA synthetase 2
IPI00289849	89.7	×	×	Leucine-rich repeat and fibronectin type-III domain-containing protein 6 precursor
IPI00290035	216.1	×	×	Protocadherin-15 precursor
IPI00290077	49.2	×	×	Keratin, type I cytoskeletal 15
IPI00290078	63.9	×	×	keratin 4
IPI00290094	104.8	×	×	Splicing factor, arginine/serine-rich 8
IPI00290116	57.6	×	×	Cytochrome P450 11B2, mitochondrial precursor
IPI00290189	59.1	×	×	Bardet-Biedl syndrome 4 splice variant 1

IPI00290283	81.9	×	×	mannan-binding lectin serine protease 1 isoform 2 precursor
IPI00290350	121.1	×	×	Isoform 2 of Protocadherin-19 precursor
IPI00290368	74.1	×	×	Histidine decarboxylase
IPI00290410	90.7	×	×	Acidic 82 kDa protein mRNA
IPI00290543	68.1	×	×	nuclear protein localization 4
IPI00290571	82.3	×	×	F-box only protein 30
IPI00290652	163.8	×	×	remodeling and spacing factor 1
IPI00290770	60.5	×	×	chaperonin containing TCP1, subunit 3 isoform b
IPI00290799	30.8	×	×	hypothetical protein LOC125228
IPI00290812	129.6	×	×	Isoform 1 of Transcription termination factor 2
IPI00290824	86.4	×	×	TBC1 domain family member 16
IPI00290834	52.8	×	×	Isoform 1 of Calcium-binding tyrosine phosphorylation-regulated protein
IPI00290933	98.6	×	×	Isoform 1 of Piwi-like protein 1
IPI00290950	62.9	×	×	WD repeat protein 20
IPI00291003	111.1	×	×	Hypothetical protein MGC4562
IPI00291200	129	×	×	Nuclear pore complex protein Nup133
IPI00291205	125	×	×	cGMP-inhibited 3',5'-cyclic phosphodiesterase A
IPI00291215	184.3	×	×	Isoform 2 of Poly [ADP-ribose] polymerase 14
IPI00291235	51.9	×	×	Beclin-1
IPI00291278	50.5	×	×	Interferon-induced protein 44
IPI00291347	282.6	×	×	Alpha1A-voltage-dependent calcium channel
IPI00291351	118.7	×	×	Ribosomal protein S6 kinase delta-1
IPI00291364	89.3	×	×	TFIIH basal transcription factor complex helicase XPB subunit
IPI00291392	65.1	×	×	Isoform 2 of Taste receptor type 1 member 1 precursor
IPI00291540	50.3	×	×	Keratin, type I cuticular Ha2
IPI00291579	110.1	×	×	Isoform 1 of Kinesin-like protein KIF23
IPI00291624	30.6	×	×	OTTHUMP00000016846
IPI00291755	205.5	×	×	206 kDa protein
IPI00291807	207.9	×	×	208 kDa protein

IPI00291827	104.3	×	×	Neutral alpha-glucosidase C
IPI00291930	68.3	×	×	Isoform 1 of Clathrin interactor 1
IPI00291939	143.2	×	×	Structural maintenance of chromosome 1-like 1 protein
IPI00292043	239.4	×	×	Isoform 1 of Protein sidekick-2 precursor
IPI00292071	53	×	×	Secretogranin-3 precursor
IPI00292135	70.7	×	×	Lamin-B receptor
IPI00292376	106.7	×	×	GEM-interacting protein
IPI00292380	97.3	×	×	Isoform 1 of Phosphoinositide 3-kinase regulatory subunit 5
IPI00292445	75.7	×	×	CpG-binding protein
IPI00292471	193.4	×	×	Centaurin-delta 1
IPI00292548	77	×	×	Rhophilin-2
IPI00292615	50.1	×	×	zinc finger and BTB domain containing 8
IPI00292660	57.4	×	×	Zinc finger protein 655
IPI00292715	49.4	×	×	keratin 34
IPI00292748	96.6	×	×	Isoform 1 of Whirlin
IPI00292791	112.8	×	×	Contactin-3 precursor
IPI00292836	191.1	×	×	OTTHUMP00000021741
IPI00292914	151.3	×	×	similar to Ankyrin repeat and IBR domain-containing protein 1
IPI00292934	120.8	×	×	Inactive ubiquitin carboxyl-terminal hydrolase 53
IPI00292953	110.4	×	×	Isoform 2 of Ankycorbin
IPI00293251	591	×	×	Isoform 6 of Bullous pemphigoid antigen 1, isoforms 6/9/10
IPI00293338	128.8	×	×	SMC5 structural maintenance of chromosomes 5-like 1
IPI00293396	91.7	×	×	adaptor-related protein complex 1, gamma 1 subunit isoform a
IPI00293565	152.8	×	×	fms-related tyrosine kinase 4 isoform 1
IPI00293592	55.7	×	×	hypothetical protein LOC146562
IPI00293655	82.4	×	×	ATP-dependent RNA helicase DDX1
IPI00293665	59.9	×	×	Keratin, type II cytoskeletal 6B
IPI00293826	62.2	×	×	TBC1 domain family member 3
IPI00293963	66.4	×	×	Chromodomain Y-like protein
IPI00294084	96.5	×	×	Endothelial PAS domain-containing protein 1

IPI00294148	278.8	×	×	hypothetical protein LOC23379
IPI00294173	156.3	×	×	Ninein-like protein
IPI00294187	75.6	×	×	Protein-arginine deiminase type-2
IPI00294212	46.3	×	×	similar to Tetratricopeptide repeat protein 9
IPI00294344	93.5	×	×	Proto-oncogene tyrosine-protein kinase Fes/Fps
IPI00294402	82.3	×	×	Isoform 1 of Origin recognition complex subunit 3
IPI00294556	92.9	×	×	Amyloid beta A4 precursor protein-binding family A member 1
IPI00294640	91.9	×	×	Isoform 1 of Collagen alpha-1(IX) chain precursor
IPI00294649	50.4	×	×	keratin 35
IPI00294653	258.2	×	×	Protein C21orf5
IPI00294728	337.9	×	×	Protein DmX-like 1
IPI00294749	160.3	×	×	KIF27A
IPI00294787	150.8	×	×	RAD54-like 2
IPI00294810	101.9	×	×	CDNA FLJ10824 fis, clone NT2RP4001086
IPI00294834	86	×	×	86 kDa protein
IPI00294840	188.7	×	×	Absent in melanoma 1 protein
IPI00294891	94.1	×	×	Proliferating-cell nucleolar antigen p120
IPI00294978	86.5	×	×	86 kDa protein
IPI00294982	66.2	×	×	Isoform Long of Estrogen receptor
IPI00294997	42.5	×	×	Nucleoside diphosphate kinase 7
IPI00294999	79	×	×	CDNA FLJ39238 fis, clone OCBBF2007946
IPI00295022	165.7	×	×	NK-tumor recognition protein
IPI00295105	35.4	×	×	Carbonic anhydrase 6 precursor
IPI00295133	95.3	×	×	Isoform CD22-beta of B-cell receptor CD22 precursor
IPI00295209	46.8	×	×	Sorting nexin-5
IPI00295252	186.2	×	×	Isoform 1 of Phosphatidylinositol 3,4,5-trisphosphate-dependent Rac exchanger 1 protein
IPI00295339	90.8	×	×	P-selectin precursor
IPI00295380	47.5	×	×	CDNA FLJ11142 fis, clone PLACE1006552
IPI00295461	87.8	×	×	Isoform 1 of Seprase

IPI00295502	170.2	×	×	Homolog of Mus musculus wizL protein
IPI00295577	224.3	×	×	Receptor-type tyrosine-protein phosphatase beta precursor
IPI00295701	80.7	×	×	Isoform 1 of Amyotrophic lateral sclerosis 2 chromosomal region candidate gene 8 protein
IPI00295716	38.4	×	×	Novel protein
IPI00295771	60.2	×	×	Cytochrome P450 11A1, mitochondrial precursor
IPI00295832	49.6	×	×	Oligodendrocyte-myelin glycoprotein precursor
IPI00295851	107.1	×	×	Coatomer subunit beta
IPI00295865	38.6	×	×	SRR1-like protein
IPI00295976	113.4	×	×	Isoform 1 of Integrin alpha-IIb precursor
IPI00296036	72.6	×	×	Eomesodermin homolog
IPI00296078	143	×	×	zinc finger transcription factor TRPS1
IPI00296147	29.2	×	×	Transmembrane protein 119 precursor
IPI00296183	50.4	×	×	Aldehyde dehydrogenase, dimeric NADP-preferring
IPI00296196	96.8	×	×	Dimethylglycine dehydrogenase, mitochondrial precursor
IPI00296199	42.2	×	×	translocation associated membrane protein 1-like 1
IPI00296291	61.2	×	×	HP1-BP74
IPI00296318	94.2	×	×	E2F family member 8
IPI00296337	469.1	×	×	Isoform 1 of DNA-dependent protein kinase catalytic subunit
IPI00296362	409.8	×	×	Isoform 1 of Abnormal spindle-like microcephaly-associated protein
IPI00296365	316.4	×	×	centromere protein E
IPI00296388	208.4	×	×	Isoform 1 of Bromodomain adjacent to zinc finger domain 2A
IPI00296437	31.4	×	×	Type III iodothyronine deiodinase
IPI00296527	37.7	×	×	C-type lectin domain family 4 member K
IPI00296534	77.3	×	×	Isoform D of Fibulin-1 precursor
IPI00296830	120.3	×	×	Isoform 1 of Leucine zipper protein 1
IPI00296934	47.1	×	×	NF-kappa-B-activating protein
IPI00296947	87.9	×	×	PR domain zinc finger protein 4
IPI00297191	160.7	×	×	CRSP complex subunit 2

IPI00297211	121.9	×	×	SWI/SNF-related matrix-associated actin-dependent regulator of chromatin subfamily A member 5
IPI00297212	94.1	×	×	Paladin
IPI00297242	310.6	×	×	similar to CG1332-PA
IPI00297257	101.4	×	×	Trafficking kinesin-binding protein 2
IPI00297288	157	×	×	Cdc42 GTPase-activating protein
IPI00297301	59.9	×	×	Mesoderm induction early response 1, family member 2
IPI00297407	87.6	×	×	CDNA FLJ46344 fis, clone TESTI4047119
IPI00297412	152.8	×	×	Isoform 1 of Calcium-dependent secretion activator 1
IPI00297450	68.2	×	×	Uncharacterized protein C12orf26
IPI00297452	88.7	×	×	TRK-fused gene/anaplastic large cell lymphoma kinase extra long form
IPI00297465	94.5	×	×	95 kDa protein
IPI00297550	83.1	×	×	Coagulation factor XIII A chain precursor
IPI00297593	404.2	×	×	ubiquitin specific protease 34
IPI00297626	67.6	×	×	Syntaxin-binding protein 3
IPI00297655	265.4	×	×	Neurogenic locus notch homolog protein 2 precursor
IPI00297671	115.5	×	×	Cyclin-dependent kinase-like 5
IPI00297723	128.6	×	×	RNA-binding protein 6
IPI00297763	255.9	×	×	Retinal-specific ATP-binding cassette transporter
IPI00297851	196.7	×	×	chromodomain helicase DNA binding protein 1
IPI00297859	564.2	×	×	Isoform 1 of Myeloid/lymphoid or mixed-lineage leukemia protein 2
IPI00297933	166.4	×	×	Glutamate [NMDA] receptor subunit epsilon 2 precursor
IPI00298022	79.4	×	×	RING finger protein 103
IPI00298057	204.6	×	×	Periplakin
IPI00298058	121	×	×	Isoform 1 of Transcription elongation factor SPT5
IPI00298070	79.9	×	×	Isoform PDE4C1 of cAMP-specific 3',5'-cyclic phosphodiesterase 4C
IPI00298306	350.6	×	×	Serine-protein kinase ATM

IPI00298363	72.7	×	×	Far upstream element-binding protein 2
IPI00298421	53.1	×	×	Doublesex- and mab-3-related transcription factor A1
IPI00298441	60.7	×	×	Excitatory amino acid transporter 5
IPI00298447	181.7	×	×	TAR RNA binding protein 1
IPI00298518	126.5	×	×	Isoform 1 of Suppression of tumorigenicity 5
IPI00298726	52.9	×	×	Isoform 58 kDa of Mitogen-activated protein kinase kinase kinase 8
IPI00298738	140.2	×	×	Similar to DNA-directed RNA polymerase, mitochondrial precursor
IPI00298753	108	×	×	DRP2 protein
IPI00298870	142.2	×	×	Transmembrane protein 1
IPI00298883	122.1	×	×	Isoform 1 of 5-azacytidine-induced protein 1
IPI00298884	140.5	×	×	Putative RNA-binding protein 16
IPI00298902	140.9	×	×	KIAA1107 protein
IPI00298935	191.6	×	×	Isoform 1 of JmjC domain-containing histone demethylation protein 2B
IPI00299007	45.9	×	×	Phakinin
IPI00299010	88.2	×	×	Isoform 1 of Paraplegin
IPI00299048	180.6	×	×	IQ motif containing GTPase activating protein 2
IPI00299063	77.5	×	×	Stromal interaction molecule 1 precursor
IPI00299088	60.9	×	×	Isoform 1 of Brain-specific angiogenesis inhibitor 1-associated protein 2
IPI00299111	187.1	×	×	soluble adenylyl cyclase
IPI00299150	37.5	×	×	Cathepsin S precursor
IPI00299158	45.3	×	×	Isoform Alpha of Poliovirus receptor precursor
IPI00299162	59.2	×	×	V(D)J recombination-activating protein 2
IPI00299186	51.5	×	×	52 kDa protein
IPI00299254	138.8	×	×	Eukaryotic translation initiation factor 5B
IPI00299307	79.3	×	×	Complement-activating component of Ra-reactive factor precursor

IPI00299313	37.6	×	×	Isoform 1 of Ubiquitin carboxyl-terminal hydrolase isozyme L5
IPI00299404	129.6	×	×	Laminin beta-3 chain precursor
IPI00299425	60.7	×	×	Protein GPR108 precursor
IPI00299503	92.4	×	×	Phosphatidylinositol-glycan-specific phospholipase D 1 precursor
IPI00299512	319.4	×	×	Isoform 2 of Neurofibromin
IPI00299594	103.1	×	×	Isoform 1 of Neuropilin-1 precursor
IPI00299608	105.8	×	×	Isoform 1 of 26S proteasome non-ATPase regulatory subunit 1
IPI00299627	175.4	×	×	Dual oxidase 2 precursor
IPI00299635	527.6	×	×	Baculoviral IAP repeat-containing protein 6
IPI00299890	94.5	×	×	ZNF33A protein
IPI00300053	56.7	×	×	Keratin, type II cuticular Hb2
IPI00300078	102.5	×	×	Periodic tryptophan protein 2 homolog
IPI00300125	104.1	×	×	diacylglycerol kinase, zeta 104kDa isoform 2
IPI00300127	115.7	×	×	N-acetyltransferase 10
IPI00300321	83.2	×	×	Isoform 1 of Ribosomal protein S6 kinase alpha-2
IPI00300378	104.7	×	×	CDNA: FLJ23035 fis, clone LNG02033
IPI00300504	147.8	×	×	Isoform 1 of Regulator of nonsense transcripts 2
IPI00300536	69	×	×	ATG16 autophagy related 16-like 2
IPI00300789	62.6	×	×	Isoform 1 of Double-stranded RNA-binding protein Staufen homolog 2
IPI00300886	35.4	×	×	Mitochondrial folate transporter/carrier
IPI00301139	72.9	×	×	CRSP complex subunit 6
IPI00301277	70.4	×	×	Heat shock 70 kDa protein 1L
IPI00301317	132.7	×	×	Isoform 1 of Catenin delta-2
IPI00301480	134.5	×	×	134 kDa protein
IPI00301865	110.1	×	×	transmembrane protein 132A isoform b
IPI00302135	65.3	×	×	Spermatogenesis associated 16
IPI00302149	106.2	×	×	Isoform 1 of Polyhomeotic-like protein 3
IPI00302302	116.7	×	×	Isoform 2 of Homeodomain-interacting protein kinase 1

IPI00302328	223.7	×	×	Myosin heavy chain, cardiac muscle alpha isoform
IPI00302383	168.2	×	×	Cystic fibrosis transmembrane conductance regulator
IPI00302453	511.9	×	×	Ciliary dynein heavy chain 9
IPI00302503	78.7	×	×	OTTHUMP00000016947
IPI00302599	49	×	×	Isoform A of Probable tubulin polyglutamylase
IPI00302641	479.4	×	×	Protocadherin Fat 2 precursor
IPI00302647	104.1	×	×	Isoform 1 of Coiled-coil and C2 domain-containing protein 1A
IPI00302787	78.2	×	×	Serine/threonine-protein kinase PLK2
IPI00303063	150.8	×	×	SCC-112 protein
IPI00303071	59	×	×	Cat eye syndrome critical region protein 1 precursor
IPI00303112	235.3	×	×	CXXC-type zinc finger protein 6
IPI00303139	47.7	×	×	48 kDa protein
IPI00303152	161.1	×	×	collagen, type XXII, alpha 1
IPI00303313	183.6	×	×	Collagen alpha-1(V) chain precursor
IPI00303335	773.2	×	×	Nebulin
IPI00303342	22	×	×	FLJ00035 protein
IPI00303343	139.3	×	×	Serine arginine-rich pre-mRNA splicing factor SR-A1
IPI00303530	42.1	×	×	methenyltetrahydrofolate synthetase domain containing
IPI00303770	35.6	×	×	Olfactory receptor 2G2
IPI00303813	81.1	×	×	Nucleolar protein 11
IPI00303868	83.8	×	×	Glycogen [starch] synthase, muscle
IPI00303890	56.7	×	×	Baculoviral IAP repeat-containing protein 4
IPI00304023	102.7	×	×	RBBP8 protein
IPI00304028	128.4	×	×	Retinoblastoma-like protein 2
IPI00304064	196.4	×	×	Isoform 1 of InaD-like protein
IPI00304345	35.3	×	×	Isoform 2 of Signal peptide peptidase-like 2B
IPI00304435	33.3	×	×	Protein NipSnap1
IPI00304600	51.8	×	×	Isoform 3 of CaM kinase-like vesicle-associated protein
IPI00304639	425.6	×	×	Isoform 3 of Dystrophin
IPI00304648	88.4	×	×	MLCK protein

IPI00304654	239.7	×	×	similar to additional sex combs like 2 isoform 1
IPI00304692	42.3	×	×	Heterogeneous nuclear ribonucleoprotein G
IPI00304742	112.7	×	×	113 kDa protein
IPI00304817	136.7	×	×	SAM and SH3 domain-containing protein 1
IPI00304875	62	×	×	Isoform 1 of HIRA-interacting protein 3
IPI00304885	106.9	×	×	Centromere protein C 1
IPI00304993	96.1	×	×	Interleukin-17 receptor A precursor
IPI00305022	14.7	×	×	Potassium voltage-gated channel subfamily E member 1
IPI00305068	106.9	×	×	Pre-mRNA-processing factor 6
IPI00305258	37.5	×	×	Novel protein
IPI00305282	153.9	×	×	Isoform 1 of DNA repair protein RAD50
IPI00305289	119.2	×	×	Kinesin-like protein KIF11
IPI00305457	13.1	×	×	PRO2275
IPI00305518	95.6	×	×	Coiled-coil domain-containing protein 45
IPI00305703	34.9	×	×	Isoform 1 of Testisin precursor
IPI00305833	57.5	×	×	Smu-1 suppressor of mec-8 and unc-52 protein homolog
IPI00305887	54.3	×	×	Kinetochore protein Nuf2
IPI00306048	72.6	×	×	Isoform 1 of ATPase family AAA domain-containing protein 3B
IPI00306239	72.3	×	×	Katanin p80 WD40-containing subunit B1
IPI00306511	88.3	×	×	Isoform 1 of Lethal
IPI00306532	55.1	×	×	MYO15B protein
IPI00306613	33.8	×	×	TMEM116 protein
IPI00306718	175.6	×	×	Synaptonemal complex protein 2
IPI00306723	121	×	×	CCAAT/enhancer-binding protein zeta
IPI00306794	116.5	×	×	Caspase recruitment domain-containing protein 6
IPI00306851	216	×	×	Low-density lipoprotein receptor-related protein 4 precursor
IPI00306871	93.8	×	×	Isoform 1 of General control of amino acid synthesis protein 5-like 2
IPI00306929	285.5	×	×	Isoform 1 of Myosin-18B
IPI00306984	123.8	×	×	Potassium voltage-gated channel subfamily H member 8

IPI00307009	103.1	×	×	POM121 membrane glycoprotein-like 2
IPI00307016	6.1	×	×	metallothionein 1H-like protein
IPI00307114	158.3	×	×	Zinc finger protein 291
IPI00307257	140.5	×	×	TBC1 domain family, member 9B (with GRAM domain) isoform a
IPI00307569	42.5	×	×	CDNA FLJ39824 fis, clone SPLEN2011981
IPI00307592	269.9	×	×	ATP-binding cassette, sub-family A, member 2 isoform a
IPI00307611	266.1	×	×	Isoform 1 of Microtubule-associated serine/threonine-protein kinase 4
IPI00307612	89.1	×	×	Cadherin-20 precursor
IPI00307684	355.9	×	×	lupus brain antigen 1
IPI00307729	135.6	×	×	ADAMTS-3 precursor
IPI00307758	135.1	×	×	135 kDa protein
IPI00307783	72.6	×	×	Isoform 3 of Histone-lysine N-methyltransferase NSD3
IPI00307829	149.1	×	×	cingulin-like 1
IPI00328097	50.9	×	×	Sorting nexin-27
IPI00328118	134.4	×	×	Sperm-associated antigen 5
IPI00328195	327.8	×	×	Isoform 1 of Protein neurobeachin
IPI00328228	46.4	×	×	Isoform 2 of Cytohesin-1
IPI00328230	253.1	×	×	Isoform Short of Spectrin beta chain, brain 1
IPI00328268	146.9	×	×	EIF4G3 protein
IPI00328350	103.1	×	×	Niban protein
IPI00328379	76.7	×	×	G-protein signalling modulator 2
IPI00328493	15.6	×	×	Full-length cDNA clone CS0DL004YM19 of B cells (Ramos cell line) of Homo sapiens (Fragment)
IPI00328713	98.4	×	×	Zinc finger protein 546
IPI00328719	81.9	×	×	Oligopeptide transporter, kidney isoform
IPI00328736	293.4	×	×	293 kDa protein
IPI00328737	98.6	×	×	Isoform 1 of Zinc finger protein 598
IPI00328754	393.7	×	×	Zinc finger homeodomain 4 protein
IPI00328762	576.2	×	×	Isoform 1 of ATP-binding cassette sub-family A member 13

IPI00328765	89.8	×	×	Isoform 1 of Progesterone-induced-blocking factor 1
IPI00328793	123.7	×	×	Sterol regulatory element-binding protein 2
IPI00328813	71.2	×	×	Probable ATP-dependent RNA helicase DDX53
IPI00328825	198.8	×	×	Isoform 1 of NEDD4-binding protein 2
IPI00328828	147.8	×	×	AT rich interactive domain 4B isoform 1
IPI00328894	16	×	×	Hypothetical protein
IPI00328905	184.9	×	×	185 kDa protein
IPI00328929	106.4	×	×	Conserved nuclear protein NHN1
IPI00328963	44.2	×	×	Angiopoietin-related protein 5 precursor
IPI00329002	79.4	×	×	Huntingtin-interacting protein-like protein
IPI00329038	215	×	×	Isoform 1 of CDK5 regulatory subunit-associated protein 2
IPI00329084	153.7	×	×	CDNA FLJ46776 fis, clone TRACH3026650, highly similar to Actin cross- linking family protein 7
IPI00329104	47.5	×	×	Leukocyte immunoglobulin-like receptor subfamily A member 3 precursor
IPI00329192	12.9	×	×	C14orf125 protein
IPI00329245	104.1	×	×	KIAA0692 protein
IPI00329291	123.9	×	×	Isoform 2 of StAR-related lipid transfer protein 13
IPI00329327	443.6	×	×	Isoform 2 of Extracellular matrix protein FRAS1 precursor
IPI00329331	56.9	×	×	UDP-glucose pyrophosphorylase 2 isoform a
IPI00329383	61.5	×	×	Suppressor of hairy wing homolog 2
IPI00329420	37.8	×	×	serine (or cysteine) proteinase inhibitor, clade A (alpha-1 antiproteinase, antitrypsin), member 9 isoform B
IPI00329517	62.1	×	×	OTTHUMP00000022161
IPI00329528	163.1	×	×	VPRBP protein
IPI00329556	192	×	×	hypothetical protein LOC80217
IPI00329583	97.9	×	×	CDNA FLJ34065 fis, clone FCBBF3000536, highly similar to Mus musculus spermatogenesis associated factor (SPAF) mRNA
IPI00329590	53.2	×	×	Isoform 1 of Suppressor of SWI4 1 homolog
IPI00329591	51.6	×	×	Isoform 1 of Protein C6orf96

IPI00329598	33	×	×	Dehydrogenase/reductase SDR family member 8 precursor
IPI00329605	127.5	×	×	DNA mismatch repair protein Msh3
IPI00329629	56.4	×	×	DnaJ homolog subfamily C member 7
IPI00329637	103.2	×	×	Uncharacterized protein C1orf26
IPI00329668	118.1	×	×	similar to DAB2 interacting protein isoform 2
IPI00329692	56.8	×	×	Isoform Long of Glycylpeptide N-tetradecanoyltransferase 1
IPI00329719	116.2	×	×	Isoform 1 of Myosin Id
IPI00329775	48.4	×	×	Isoform 1 of Carboxypeptidase B2 precursor
IPI00332155	102.4	×	×	Isoform 1 of G protein-regulated inducer of neurite outgrowth 1
IPI00332552	138.1	×	×	Isoform 1 of Zinc finger and BTB domain-containing protein 40
IPI00332845	69.7	×	×	70 kDa protein
IPI00333014	46.4	×	×	Isoform 1 of Protein C13orf3
IPI00333314	68.7	×	×	Zinc finger protein 85
IPI00333541	280.6	×	×	Filamin-A
IPI00333753	313.9	×	×	Isoform 1 of Inositol 1,4,5-trisphosphate receptor type 1
IPI00333770	249.5	×	×	Isoform 1 of Dedicator of cytokinesis protein 10
IPI00333828	47	×	×	Serpin A11 precursor
IPI00334013	80.6	×	×	Protein C6orf152
IPI00334532	139.5	×	×	Isoform 2 of Neural cell adhesion molecule L1 precursor
IPI00334627	38.7	×	×	Similar to annexin A2 isoform 1
IPI00335085	148.5	×	×	Isoform 1 of E3 ubiquitin-protein ligase RNF123
IPI00335158	247.6	×	×	248 kDa protein
IPI00335437	80.9	×	×	Ankyrin repeat and zinc finger domain-containing protein 1
IPI00335443	49.7	×	×	Isoform 2 of Sperm-associated antigen 6
IPI00335509	61.4	×	×	Dihydropyrimidinase-related protein 5
IPI00335541	138.6	×	×	Isoform 1 of Timeless homolog
IPI00335824	187	×	×	Isoform Short of Tight junction protein ZO-1
IPI00337325	84	×	×	Isoform A of Hyaluronan mediated motility receptor
IPI00337335	228.7	×	×	myosin, heavy chain 14 isoform 1

IPI00337385	110.4	×	×	similar to Pre-mRNA-processing factor 40 homolog A (Formin-binding protein 3) (Huntingtin yeast partner A) (Huntingtin-interacting protein HYPA/FBP11) (Fas ligand-associated factor 1) (NY-REN-6 antigen) isoform 3
IPI00337766	91.7	×	×	zinc finger, CCHC domain containing 2
IPI00337790	142	×	×	Isoform 2 of Ras GTPase-activating protein SynGAP
IPI00337800	123.5	×	×	Isoform 1 of Disheveled-associated activator of morphogenesis 1
IPI00337834	103.1	×	×	transcription termination factor, RNA polymerase I
IPI00339274	13.9	×	×	Histone H2A type 2-C
IPI00339309	107.7	×	×	Isoform 1 of Proto-oncogene DBL
IPI00339381	113.9	×	×	Isoform 1 of SWI/SNF-related matrix-associated actin-dependent regulator of chromatin subfamily A member 3
IPI00373807	13.5	×	×	similar to H2A histone family, member V isoform 2
IPI00373870	226.6	×	×	Isoform 1 of Chromodomain helicase-DNA-binding protein 3
IPI00373894	253.7	×	×	Isoform 1 of UPF0378 family protein KIAA0100 precursor
IPI00374186	40.7	×	×	CDNA FLJ45645 fis, clone CTONG2003517
IPI00374260	24.6	×	×	similar to ribosomal protein L10 isoform 1
IPI00374531	70.5	×	×	Isoform 2 of Zinc finger CCHC domain-containing protein 6
IPI00374564	37.1	×	×	SUDS3 protein
IPI00374590	49.5	×	×	cancer susceptibility candidate 4 isoform a
IPI00374862	84.5	×	×	Isoform 1 of Kelch-like protein 5
IPI00374973	203.4	×	×	ankyrin 1 isoform 4
IPI00375149	110.7	×	×	Isoform 8 of Dynamin-like 120 kDa protein, mitochondrial precursor
IPI00375239	15.7	×	×	Hypothetical protein
IPI00375254	123.4	×	×	F-box only protein, helicase, 18 isoform 1
IPI00375294	337.2	×	×	Laminin alpha-1 chain precursor
IPI00375330	132.9	×	×	Isoform 1 of Wings apart-like protein homolog

IPI00375339	114.2	×	×	Isoform 1 of Sodium/potassium-transporting ATPase alpha-4 chain
IPI00375358	128.3	×	×	Isoform 1 of Replication factor C subunit 1
IPI00375402	86.8	×	×	87 kDa protein
IPI00375604	48	×	×	male-specific lethal 3-like 1 isoform d
IPI00375638	101	×	×	SAPS domain family member 2
IPI00375731	110.3	×	×	Hypothetical protein DKFZp686E2459
IPI00375737	8.7	×	×	9 kDa protein
IPI00376045	71.4	×	×	Isoform 1 of AMY-1-associating protein expressed in testis 1
IPI00376259	124.3	×	×	Isoform 1 of SLIT-ROBO Rho GTPase-activating protein 1
IPI00376321	95.5	×	×	Rho GTPase activating protein 30 isoform 2
IPI00376344	132	×	×	Isoform 1 of Myosin Ib
IPI00376383	205.2	×	×	Centrosomal protein 1
IPI00376439	448.7	×	×	Isoform 1 of Vacuolar protein sorting-associated protein 13B
IPI00376529	9.9	×	×	10 kDa protein
IPI00376941	100.4	×	×	Isoform 1 of Probable phospholipase DDHD1
IPI00376955	59.6	×	×	Serine/threonine-protein kinase PCTAIRE-2
IPI00377045	372.7	×	×	Alpha3A
IPI00377071	158.2	×	×	Isoform 1 of Protein phosphatase Slingshot homolog 2
IPI00377122	91.3	×	×	KIAA1875 protein
IPI00382422	10.9	×	×	Ig lambda chain V-I region NEWM
IPI00382426	11.6	×	×	Ig lambda chain V-II region TRO
IPI00382432	143.6	×	×	Isoform 1 of N-acetylglucosamine-1-phosphotransferase subunits alpha/beta precursor
IPI00382455	12.5	×	×	Ig heavy chain V-I region EU
IPI00382476	12.3	×	×	Ig heavy chain V-III region WEA
IPI00382483	13.2	×	×	Ig heavy chain V-III region GA
IPI00382486	13.2	×	×	Ig heavy chain V-III region NIE
IPI00382497	12.4	×	×	Ig heavy chain V-III region TUR
IPI00382500	12.7	×	×	Ig heavy chain V-III region GAL

IPI00382534	14.3	×	×	Ig heavy chain V-II region OU
IPI00382844	65.3	×	×	Aconitase (Fragment)
IPI00383016	11.5	×	×	Immunglobulin light chain variable region (Fragment)
IPI00383105	326.1	×	×	Isoform 1 of Chromodomain-helicase-DNA-binding protein 9
IPI00383133	182.5	×	×	BCL6 co-repressor-like 1
IPI00383161	88.4	×	×	88 kDa protein
IPI00383165	24.5	×	×	HSPC123
IPI00383244	33	×	×	Metaphase chromosomal protein 1
IPI00383372	9.3	×	×	PRO1155
IPI00383474	57.7	×	×	Isoform 1 of Butyrophilin subfamily 3 member A1 precursor
IPI00383508	119.8	×	×	Isoform 3 of Protein SMG7
IPI00383585	52.4	×	×	BTB/POZ domain-containing protein KCTD8
IPI00383695	24	×	×	Mitochondrial trifunctional protein beta subunit (Fragment)
IPI00383729	39.5	×	×	U7 snRNA-associated Sm-like protein LSm11
IPI00383732	15.8	×	×	VH3 protein (Fragment)
IPI00383825	61.4	×	×	Hypothetical protein W80
IPI00383866	179.4	×	×	Retinoblastoma binding protein 2 homolog 1
IPI00383887	12.2	×	×	Immunoblobulin heavy chain (Fragment)
IPI00383904	21.7	×	×	CDNA FLJ37981 fis, clone CTONG2010566
IPI00383931	76.6	×	×	CDNA FLJ37317 fis, clone BRAMY2017455, highly similar to Homo sapiens ATP-binding cassette protein M-ABC1 mRNA
IPI00383953	16.3	×	×	VH4 heavy chain variable region precursor (Fragment)
IPI00383957	17.3	×	×	VH1 protein precursor (Fragment)
IPI00383995	28.6	×	×	Isoform 2 of Protein C10orf93
IPI00384089	18.9	×	×	KFSP2566
IPI00384172	141.6	×	×	Beta I spectrin form betaI sigma3
IPI00384250	89.2	×	×	cyclic AMP-regulated phosphoprotein, 21 kD isoform 1
IPI00384297	25.3	×	×	carbonic reductase 4
IPI00384392	12.9	×	×	Myosin-reactive immunoglobulin heavy chain variable region (Fragment)

IPI00384398	11.5	×	×	Myosin-reactive immunoglobulin light chain variable region (Fragment)
IPI00384399	11.4	×	×	Myosin-reactive immunoglobulin light chain variable region (Fragment)
IPI00384400	13.6	×	×	Myosin-reactive immunoglobulin heavy chain variable region (Fragment)
IPI00384407	13.6	×	×	Myosin-reactive immunoglobulin heavy chain variable region (Fragment)
IPI00384441	27.5	×	×	Sarcoma antigen NY-SAR-48 (Fragment)
IPI00384444	51.5	×	×	Keratin, type I cytoskeletal 14
IPI00384479	48.5	×	×	C12orf40 protein
IPI00384529	135.6	×	×	Isoform 1 of Protein cordon-bleu
IPI00384707	60	×	×	EARS2 protein
IPI00384783	20.6	×	×	CDNA FLJ45402 fis, clone BRHIP3029409, moderately similar to Homo sapiens secreted frizzled-related protein 1
IPI00384831	17	×	×	CDNA FLJ37699 fis, clone BRHIP2016788, weakly similar to Mus musculus left-right dynein (Lrd) mRNA
IPI00384972	121	×	×	Isoform 1 of Protein KIAA1267
IPI00385055	105.4	×	×	Alpha-2 catenin
IPI00385065	112.7	×	×	Isoform 1 of Coiled-coil domain-containing protein 46
IPI00385066	38.7	×	×	MGC45780
IPI00385143	14.1	×	×	Microfibrillar protein 2 (Fragment)
IPI00385252	11.8	×	×	Ig kappa chain V-III region GOL
IPI00385253	14.3	×	×	Ig kappa chain V-III region CLL precursor
IPI00385254	11.7	×	×	Ig lambda chain V-I region WAH
IPI00385264	43.1	×	×	Ig mu heavy chain disease protein
IPI00385321	66.9	×	×	Isoform 2 of Protein kinase C-binding protein 1
IPI00385480	149.9	×	×	Caskin-1
IPI00385511	186.7	×	×	Isoform 1 of Trinucleotide repeat-containing 6B protein
IPI00385578	51.9	×	×	HDCMC04P

IPI00385614	11.7	×	×	Ig kappa chain V-I region WAT
IPI00385757	16.2	×	×	CDNA FLJ35506 fis, clone SMINT2009415
IPI00385859	54.8	×	×	CDNA FLJ10866 fis, clone NT2RP4001614
IPI00385869	17.2	×	×	DC27
IPI00385910	111.5	×	×	SNCAIP protein
IPI00386072	47.1	×	×	Isoform 1 of Alpha-1,3-mannosyltransferase ALG2
IPI00386134	13.6	×	×	Ig lambda chain V-I region BL2 precursor
IPI00386140	13.6	×	×	Ig heavy chain V-I region Mot
IPI00386189	101.3	×	×	Isoform 1 of NMDA receptor-regulated protein 1
IPI00386211	330.5	×	×	hypothetical protein LOC259282
IPI00386364	35.5	×	×	Olfactory receptor 2L2
IPI00386442	91.2	×	×	Kinesin-associated protein 3
IPI00386524	53.5	×	×	CDNA FLJ25298 fis, clone STM07683, highly similar to Protein Tro alpha1 H,myeloma
IPI00386572	26.9	×	×	C20orf74 protein
IPI00386687	71.8	×	×	LRRFIP1 protein
IPI00386763	216.6	×	×	Isoform 1 of ADAMTS-9 precursor
IPI00386839	12.3	×	×	Amyloid lambda 6 light chain variable region SAR (Fragment)
IPI00386879	53.1	×	×	CDNA FLJ14473 fis, clone MAMMA1001080, highly similar to Homo sapiens SNC73 protein (SNC73) mRNA
IPI00386981	109.7	×	×	HERV-K_3q27.3 provirus ancestral Pol protein
IPI00387024	11.7	×	×	Ig kappa chain V-I region CAR
IPI00387077	96.1	×	×	solute carrier family 12 (potassium/chloride transporters), member 9
IPI00387079	23.5	×	×	HT016
IPI00387105	11.9	×	×	Ig kappa chain V-I region Mev
IPI00387106	12.2	×	×	Ig kappa chain V-I region Ni
IPI00387111	12.8	×	×	13 kDa protein
IPI00387116	10.7	×	×	Ig kappa chain V-III region NG9 precursor (Fragment)
IPI00387117	14	×	×	Similar to Ig kappa chain V-III region HAH precursor

IPI00387119	11.9	×	×	Ig kappa chain V-III region POM
IPI00387144	50.2	×	×	Tubulin alpha-ubiquitous chain
IPI00387161	11.5	×	×	Ig lambda chain V-I region VOR
IPI00387168	74.4	×	×	Isoform 1 of Proprotein convertase subtilisin/kexin type 9 precursor
IPI00394665	136	×	×	Isoform 1 of Double-stranded RNA-specific adenosine deaminase
IPI00394793	111.2	×	×	pleckstrin homology domain containing family G member 5 isoform a
IPI00394926	51.4	×	×	DNA polymerase subunit delta 3
IPI00395554	73.5	×	×	Forkhead box protein P4
IPI00395649	281.2	×	×	281 kDa protein
IPI00395726	55.2	×	×	PRAME family member 1
IPI00395771	36.8	×	×	Isoform 2 of Protein phosphatase 1 regulatory subunit 7
IPI00395925	122.7	×	×	WWC family member 3
IPI00396058	132.2	×	×	Isoform 2 of Condensin-II complex subunit G2
IPI00396063	213.7	×	×	Similar to IRLB
IPI00396130	108	×	×	Isoform 2 of p130Cas-associated protein
IPI00396174	24.5	×	×	Coiled-coil domain-containing protein 25
IPI00396185	83.6	×	×	corneal wound healing-related protein
IPI00396218	103.7	×	×	SCY1-like protein 2
IPI00396243	151.8	×	×	Isoform 1 of WD repeat protein 19
IPI00396286	59.6	×	×	Isoform 1 of Dual specificity tyrosine-phosphorylation-regulated kinase 4
IPI00396343	115.1	×	×	Isoform 1 of Kinesin-like protein KIF17
IPI00397430	28.6	×	×	LOC54103 protein
IPI00397526	228.9	×	×	Myosin-10
IPI00397768	73	×	×	Isoform 2 of Ribonucleoprotein PTB-binding 2
IPI00398012	555.3	×	×	Fragile site-associated protein
IPI00398162	198	×	×	Isoform 2 of Nebulin-related anchoring protein
IPI00398268	44.3	×	×	similar to melanoma antigen family B, 6

IPI00398310	78.2	×	×	ZNF573 protein
IPI00398364	133.5	×	×	Isoform 1 of Liprin-alpha-3
IPI00398421	130.5	×	×	potassium channel, subfamily T, member 2
IPI00398435	211.9	×	×	similar to Plexin-B2 precursor
IPI00398823	50.6	×	×	Isoform 3 of Zinc phosphodiesterase ELAC protein 2
IPI00398940	83.1	×	×	IQ motif and Sec7 domain 3
IPI00399164	132.3	×	×	Isoform 1 of AT-rich interactive domain-containing protein 5B
IPI00399252	95.5	×	×	Isoform 1 of Protein Jade-1
IPI00399337	35.7	×	×	Olfactory receptor 6C1
IPI00400836	10.5	×	×	Conserved hypothetical protein
IPI00400873	302.3	×	×	similar to retinoblastoma-associated protein 140 isoform 1
IPI00400922	208.7	×	×	RRP5 protein homolog
IPI00400923	121.8	×	×	SH3 multiple domains 1
IPI00401201	131	×	×	similar to Zinc finger protein 74
IPI00401270	53.6	×	×	X Kell blood group precursor-related, X-linked
IPI00401276	114.2	×	×	Phosphodiesterase 4D interacting protein
IPI00401282	146.5	×	×	similar to Golgin subfamily A member 6
IPI00401611	193.7	×	×	similar to RW1 protein isoform 1
IPI00401829	97.4	×	×	Putative uncharacterized protein ENST00000281581
IPI00401956	148.3	×	×	similar to apoptosis-associated tyrosine kinase
IPI00401962	33.2	×	×	MGC16597 protein
IPI00402037	66.2	×	×	similar to C32D5.6 isoform 1
IPI00409579	120.6	×	×	Isoform 2 of Glutamate receptor-interacting protein 1
IPI00409607	168.1	×	×	Novel protein
IPI00409756	41.1	×	×	L-myc-2 protein
IPI00410039	52.7	×	×	Isoform 1 of Periphilin-1
IPI00410096	197.6	×	×	selective LIM binding factor homolog
IPI00410110	88.6	×	×	Isoform 1 of Probable ATP-dependent RNA helicase DHX40
IPI00410121	166.5	×	×	IMP dehydrogenase/GMP reductase family protein
IPI00410188	112.8	×	×	hypothetical protein LOC57639

IPI00410321	73.2	×	×	multiple coiled-coil GABABR1-binding protein
IPI00410323	60.5	×	×	Isoform 1 of Thioredoxin domain-containing protein 2
IPI00410334	65.3	×	×	Sulfate/anion exchanger
IPI00410485	105.4	×	×	Serine/threonine-protein kinase TAO3
IPI00411291	142.9	×	×	Peroxisome biogenesis factor 1
IPI00411452	238.1	×	×	Dedicator of cytokinesis 11
IPI00411635	179.6	×	×	180 kDa protein
IPI00411656	566.7	×	×	Isoform 1 of Protein piccolo
IPI00411901	202.1	×	×	Isoform 1 of Discs large homolog 5
IPI00412146	99.1	×	×	Cone cGMP-specific 3',5'-cyclic phosphodiesterase alpha'-subunit
IPI00412269	51.9	×	×	Equilibrative nucleoside transporter 3
IPI00412298	301.4	×	×	Isoform 1 of Serine/threonine-protein kinase ATR
IPI00412408	384.2	×	×	Breast cancer type 2 susceptibility protein
IPI00412494	23.2	×	×	Isoform 1 of Claudin-19
IPI00412541	135.5	×	×	Probable G-protein coupled receptor 158 precursor
IPI00413300	17.7	×	×	LOC595101 protein
IPI00413492	74.1	×	×	calpastatin isoform b
IPI00413604	202.6	×	×	Isoform 4 of Myosin light chain kinase, smooth muscle
IPI00413659	52.5	×	×	Isoform 2 of Alkylated repair protein alkB homolog 5
IPI00413755	110.1	×	×	Transcription initiation factor TFIID subunit 4
IPI00413868	133	×	×	Chromosome 6 open reading frame 60
IPI00414008	70.4	×	×	Leiomodin 1
IPI00414138	42.3	×	×	Isoform 2 of Alpha-(1,3)-fucosyltransferase
IPI00414481	213	×	×	GTF3C1 protein
IPI00414779	168.4	×	×	DNA excision repair protein ERCC-6
IPI00415037	99.6	×	×	Isoform 12L of ADAM 12 precursor
IPI00418137	34.7	×	×	POTE15 protein
IPI00418220	139	×	×	Isoform 1 of Zinc finger protein 406
IPI00418277	100.4	×	×	Chondroitin sulfate synthase 3

IPI00418422	63.4	×	×	IGHD protein
IPI00418464	69	×	×	69 kDa protein
IPI00418557	77.4	×	×	Transmembrane and coiled-coil domains protein 2
IPI00418592	43.6	×	×	CD1E antigen isoform a precursor
IPI00418663	53	×	×	CDNA FLJ26317 fis, clone DMC09625
IPI00418760	75.4	×	×	CDNA FLJ46362 fis, clone TESTI4050293
IPI00418765	314.7	×	×	similar to otogelin
IPI00418780	132.3	×	×	Isoform 3 of Regulator of G-protein signaling 3
IPI00418791	135.2	×	×	sperm-specific sodium proton exchanger
IPI00418991	143.7	×	×	CDNA FLJ44035 fis, clone TESTI4028612
IPI00419084	149.3	×	×	CDNA FLJ43333 fis, clone NT2RI3006376
IPI00419219	53.4	×	×	CD44 antigen isoform 3 precursor
IPI00419234	60.6	×	×	Glycerol kinase, testis specific 2
IPI00419286	45.3	×	×	Isoform 1 of EMI domain-containing protein 1 precursor
IPI00419431	153.7	×	×	Hypothetical protein DKFZp686C1968
IPI00419504	76.5	×	×	Isoform 1 of Mitogen-activated protein kinase kinase kinase 7-interacting protein 2
IPI00419509	177.5	×	×	peroxisome proliferator-activated receptor gamma, coactivator-related 1
IPI00419518	121.1	×	×	Ankyrin repeAt domAin 32
IPI00419535	90.3	×	×	Leucine-rich repeat-containing protein 8E
IPI00419542	84.1	×	×	Isoform 2 of Hormone-sensitive lipase
IPI00419565	275.5	×	×	Isoform 1 of Stabilin-1 precursor
IPI00419579	52.4	×	×	cytochrome P450, family 20, subfamily A, polypeptide 1 isoform 1
IPI00419693	136.4	×	×	activating transcription factor 7 interacting protein
IPI00419833	13.8	×	×	Histone H2B type 1-K
IPI00419922	77.4	×	×	IQ motif containing E
IPI00419928	44.6	×	×	Hypothetical protein LOC339745
IPI00420014	244.5	×	×	U5 small nuclear ribonucleoprotein 200 kDa helicase

IPI00423565	89.6	×	×	Isoform 3 of Ubiquitin carboxyl-terminal hydrolase 6
IPI00424460	168.8	×	×	Isoform 1 of Zinc finger FYVE domain-containing protein 16
IPI00425407	182.2	×	×	Isoform 3 of Kinesin family member 21A
IPI00425560	174.9	×	×	Isoform 1 of Protein LAP4
IPI00426267	172.6	×	×	Isoform 1 of Leucine-rich repeat-containing protein 7
IPI00427522	168.4	×	×	Peroxisome proliferator-activated receptor-binding protein
IPI00427808	156.5	×	×	hypothetical protein LOC118461
IPI00427822	144.5	×	×	Isoform 3 of ADAMTS-13 precursor
IPI00428401	160.1	×	×	160 kDa protein
IPI00428447	141.4	×	×	mitochondrial tumor suppressor 1 isoform 1
IPI00429689	35.6	×	×	Serine/threonine-protein phosphatase 2A catalytic subunit beta isoform
IPI00430195	51.5	×	×	Isoform 1 of Alpha-1A adrenergic receptor
IPI00430472	251.5	×	×	Isoform 1 of Activating signal cointegrator 1 complex subunit 3
IPI00432363	670.2	×	×	Microtubule-actin cross-linking factor 1, isoform 4
IPI00433029	21.8	×	×	Insulin-like growth factor IB precursor
IPI00433284	70.1	×	×	D-glucuronyl C5-epimerase
IPI00433513	154.7	×	×	leucine-rich repeats and IQ motif containing 1
IPI00434539	34.6	×	×	Isoform 1 of Homeobox protein NANOG
IPI00435422	197.5	×	×	Isoform 2 of Signal-induced proliferation-associated 1-like protein 1
IPI00436021	91.8	×	×	Titin (Fragment)
IPI00436632	316.1	×	×	Isoform 1 of Nipped-B-like protein
IPI00436705	107.3	×	×	MORC family CW-type zinc finger protein 3
IPI00437186	149.5	×	×	Isoform 1 of Probable G-protein coupled receptor 116 precursor
IPI00438229	88.5	×	×	tripartite motif-containing 28 protein
IPI00438287	153.9	×	×	Isoform 2 of Protein LAP2
IPI00438355	300.4	×	×	Fibrillin-3 precursor
IPI00440727	152.2	×	×	Isoform 1 of Bromodomain-containing protein 4

IPI00441473	72.6	×	×	Protein arginine N-methyltransferase 5
IPI00441515	169.9	×	×	Neurexin-3-alpha precursor
IPI00441867	32.8	×	×	Isoform 1 of Peroxisomal biogenesis factor 19
IPI00442025	119.3	×	×	tyrosine kinase, non-receptor, 2 isoform 2
IPI00442035	66.2	×	×	CDNA FLJ16543 fis, clone OCBBF3002654, highly similar to Triple functional domain protein
IPI00442053	81.2	×	×	Isoform 2 of Rho-GTPase-activating protein 9
IPI00442274	101.3	×	×	nuclear transcription factor, X-box binding-like 1
IPI00444272	123.7	×	×	Leukemia inhibitory factor receptor precursor
IPI00444371	101.4	×	×	Isoform 1 of WD repeat protein 44
IPI00444592	106	×	×	Isoform 1 of Trafficking kinesin-binding protein 1
IPI00445717	40.9	×	×	Isoform 2 of GDNF family receptor alpha-3 precursor
IPI00448121	63.9	×	×	Cell division cycle 7-related protein kinase
IPI00449049	113	×	×	Poly [ADP-ribose] polymerase 1
IPI00450780	12.6	×	×	CD45 transcript variant
IPI00451624	71.4	×	×	Isoform 1 of Cartilage acidic protein 1 precursor
IPI00451941	33.7	×	×	tRNA-splicing endonuclease subunit Sen34
IPI00452247	159.8	×	×	Isoform 2 of Kinesin-like motor protein C20orf23
IPI00452465	75.7	×	×	Isoform 2 of E3 ubiquitin-protein ligase DZIP3
IPI00452727	24.1	×	×	Isoform 1 of Transcription cofactor HES-6
IPI00454858	123.4	×	×	similar to alpha 3 type VI collagen isoform 1 precursor
IPI00455165	48.1	×	×	Isoform 1 of Pannexin-1
IPI00455210	220.8	×	×	Isoform 2 of Chromodomain helicase-DNA-binding protein 4
IPI00455835	47.3	×	×	Pleckstrin homology domain-containing family A member 2
IPI00455851	125	×	×	KIAA1688 protein
IPI00455982	65.7	×	×	High mobility group protein 2-like 1
IPI00456157	46.3	×	×	Isoform 1 of Decaprenyl-diphosphate synthase subunit 1
IPI00456624	71.7	×	×	Isoform 2 of Brevican core protein precursor
IPI00456626	76.5	×	×	Beta-taxilin
IPI00456678	107.7	×	×	Isoform 3 of F-box only protein 38

IPI00456708	213.1	×	×	centrosomal protein 192kDa
IPI00456887	85.1	×	×	Scaffold attachment factor A2
IPI00456907	61.5	×	×	zinc finger protein 498
IPI00456919	481.9	×	×	Isoform 1 of HECT, UBA and WWE domain-containing protein 1
IPI00456994	91.5	×	×	Hypothetical protein DKFZp434N2321
IPI00464978	137.4	×	×	Insulin receptor substrate 2 insertion mutant (Fragment)
IPI00464980	133.1	×	×	Isoform 1 of Paired amphipathic helix protein Sin3b
IPI00465045	171.5	×	×	DIP2 disco-interacting protein 2 homolog B
IPI00465050	113.4	×	×	Isoform C of Lethal(2) giant larvae protein homolog 2
IPI00465061	93.2	×	×	Hypothetical protein FLJ10081 (Fragment)
IPI00465087	80.4	×	×	Ubiquitin carboxyl-terminal hydrolase BAP1
IPI00465102	261.5	×	×	DNA polymerase epsilon, catalytic subunit A
IPI00465142	110.4	×	×	Protein KIAA0528
IPI00465164	157.7	×	×	KIAA0194 protein (Fragment)
IPI00465167	84.7	×	×	Isoform 1 of Centaurin-beta 5
IPI00465186	211.7	×	×	protein tyrosine phosphatase, receptor type, F isoform 2 precursor
IPI00465191	152.5	×	×	Hypothetical protein DKFZp686J0330
IPI00465213	39.7	×	×	annexin A13 isoform b
IPI00465246	166.4	×	×	Hypothetical protein DKFZp761D20121 (Fragment)
IPI00465271	24.1	×	×	HYI protein
IPI00465310	84	×	×	SLC30A5
IPI00465319	85.5	×	×	Chondroitin sulfate synthase 2
IPI00465323	67.2	×	×	GFHL3075
IPI00465460	65.8	×	×	Dual specificity tyrosine-phosphorylation-regulated kinase 3
IPI00470464	110.9	×	×	Isoform 1 of Zinc finger protein 694
IPI00470478	373.9	×	×	374 kDa protein
IPI00470519	91.9	×	×	Isoform 2 of Mitotic spindle assembly checkpoint protein MAD1
IPI00470582	103.7	×	×	Isoform 1 of Protein C10orf118
IPI00470596	220.6	×	×	Isoform 1 of Zinc finger protein 638

IPI00470597	123.1	×	×	ubiquitin protein ligase E3B
IPI00470627	193.5	×	×	SNF2 histone linker PHD RING helicase isoform a
IPI00470635	57.2	×	×	57 kDa protein
IPI00470637	70.1	×	×	Long-chain fatty acid transport protein 6
IPI00470648	81.5	×	×	Prestin
IPI00470838	106.9	×	×	Isoform 1 of DENN domain-containing protein 2C
IPI00470867	146.7	×	×	Hypothetical protein DKFZp686C0668
IPI00470912	224.3	×	×	Hypothetical protein DKFZp781D2023
IPI00470937	164.2	×	×	OTTHUMP00000017181
IPI00472110	49.6	×	×	Complement component (3b/4b) receptor 1-like
IPI00472202	130.8	×	×	integrin, alpha 1 precursor
IPI00472263	156.3	×	×	RNA polymerase III transcription initiation factor B''
IPI00472426	101.7	×	×	BC331191_1
IPI00472675	228.1	×	×	228 kDa protein
IPI00472779	480.4	×	×	Ankyrin-3
IPI00472810	74.8	×	×	Isoform 2 of Ubiquitin-protein ligase E3C
IPI00472901	29.8	×	×	CDNA FLJ20361 fis, clone HEP16789
IPI00472977	110.1	×	×	Ubiquitin carboxyl-terminal hydrolase 37
IPI00473031	39.7	×	×	Alcohol dehydrogenase 1B
IPI00473040	13.9	×	×	FKSG57
IPI00477245	119.6	×	×	sodium channel associated protein 2 isoform a
IPI00477535	133.3	×	×	DNA-repair protein complementing XP-G cells
IPI00477825	60.7	×	×	Isoform 1 of Mesoderm induction early response protein 1
IPI00477868	61.3	×	×	LAMA5 protein
IPI00477971	68.3	×	×	WD repeat protein SAZD
IPI00477992	26.7	×	×	complement component 1, q subcomponent, B chain precursor
IPI00478037	49.7	×	×	Zinc finger protein 358
IPI00478371	47	×	×	Isoform 1 of Ligand-dependent corepressor
IPI00478529	194.7	×	×	Isoform 9 of Transient receptor potential cation channel subfamily M member 3

IPI00478616	70.2	×	×	hypothetical protein LOC63901
IPI00478832	90.8	×	×	Fas (TNFRSF6) binding factor 1
IPI00478916	116.4	×	×	DNA cross-link repair 1A protein
IPI00479385	69.8	×	×	Isoform 1 of N-acetylserotonin O-methyltransferase-like protein
IPI00479572	123.9	×	×	Urf-ret protein
IPI00479688	95	×	×	similar to TBC1 domain family member 12
IPI00480027	127.4	×	×	1A6/DRIM (down-regulated in metastasis) interacting protein
IPI00514047	45.5	×	×	Novel protein similar to beta-tubulin 4Q
IPI00514071	196.4	×	×	Isoform 1 of Microtubule-associated serine/threonine-protein kinase 2
IPI00514475	43.9	×	×	Isoform 1 of Apolipoprotein-L1 precursor
IPI00514533	93.6	×	×	145 kDa nucleolar protein
IPI00514622	124.7	×	×	Ran-binding protein 6
IPI00514769	113	×	×	ankyrin repeat domain 28
IPI00514983	96.9	×	×	Isoform Alpha of Heat-shock protein 105 kDa
IPI00549513	34.4	×	×	RING finger protein 148 precursor
IPI00549540	44	×	×	p21-activated protein kinase-interacting protein 1
IPI00549543	78.9	×	×	neurochondrin isoform 1
IPI00549673	62.3	×	×	Armadillo repeat-containing X-linked protein 5
IPI00549822	472	×	×	Isoform 5 of Obscurin
IPI00549869	76.2	×	×	CDNA FLJ30936 fis, clone FEBRA2007200, weakly similar to MYOSIN II HEAVY CHAIN, NON MUSCLE
IPI00550035	32.7	×	×	Four and a half LIM domains protein 5
IPI00550037	29.8	×	×	28S ribosomal protein S15, mitochondrial precursor
IPI00550044	67.9	×	×	Isoform 2 of Zinc finger protein 317
IPI00550090	180.7	×	×	similar to jumonji domain containing 3 isoform 1
IPI00550232	379.2	×	×	CMYA3
IPI00550272	82.2	×	×	Integrator complex subunit 10
IPI00550361	55.3	×	×	Adipose triglyceride lipase

IPI00550477	103.3	×	×	Evolutionarily conserved G-patch domain containing protein
IPI00550496	89.9	×	×	Isoform 1 of Ring finger protein 10
IPI00550703	108.2	×	×	Isoform 1 of GPI ethanolamine phosphate transferase 2
IPI00550781	21	×	×	PRKR interacting protein 1
IPI00550834	58.8	×	×	CDNA FLJ36288 fis, clone THYMU2003650
IPI00551038	48.8	×	×	Isoform 3 of Actin-binding LIM protein 1
IPI00552074	149.5	×	×	149 kDa protein
IPI00552545	230.8	×	×	Dedicator of cytokinesis protein 8
IPI00552578	13.5	×	×	Serum amyloid A protein precursor
IPI00552897	226.7	×	×	Isoform 1 of Mediator of DNA damage checkpoint protein 1
IPI00554786	54.7	×	×	Thioredoxin reductase 1, cytoplasmic precursor
IPI00604551	42.6	×	×	Isoform 1 of Cell division cycle-associated protein 7
IPI00604620	76.6	×	×	nucleolin
IPI00607591	66.3	×	×	RAP1, GTP-GDP dissociation stimulator 1
IPI00640326	59.3	×	×	zinc finger protein 75
IPI00640653	14.8	×	×	CDNA FLJ31480 fis, clone NT2NE2001648
IPI00640762	31.9	×	×	Novel protein similar to esterases
IPI00641240	95.2	×	×	PHLPPL protein
IPI00641977	59.6	×	×	MHC class II transactivator CIITAmed
IPI00642186	165.2	×	×	Intraflagellar transport 140 homolog
IPI00642204	93.6	×	×	Thioredoxin-domain containing protein KIAA1344 precursor
IPI00642409	40.6	×	×	HLA class I histocompatibility antigen, Cw-7 alpha chain precursor
IPI00642705	218.3	×	×	Isoform 2 of AT-rich interactive domain-containing protein 1A
IPI00642716	225.8	×	×	myosin, heavy polypeptide 7B, cardiac muscle, beta
IPI00643365	55.7	×	×	KIAA0266 protein
IPI00643572	21.6	×	×	Zinc finger protein SBZF3
IPI00644346	104.6	×	×	ADAMTS-like protein 2 precursor
IPI00645901	44.9	×	×	Chromosome 1 open reading frame 94
IPI00646010	167.3	×	×	167 kDa protein

IPI00647347	112.4	×	×	similar to Carcinoembryonic antigen-related cell adhesion molecule 1 precursor
IPI00647558	77.8	×	×	FOXRED2 protein
IPI00647650	41.6	×	×	Eukaryotic translation initiation factor 3 subunit 3
IPI00654559	48.3	×	×	Smad interacting-protein 1
IPI00654696	41.5	×	×	synaptotagmin-like 2 isoform f
IPI00657839	162.2	×	×	hypothetical protein LOC54875
IPI00658151	395.9	×	×	similar to aortic preferentially expressed gene 1
IPI00658152	155.3	×	×	tensin-like SH2 domain containing 1
IPI00719051	82.2	×	×	Exocyst complex component 5
IPI00719442	89	×	×	Partial cDNA sequence, clone x529, unknown open reading frame
IPI00719621	212.7	×	×	Isoform 1 of Plexin-A2 precursor
IPI00719690	46.9	×	×	UEF3
IPI00719804	13.2	×	×	Similar to mammalian retrotransposon derived 8b
IPI00737969	108.3	×	×	IMP dehydrogenase/GMP reductase family protein
IPI00738628	175.5	×	×	similar to Rap guanine nucleotide exchange factor 2 (Neural RAP guanine nucleotide exchange protein) (nRap GEP) (PDZ domain-containing guanine nucleotide exchange factor 1) (PDZ-GEF1) (RA-GEF) isoform 9
IPI00738937	227.3	×	×	similar to CG17233-PA, isoform A isoform 2
IPI00739203	55.4	×	×	similar to nuclear localized factor 2
IPI00740166	82.3	×	×	Myosin 5B
IPI00744232	80.9	×	×	Isoform 2 of G protein-regulated inducer of neurite outgrowth 1
IPI00745214	83.4	×	×	Hypothetical protein FLJ13305 (Fragment)
IPI00748234	195.3	×	×	KIAA0423 protein
IPI00748284	67.7	×	×	NADPH-dependent FMN-and FAD-containing oxidoreductase
IPI00748877	112.6	×	×	coiled-coil domain containing 100
IPI00749222	22.3	×	×	22 kDa protein
IPI00759691	109.5	×	×	Isoform 2 of Bromodomain testis-specific protein

IPI00760639	11.4	×	×	11 kDa protein
IPI00761016	62.6	×	×	Hypothetical protein FLJ10925 (Fragment)
IPI00783879	296.9	×	×	human immunodeficiency virus type I enhancer binding protein 1
IPI00784002	504.6	×	×	sacsin
IPI00784545	24.8	×	×	Hypothetical protein
IPI00785023	256.7	×	×	Isoform 2 of Serine/arginine repetitive matrix protein 2
IPI00787428	76.7	×	×	similar to Protein C19orf16
IPI00000494	34.2	×	×	60S ribosomal protein L5
IPI00000870	17.7	×	×	Choriogonadotropin subunit beta precursor
IPI00001519	21.9	×	×	Protein SSX1
IPI00002333	33.1	×	×	CDNA FLJ43364 fis, clone NT2RP7017546
IPI00003411	57.1	×	×	Prostacyclin synthase
IPI00003774	40.2	×	×	dTDP-D-glucose 4,6-dehydratase
IPI00004367	10.5	×	×	FXYD domain-containing ion transport regulator 6 precursor
IPI00005510	28.7	×	×	Isoform GFRalpha4a of GDNF family receptor alpha-4 precursor
IPI00006146	13.5	×	×	serum amyloid A2
IPI00007289	59.5	×	×	Alkaline phosphatase, placental type precursor
IPI00007304	57.8	×	×	Protein ariadne-2 homolog
IPI00007614	48.3	×	×	Inward rectifier potassium channel 2
IPI00008600	42	×	×	Alpha-(1,3)-fucosyltransferase
IPI00009244	22.7	×	×	Adapter-related protein complex 1 sigma 1B subunit
IPI00009367	43	×	×	Serine--pyruvate aminotransferase
IPI00009949	29.8	×	×	Proteasome inhibitor PI31 subunit
IPI00010141	16.9	×	×	DNA polymerase epsilon subunit 3
IPI00010189	25.2	×	×	CDNA: FLJ22362 fis, clone HRC06544
IPI00010759	35.4	×	×	Homeobox protein Hox-C13
IPI00010862	56.9	×	×	Bone morphogenetic protein receptor type IB precursor
IPI00013914	119.1	×	×	V(D)J recombination-activating protein 1
IPI00014069	39.2	×	×	Protein Wnt-11 precursor
IPI00015160	26.2	×	×	CAP-binding protein complex interacting protein 1 (Fragment)

IPI00015361	17.3	×	×	Prefoldin subunit 5
IPI00016320	16.8	×	×	CDNA FLJ14126 fis, clone MAMMA1002571
IPI00016462	26.3	×	×	Protein C10orf95
IPI00016539	29	×	×	Hypothetical protein SCD4
IPI00016608	22.8	×	×	Transmembrane emp24 domain-containing protein 2 precursor
IPI00020968	47.1	×	×	Vasopressin V1a receptor
IPI00021578	37.3	×	×	Complement factor H-related protein 4 precursor
IPI00022208	16.4	×	×	CCAAT/enhancer-binding protein gamma
IPI00023043	10.5	×	×	HSPC054
IPI00023080	11.2	×	×	postsynaptic protein CRIPT
IPI00023343	90.3	×	×	Discs large homolog 3
IPI00023500	46.1	×	×	Isoform 1 of UDP-N-acetylglucosamine--dolichyl-phosphate N-acetylglucosaminephosphotransferase
IPI00024134	14.1	×	×	Ig kappa chain V-I region Walker precursor
IPI00025830	71.6	×	×	Wee1-like protein kinase
IPI00025854	64.8	×	×	similar to Alpha-1-antitrypsin-related protein precursor
IPI00026606	41.5	×	×	Surfeit locus protein 6
IPI00026663	56.1	×	×	Aldehyde dehydrogenase 1A3
IPI00027216	34	×	×	Taste receptor type 2 member 16
IPI00028480	20.4	×	×	Interleukin-19 precursor
IPI00031046	18	×	×	Hypothetical protein DKFZp434N035
IPI00034860	31.7	×	×	Isoform 3 of Coronin-6
IPI00043716	57.5	×	×	CDNA FLJ30696 fis, clone FCBBF2000808
IPI00045224	35	×	×	Isoform 6 of WW domain-containing oxidoreductase
IPI00045660	14.1	×	×	Novel protein
IPI00061087	30.5	×	×	Protein FAM122A
IPI00064771	42.6	×	×	ATP-sensitive inward rectifier potassium channel 15
IPI00065312	116.5	×	×	N-acetyl-beta-glucosaminyl-glycoprotein 4-beta-N-acetylgalactosaminyltransferase 1
IPI00099521	33.9	×	×	G4 protein

IPI00154778	21.6	×	×	Isoform 1 of Muted protein homolog
IPI00167951	24.4	×	×	PLTP9389
IPI00168153	17.2	×	×	CDNA FLJ34651 fis, clone KIDNE2018167
IPI00168223	14.4	×	×	CDNA FLJ33442 fis, clone BRACE2021936
IPI00168972	35.4	×	×	Olfactory receptor 4F6
IPI00175201	48.8	×	×	Zinc finger protein 645
IPI00178750	28.9	×	×	NEFA-interacting nuclear protein NIP30
IPI00178946	23.2	×	×	Isoform 1 of Nicotinamide riboside kinase 1
IPI00180681	82.9	×	×	NY-REN-58 antigen
IPI00184544	98.2	×	×	Zinc finger protein 484
IPI00186984	25	×	×	Isoform 4 of FRAS1-related extracellular matrix protein 1 precursor
IPI00216656	25.9	×	×	Isoform 2 of Putative potassium channel regulatory protein
IPI00216920	26.3	×	×	OTTHUMP00000030235
IPI00217553	15.4	×	×	mitochondrial ribosomal protein L41
IPI00218054	13.4	×	×	Selenoprotein H
IPI00220808	54.4	×	×	Isoform 2 of 6-phosphofructo-2-kinase/fructose-2,6-biphosphatase 2
IPI00221003	13	×	×	Nuclear transport factor 2-like export factor 2
IPI00234368	38.7	×	×	Isoform Short of Adenosine kinase
IPI00238891	46.5	×	×	Isoform C8 of CMP-N-acetylneuraminate-beta-1,4-galactoside alpha-2,3- sialyltransferase
IPI00244479	13.1	×	×	similar to Coiled-coil-helix-coiled-coil-helix domain-containing protein 2
IPI00256752	66.9	×	×	Isoform 3 of Epidermal growth factor receptor kinase substrate 8-like protein 3
IPI00288993	25.8	×	×	Transmembrane gamma-carboxyglutamic acid protein 3 precursor
IPI00294158	33.4	×	×	Biliverdin reductase A precursor
IPI00295772	57.3	×	×	cytochrome P450, family 51
IPI00301255	51.8	×	×	Immunoglobulin superfamily member 21 precursor

IPI00302552	54.9	×	×	Isoform 1 of Activin receptor type 1C precursor
IPI00303431	35.2	×	×	Olfactory receptor OR11-74
IPI00303615	35.3	×	×	Olfactory receptor 1L4
IPI00304664	39.4	×	×	KIAA1704 protein (Fragment)
IPI00329230	39.1	×	×	Isoform 1 of Protein FAM26A
IPI00329306	59.4	×	×	Keratin 5c
IPI00374975	28.8	×	×	Probable phosphoglycerate mutase 4
IPI00375582	55.8	×	×	Monocarboxylate transporter 9
IPI00382395	21.1	×	×	hypothetical protein LOC92806
IPI00382481	12.4	×	×	Ig heavy chain V-III region BUT
IPI00382488	13.6	×	×	Ig heavy chain V-III region HIL
IPI00382499	12.6	×	×	Ig heavy chain V-III region JON
IPI00382894	10.9	×	×	Tropomyosin 3
IPI00385717	28.5	×	×	Hypothetical protein DKFZp434L1713 (Fragment)
IPI00385799	23.4	×	×	erv1-like growth factor
IPI00386142	16.2	×	×	Ig heavy chain V-II region ARH-77 precursor
IPI00386308	32.2	×	×	FLJ00085 protein (Fragment)
IPI00394818	88.4	×	×	Isoform 1 of Ankyrin repeat domain-containing protein 47
IPI00394849	42.7	×	×	Progestin and adipoQ receptor family member 9
IPI00400895	22.6	×	×	KIAA1644 protein
IPI00400950	198.4	×	×	similar to RAN binding protein 2 isoform 7
IPI00401759	25.9	×	×	early estrogen-induced gene 1 protein isoform b
IPI00418983	164.1	×	×	sarcoma antigen NY-SAR-41
IPI00455971	37.9	×	×	38 kDa protein
IPI00464952	53.5	×	×	Splicing factor arginine/serine-rich 11
IPI00470901	215.3	×	×	dedicator of cytokinesis 5
IPI00554648	53.6	×	×	Keratin, type II cytoskeletal 8
IPI00645702	65.7	×	×	CTP synthase 2
IPI00719405	5	×	×	TMSL6 protein
IPI00745619	10.4	×	×	Similar to 37LRP/p40

IPI00784739	115	×	×	Uncharacterized protein C14orf43

abbreviations: TheoMW(theoretical Molecular weight), ExpMW_pla(experimental molecular weight in plasma), ExpMW_uri(exprimental molecular weight in urine),f(fragments), ptm(post-translational modification)
