# Supplementary material for: An Attempt to Understand Kidney's Protein Handling Function by Comparing Plasma and Urine Proteomes
Source: PLoS One. 2009 Apr 20;4(4):e5146. doi: 10.1371/journal.pone.0005146 (PMC2668176; doi:10.1371/journal.pone.0005146)
Supplement: Table S2 — Plasma-and-Urine subproteome (0.22 MB PDF) [file pone.0005146.s002.pdf]

Table S2 Plasma-and-Urine subproteome

IPI3.24	TheoMW(kD)	ExpMW_pla(kD)	ExpMW_uri(kD)	(Exp_pla-TheoMW)/TheoMW	Protein Name
IPI00006644	214.4	132.5	×	-38.20%	Isoform 2 of Plexin-B1 precursor
IPI00006662	21.3	23.7	×	11.27%	Apolipoprotein D precursor
IPI00009793	53.5	65.1	×	21.68%	Complement component 1, r subcomponent-like variant
IPI00009920	105.8	113.1	×	6.90%	Complement component C6 precursor
IPI00011261	22.3	15.7	×	-29.60%	Complement component C8 gamma chain precursor
IPI00017696	76.7	23.7	×	-69.10%	Complement C1s subcomponent precursor
IPI00019399	14.8	8.4	×	-43.24%	Serum amyloid A-4 protein precursor
IPI00019502	226.4	8.4	×	-96.29%	Myosin-9
IPI00019580	90.6	91.1	×	0.55%	Plasminogen precursor
IPI00019581	67.8	80	×	17.99%	Coagulation factor XII precursor
IPI00019591	85.5	91.1	×	6.55%	Isoform 1 of Complement factor B precursor (Fragment)
IPI00019943	69.1	80	×	15.77%	Afamin precursor
IPI00020091	23.6	38.8	×	64.41%	Alpha-1-acid glycoprotein 2 precursor
IPI00020986	38.4	65.1	×	69.53%	Lumican precursor
IPI00021856	11.3	6	×	-46.90%	Apolipoprotein C-II precursor
IPI00022395	63.2	58.7	×	-7.12%	Complement component C9 precursor
IPI00023019	43.8	38.8	×	-11.42%	Isoform 1 of Sex hormone-binding globulin precursor
IPI00023728	36	38.8	×	7.78%	Gamma-glutamyl hydrolase precursor
IPI00024825	151.1	136	×	-9.99%	Isoform A of Proteoglycan-4 precursor
IPI00025204	38.1	38.8	×	1.84%	CD5 antigen-like precursor
IPI00025864	72.9	97.2	×	33.33%	Cholinesterase precursor
IPI00026199	25.5	15.7	×	-38.43%	Glutathione peroxidase 3 precursor
IPI00027482	45.1	51.6	×	14.41%	Corticosteroid-binding globulin precursor
IPI00027509	78.4	91.1	×	16.20%	Matrix metalloproteinase-9 precursor

IPI00027843	44.7	58.7	×	31.32%	Isoform 1 of Vitamin K-dependent protein Z precursor
IPI00029193	70.7	91.2	×	29.00%	Hepatocyte growth factor activator precursor
IPI00029863	55.1	65.7	×	19.24%	Alpha-2-antiplasmin precursor
IPI00030205	14.1	17.4	×	23.40%	Ig kappa chain V-III region HAH precursor
IPI00030739	21.3	19.8	×	-7.04%	Apolipoprotein M
IPI00032291	188.3	65.1	×	-65.43%	Complement C5 precursor
IPI00154742	24.8	23.7	×	-4.44%	25 kDa protein
IPI00218192	101.2	136.1	×	34.49%	Isoform 2 of Inter-alpha-trypsin inhibitor heavy chain H4 precursor
IPI00218413	61.1	74.1	×	21.28%	biotinidase precursor
IPI00291867	65.7	35	×	-46.73%	Complement factor I precursor
IPI00292950	60.2	78.2	×	29.90%	Heparin cofactor 2 precursor
IPI00294395	67	58.7	×	-12.39%	Complement component C8 beta chain precursor
IPI00296099	129.4	19.8	×	-84.70%	Thrombospondin-1 precursor
IPI00296165	80.2	86.5	×	7.86%	Complement C1r subcomponent precursor
IPI00296608	93.5	91.1	×	-2.57%	Complement component C7 precursor
IPI00298971	54.3	58.7	×	8.10%	Vitronectin precursor
IPI00299435	35.4	27.8	×	-21.47%	apolipoprotein F precursor
IPI00299738	48	42.5	×	-11.46%	Procollagen C-endopeptidase enhancer 1 precursor
IPI00305461	106.4	132.3	×	24.34%	Inter-alpha-trypsin inhibitor heavy chain H2 precursor
IPI00384280	56.6	51.6	×	-8.83%	Prenylcysteine oxidase precursor
IPI00387025	11.7	19.8	×	69.23%	Ig kappa chain V-I region DEE
IPI00430847	25.7	50.7	×	97.28%	IGKC protein
IPI00431645	31.4	27.1	×	-13.69%	HP protein
IPI00448800	25.4	20.2	×	-20.47%	25 kDa protein
IPI00450768	48	74.7	×	55.63%	Keratin, type I cytoskeletal 17
IPI00453473	11.2	17.2	×	53.57%	Histone H4
IPI00479116	60.6	89.3	×	47.36%	Carboxypeptidase N subunit 2 precursor

IPI00549330	12.6	18.5	×	46.83%	Myosin-reactive immunoglobulin light chain variable region
IPI00550640	52	75.5	×	45.19%	IGHG4 protein
IPI00027350	21.9	15.7	×	-28.31%	Peroxiredoxin 2
IPI00021439	41.7	38.8	×	-6.95%	Actin, cytoplasmic 1
IPI00218918	38.7	42.5	×	9.82%	annexin I
IPI00019038	16.5	11.5	×	-30.30%	Lysozyme C precursor
IPI3.24	TheoMW(kD)	ExpMW_pla(kD)	ExpMW_uri(kD)	2(Exp_pla-Exp_uri)/(Exp_pla+Exp_uri)	Protein Name
IPI00006114	46.3	42.5	44	-3.47%	Pigment epithelium-derived factor precursor
IPI00009028	22.6	11.5	23	-66.67%	Tetranectin precursor
IPI00020996	66	80.1	77, ptm	3.95%	Insulin-like growth factor binding protein complex acid labile chain precursor
IPI00021842	36.2	28.4	f:11	88.32%	Apolipoprotein E precursor
IPI00022391	25.4	19.8	24	-19.18%	Serum amyloid P-component precursor
IPI00022417	38.2	42.5	42-46, ptm	-3.47%	Leucine-rich alpha-2-glycoprotein precursor
IPI00022420	23	15.7	23	-37.73%	Plasma retinol-binding protein precursor
IPI00022426	39	26.3	38	-36.39%	AMBP protein precursor [Contains: Alpha-1-microglobulin
IPI00026314	85.7	86.5	f:47	59.18%	Gelsolin precursor, plasma
IPI00178926	18.1	17.4	24, ptm	-31.88%	Hypothetical protein
IPI00291866	55.2	91.1	75	19.39%	Plasma protease C1 inhibitor precursor
IPI00292530	101.4	132.6	f:29	128.22%	Inter-alpha-trypsin inhibitor heavy chain H1 precursor
IPI00292946	46.3	51.6	f:15	109.91%	Thyroxine-binding globulin precursor
IPI00294193	103.3	102.4	34-37	97.03%	Splice isoform 1 of Q14624
IPI00304273	45.4	38.8	45	-14.80%	Apolipoprotein A-IV precursor
IPI00387120	12.6	19.8	23-27, ptm	-23.21%	Ig kappa chain V-IV region Len
IPI3.24	TheoMW(kD)	ExpMW_pla(kD)	ExpMW_uri(kD)	(Exp_uri-TheoMW)/TheoMW	Protein Name
IPI00007082	15.2	×	16	5.26%	Interleukin-5 precursor
IPI00009771	69.9	×	59	-15.59%	Lamin B2

IPI00011229	44.6	×	27	-39.46%	Cathepsin D precursor
IPI00013179	21	×	24－27, ptm	21.43%	Prostaglandin-H2 D-isomerase precursor
IPI00014375	109.2	×	f:19	-82.60%	Glutamyl aminopeptidase
IPI00018136	81.3	×	f:39	-52.03%	Isoform 1 of Vascular cell adhesion protein 1 precursor
IPI00019190	57	×	50	-12.28%	Myocilin precursor
IPI00021447	57.7	×	60	3.99%	Alpha-amylase 2B precursor
IPI00021885	95	×	f:40	-57.89%	Isoform 1 of Fibrinogen alpha chain precursor
IPI00024284	468.8	×	f:24	-94.88%	Basement membrane-specific heparan sulfate proteoglycan core protein precursor
IPI00025363	49.9	×	46	-7.82%	Isoform 1 of Glial fibrillary acidic protein, astrocyte
IPI00025861	97.5	×	82	-15.90%	Epithelial-cadherin precursor
IPI00026824	41.7	×	40	-4.08%	Heme oxygenase 2
IPI00027487	43.1	×	f:16	-62.88%	Creatine kinase M-type
IPI00028610	163.3	×	various f	×	Isoform 4 of Copper-transporting ATPase 1
IPI00028623	35.1	×	35	-0.28%	Estrogen sulfotransferase
IPI00029236	30.6	×	28	-8.50%	Insulin-like growth factor-binding protein 5 precursor
IPI00029658	54.6	×	46	-15.75%	Isoform 1 of EGF-containing fibulin-like extracellular matrix protein 1 precursor
IPI00032516	48.5	×	49	1.03%	AP-1 complex subunit mu-1
IPI00217975	66.3	×	58	-12.52%	Lamin-B1
IPI00219217	36.5	×	37	1.37%	L-lactate dehydrogenase B chain
IPI00219684	14.7	×	15	2.04%	Fatty acid-binding protein, heart
IPI00220361	29.9	×	26	-13.04%	Calbindin
IPI00292134	98.7	×	70	-29.08%	Epidermal growth factor receptor substrate 15
IPI00294386	118.4	×	f:52	-56.08%	Myosin Ia
IPI00294713	75.7	×	20	-73.58%	Isoform 1 of Mannan-binding lectin serine protease 2 precursor

IPI00307162	123.8	×	f:60	-51.53%	vinculin isoform meta-VCL
IPI00328609	48.5	×	50	3.09%	Kallistatin precursor
IPI00374563	214.8	×	f:23	-89.29%	Agrin precursor
IPI00387022	12	×	23－27, ptm	108.33%	Ig kappa chain V-I region AG
IPI00400909	72.4	×	78	7.73%	Isoform 1 of Rho-GTPase-activating protein 25
IPI00472345	57.2	×	45－50, ptm	-16.96%	IGHG3 protein
IPI00477597	39	×	39	0.00%	Isoform 1 of Haptoglobin-related protein precursor
IPI00550731	26.2	×	20－23, ptm	58.40%	Hypothetical protein
IPI00024095	36.4	×	40	9.89%	Annexin A3
IPI3.24	TheoMW(kD)	ExpMW_pla(kD)	ExpMW_uri(kD)		Protein Name
IPI00000045	20.1	×	×	×	Isoform 1 of Interleukin-1 receptor antagonist protein precursor
IPI00000220	15	×	×	×	hypothetical protein
IPI00001610	17	×	×	×	Insulin-like growth factor IA precursor
IPI00001674	13.4	×	×	×	Isoform 1 of Neurokinin-B precursor
IPI00002459	75.3	×	×	×	annexin VI isoform 2
IPI00002525	18.9	×	×	×	Neudesin precursor
IPI00002745	33.9	×	×	×	Cathepsin Z precursor
IPI00002856	31.8	×	×	×	CDNA: FLJ23153 fis, clone LNG09441
IPI00003437	12.1	×	×	×	Guanylate cyclase activator 2B precursor
IPI00003469	11.8	×	×	×	Ig kappa chain V-I region WEA
IPI00003590	82.6	×	×	×	Isoform 1 of Sulfhydryl oxidase 1 precursor
IPI00004101	45	×	×	×	Betaine--homocysteine S-methyltransferase
IPI00004310	36	×	×	×	Ly6/PLAUR domain-containing protein 3 precursor
IPI00004358	96.6	×	×	×	Glycogen phosphorylase, brain form
IPI00004372	84.4	×	×	×	Meprin A subunit alpha precursor
IPI00004503	44.9	×	×	×	lysosomal-associated membrane protein 1
IPI00004656	13.7	×	×	×	Beta-2-microglobulin precursor
IPI00004798	27.6	×	×	×	Cysteine-rich secretory protein 3 precursor

IPI00005707	166.7	×	×	×	Macrophage mannose receptor 2 precursor
IPI00005721	10.2	×	×	×	Neutrophil defensin 1 precursor
IPI00005794	59.9	×	×	×	Blood plasma glutamate carboxypeptidase precursor
IPI00006173	54.8	×	×	×	Cholesteryl ester transfer protein precursor
IPI00006288	169.9	×	×	×	Isoform 1 of Slit homolog 2 protein precursor
IPI00006556	88.7	×	×	×	hypothetical protein LOC9865
IPI00007257	108.6	×	×	×	calsyntenin 1 isoform 2
IPI00007726	30.6	×	×	×	Kallikrein-13 precursor
IPI00007778	43.8	×	×	×	Di-N-acetylchitobiase precursor
IPI00007798	117	×	×	×	Thyrotropin-releasing hormone-degrading ectoenzyme
IPI00007814	43.8	×	×	×	Vacuolar ATP synthase subunit C
IPI00007852	11.6	×	×	×	Myosin-reactive immunoglobulin light chain variable region (Fragment)
IPI00008494	57.8	×	×	×	Intercellular adhesion molecule 1 precursor
IPI00008669	38.5	×	×	×	38 kDa protein
IPI00008787	82.2	×	×	×	Alpha-N-acetylglucosaminidase precursor
IPI00008832	35.7	×	×	×	Growth-arrest-specific protein 1 precursor
IPI00009030	45	×	×	×	Isoform LAMP-2A of Lysosome-associated membrane glycoprotein 2 precursor
IPI00009342	189.3	×	×	×	Ras GTPase-activating-like protein IQGAP1
IPI00009477	30.7	×	×	×	Intercellular adhesion molecule 2 precursor
IPI00009890	44	×	×	×	Glia-derived nexin precursor
IPI00010130	41.9	×	×	×	Glutamine synthetase
IPI00010271	21.5	×	×	×	Isoform A of Ras-related C3 botulinum toxin substrate 1 precursor
IPI00010303	44.9	×	×	×	Serpin B4
IPI00010706	52.4	×	×	×	Glutathione synthetase
IPI00010863	7.4	×	×	×	Copper transport protein ATOX1

IPI00011155	35.5	×	×	×	Isoform 1 of Asialoglycoprotein receptor 2
IPI00011218	108	×	×	×	Macrophage colony-stimulating factor 1 receptor precursor
IPI00011255	69	×	×	×	Platelet glycoprotein Ib alpha chain precursor
IPI00011264	37.7	×	×	×	Complement factor H-related protein 1 precursor
IPI00011642	215.1	×	×	×	Isoform 1 of Receptor-type tyrosine-protein phosphatase delta precursor
IPI00011651	162.1	×	×	×	Receptor-type tyrosine-protein phosphatase gamma precursor
IPI00011694	26.6	×	×	×	Trypsin-1 precursor
IPI00012269	138.2	×	×	×	Multimerin-1 precursor
IPI00012440	54.1	×	×	×	Plasma alpha-L-fucosidase precursor
IPI00013508	103.1	×	×	×	Alpha-actinin-1
IPI00013569	198.5	×	×	×	Isoform 1 of Pappalysin-2 precursor
IPI00013808	104.9	×	×	×	Alpha-actinin-4
IPI00013880	120.6	×	×	×	Semaphorin-5A precursor
IPI00013885	27.7	×	×	×	Caspase-14 precursor
IPI00013933	331.8	×	×	×	Isoform DPI of Desmoplakin
IPI00014055	45.4	×	×	×	Napsin-A precursor
IPI00014439	25.8	×	×	×	Dihydropteridine reductase
IPI00015525	104.4	×	×	×	Multimerin-2 precursor
IPI00015756	162.1	×	×	×	Receptor-type tyrosine-protein phosphatase kappa precursor
IPI00016334	71.6	×	×	×	Isoform 1 of Cell surface glycoprotein MUC18 precursor
IPI00016373	22.8	×	×	×	Ras-related protein Rab-13
IPI00016450	74.3	×	×	×	Amiloride-sensitive sodium channel gamma-subunit
IPI00016862	56.3	×	×	×	Isoform Mitochondrial of Glutathione reductase, mitochondrial precursor

IPI00016915	29.1	×	×	×	Insulin-like growth factor-binding protein 7 precursor
IPI00017160	33.9	×	×	×	Protein C6orf55
IPI00017184	60.6	×	×	×	EH-domain-containing protein 1
IPI00017530	34	×	×	×	Ficolin-2 precursor
IPI00017567	70.6	×	×	×	Isoform Long of Endoglin precursor
IPI00017855	85.4	×	×	×	Aconitate hydratase, mitochondrial precursor
IPI00017968	20.4	×	×	×	ADM precursor
IPI00018305	31.7	×	×	×	Insulin-like growth factor-binding protein 3 precursor
IPI00018342	21.6	×	×	×	Adenylate kinase isoenzyme 1
IPI00018769	130	×	×	×	Thrombospondin-2 precursor
IPI00019157	250.5	×	×	×	Chondroitin sulfate proteoglycan 4 precursor
IPI00019568	70	×	×	×	Prothrombin precursor (Fragment)
IPI00019579	27	×	×	×	Complement factor D precursor
IPI00019755	27.6	×	×	×	Glutathione transferase omega-1
IPI00020407	84.5	×	×	×	Alpha-1,6-mannosylglycoprotein 6-beta-N-acetylglucosaminyltransferase V
IPI00020557	504.6	×	×	×	Low-density lipoprotein receptor-related protein 1 precursor
IPI00021033	138.6	×	×	×	Collagen alpha-1(III) chain precursor
IPI00021034	160.6	×	×	×	Collagen alpha-1(IV) chain precursor
IPI00021405	74.1	×	×	×	Isoform A of Lamin-A/C
IPI00021812	312.5	×	×	×	Neuroblast differentiation-associated protein AHNAK (Fragment)
IPI00021828	11.1	×	×	×	Cystatin B
IPI00021834	35	×	×	×	Isoform Alpha of Tissue factor pathway inhibitor precursor
IPI00021855	9.3	×	×	×	Apolipoprotein C-I precursor

IPI00021891	51.5	×	×	×	Isoform Gamma-B of Fibrinogen gamma chain precursor
IPI00022204	44.6	×	×	×	Serpin B3
IPI00022314	24.7	×	×	×	Superoxide dismutase [Mn], mitochondrial precursor
IPI00022331	49.6	×	×	×	Phosphatidylcholine-sterol acyltransferase precursor
IPI00022434	71.7	×	×	×	ALB protein
IPI00022649	131.4	×	×	×	Isoform 1 of Solute carrier family 12 member 2
IPI00022774	89.2	×	×	×	Transitional endoplasmic reticulum ATPase
IPI00022822	153.8	×	×	×	Isoform Long of Collagen alpha-1(XVIII) chain precursor
IPI00022830	37.3	×	×	×	Isoform 2 of NSFL1 cofactor p47
IPI00022937	252.3	×	×	×	Coagulation factor V
IPI00022959	61	×	×	×	Isoform 1 of Poliovirus receptor-related protein 3 precursor
IPI00023014	309.3	×	×	×	von Willebrand factor precursor
IPI00023814	160	×	×	×	Isoform 1 of Neogenin precursor
IPI00024046	78.3	×	×	×	Cadherin-13 precursor
IPI00024175	27.9	×	×	×	Isoform 1 of Proteasome subunit alpha type 7
IPI00024292	521.9	×	×	×	Low-density lipoprotein receptor-related protein 2 precursor
IPI00024966	113.4	×	×	×	Contactin-2 precursor
IPI00025276	464.5	×	×	×	Isoform XB of Tenascin-X precursor
IPI00025418	295.2	×	×	×	Collagen alpha-1(VII) chain precursor
IPI00025465	33.9	×	×	×	Mimecan precursor
IPI00025753	113.7	×	×	×	Desmoglein-1 precursor
IPI00026154	59.4	×	×	×	Glucosidase 2 subunit beta precursor
IPI00026240	35.7	×	×	×	ADP-ribosyl cyclase 2 precursor
IPI00026781	273.4	×	×	×	Fatty acid synthase

IPI00026926	12.4	×	×	×	Guanylin precursor
IPI00026944	136.5	×	×	×	Isoform 1 of Nidogen-1 precursor
IPI00027087	140	×	×	×	Isoform 1 of Neural cell adhesion molecule L1 precursor
IPI00027310	254.6	×	×	×	Isoform 1 of Multiple epidermal growth factor-like domains 8
IPI00027410	61	×	×	×	Platelet glycoprotein V precursor
IPI00027481	141.5	×	×	×	Multidrug resistance protein 1
IPI00027493	57.9	×	×	×	4F2 cell-surface antigen heavy chain
IPI00027497	63	×	×	×	Glucose-6-phosphate isomerase
IPI00027547	11.3	×	×	×	Dermcidin precursor
IPI00027780	73.9	×	×	×	72 kDa type IV collagenase precursor
IPI00027848	166	×	×	×	Macrophage mannose receptor 1 precursor
IPI00028030	82.8	×	×	×	Cartilage oligomeric matrix protein precursor
IPI00028553	34.7	×	×	×	Isoform 2 of Multiple inositol polyphosphate phosphatase 1 precursor
IPI00028911	97.6	×	×	×	Dystroglycan precursor
IPI00028931	122.3	×	×	×	desmoglein 2 preproprotein
IPI00029168	501.3	×	×	×	Apolipoprotein
IPI00029235	25.3	×	×	×	Insulin-like growth factor-binding protein 6 precursor
IPI00029699	16.8	×	×	×	Ribonuclease 4 precursor
IPI00029715	147.9	×	×	×	Aldehyde oxidase
IPI00029751	113.3	×	×	×	Isoform 1 of Contactin-1 precursor
IPI00029819	243.7	×	×	×	Neurogenic locus notch homolog protein 3 precursor
IPI00030385	26	×	×	×	CDNA FLJ13813 fis, clone THYRO1000358, moderately similar to SELENIUM- BINDING LIVER PROTEIN
IPI00030871	57	×	×	×	Pantetheinase precursor

IPI00031549	100	×	×	×	Isoform 3A of Desmocollin-3 precursor
IPI00031708	46.4	×	×	×	Fumarylacetoacetase
IPI00031789	65.4	×	×	×	Isoform 1 of Interleukin-1 receptor accessory protein precursor
IPI00032532	74.9	×	×	×	Isoform 2 of Growth-arrest-specific protein 6 precursor
IPI00032561	39.9	×	×	×	Calcium-binding protein 39
IPI00033600	41.6	×	×	×	Isoform 1 of Protein phosphatase 1 regulatory subunit 7
IPI00044369	59.6	×	×	×	Isoform 1 of Plexin domain-containing protein 2 precursor
IPI00045512	613.5	×	×	×	hemicentin 1
IPI00062266	46.6	×	×	×	Secernin-2
IPI00064262	346.2	×	×	×	Protocadherin-16 precursor
IPI00065501	36.3	×	×	×	CDNA FLJ32416 fis, clone SKMUS2000774, weakly similar to BALBIANI RING PROTEIN 1
IPI00100160	136.4	×	×	×	Isoform 1 of Cullin-associated NEDD8-dissociated protein 1
IPI00104074	125.4	×	×	×	Isoform 1 of Scavenger receptor cysteine-rich type 1 protein M130 precursor
IPI00107831	211.8	×	×	×	Receptor-type tyrosine-protein phosphatase F precursor
IPI00152540	161.7	×	×	×	Isoform 1 of CD109 antigen precursor
IPI00152871	64.4	×	×	×	Leucine-rich repeat-containing protein 15 precursor
IPI00152881	216.9	×	×	×	Shroom-related protein
IPI00153049	49.6	×	×	×	Novel protein
IPI00160130	398.7	×	×	×	Cubilin precursor
IPI00161229	12.1	×	×	×	12 kDa protein
IPI00162735	141.4	×	×	×	Isoform 2 of Attractin precursor

IPI00163207	62.2	×	×	×	Isoform 1 of N-acetylmuramoyl-L-alanine amidase precursor
IPI00163459	24.1	×	×	×	CDNA FLJ23816 fis, clone HSI02685
IPI00164755	0	×	×	×	Pro-alpha-2(I) collagen N-prepropeptide precursor (Fragment)
IPI00165421	29.3	×	×	×	SERPINC1 protein
IPI00166866	53.4	×	×	×	IGHA1 protein
IPI00167710	47.4	×	×	×	Hypothetical protein FLJ37440
IPI00178352	291.3	×	×	×	Isoform 1 of Filamin-C
IPI00180707	351.2	×	×	×	Isoform 1 of FRAS1-related extracellular matrix protein 2 precursor
IPI00186826	102.6	×	×	×	Receptor protein tyrosine kinase variant EphB4v1
IPI00215894	47.9	×	×	×	Isoform LMW of Kininogen-1 precursor
IPI00216298	11.6	×	×	×	Thioredoxin
IPI00216677	91.4	×	×	×	Cadherin-3 precursor
IPI00216694	70.8	×	×	×	plastin 3
IPI00216728	153.9	×	×	×	Neurexin 3-alpha
IPI00217882	92.1	×	×	×	Sortilin precursor
IPI00217966	36.7	×	×	×	lactate dehydrogenase A
IPI00218407	39.3	×	×	×	Fructose-bisphosphate aldolase B
IPI00218803	77.2	×	×	×	Isoform B of Fibulin-1 precursor
IPI00218914	54.7	×	×	×	Retinal dehydrogenase 1
IPI00219131	33.3	×	×	×	Isoform 1 of ICOS ligand precursor
IPI00219221	14.9	×	×	×	Galectin-7
IPI00219226	80.2	×	×	×	Sarcoplasmic reticulum histidine-rich calcium-binding protein precursor
IPI00219575	52.6	×	×	×	Bleomycin hydrolase
IPI00219757	23.2	×	×	×	Glutathione S-transferase P
IPI00219806	11.3	×	×	×	Protein S100-A7

IPI00220249	173.5	×	×	×	Latent-transforming growth factor beta-binding protein, isoform 1L precursor
IPI00220327	65.9	×	×	×	Keratin, type II cytoskeletal 1
IPI00220701	321.4	×	×	×	Isoform 2 of Collagen alpha-3(VI) chain precursor
IPI00221020	43.9	×	×	×	Isoform EP3E of Prostaglandin E2 receptor, EP3 subtype
IPI00221246	115.5	×	×	×	115 kDa protein
IPI00239117	371.7	×	×	×	Isoform 2 of Polycystic kidney and hepatic disease 1 precursor
IPI00246058	96.8	×	×	×	PDCD6IP protein
IPI00246975	26.4	×	×	×	Glutathione S-transferase Mu 3
IPI00257882	54.4	×	×	×	Xaa-Pro dipeptidase
IPI00289334	278.2	×	×	×	Isoform 1 of Filamin-B
IPI00289819	274.3	×	×	×	Cation-independent mannose-6-phosphate receptor precursor
IPI00289831	217.1	×	×	×	Isoform PTPS of Receptor-type tyrosine-protein phosphatase S precursor
IPI00290085	99.8	×	×	×	Cadherin-2 precursor
IPI00290328	151.9	×	×	×	Receptor-type tyrosine-protein phosphatase eta precursor
IPI00290856	35.2	×	×	×	Lymphatic vessel endothelial hyaluronic acid receptor 1 precursor
IPI00291175	116.7	×	×	×	vinculin isoform VCL
IPI00291560	34.7	×	×	×	Isoform 1 of Arginase-1
IPI00291641	73	×	×	×	Mannosyl-oligosaccharide 1,2-alpha-mannosidase IA
IPI00292150	195.1	×	×	×	Latent-transforming growth factor beta-binding protein 2 precursor
IPI00292218	80.4	×	×	×	Hepatocyte growth factor-like protein precursor

IPI00292579	277	×	×	×	Stabilin-2 precursor
IPI00293530	53.9	×	×	×	C3a anaphylatoxin chemotactic receptor
IPI00293748	55.1	×	×	×	Isoform 1 of Multiple inositol polyphosphate phosphatase 1 precursor
IPI00293849	163.6	×	×	×	Receptor-type tyrosine-protein phosphatase mu precursor
IPI00296180	48.5	×	×	×	Urokinase-type plasminogen activator precursor
IPI00296191	55.9	×	×	×	Isoform 1 of Vacuolar ATP synthase subunit H
IPI00296537	74.5	×	×	×	Isoform C of Fibulin-1 precursor
IPI00296654	49.2	×	×	×	Bactericidal/permeability-increasing protein-like 1 precursor
IPI00297026	45.1	×	×	×	Inhibin beta B chain precursor
IPI00297284	35.1	×	×	×	Insulin-like growth factor-binding protein 2 precursor
IPI00298281	177.6	×	×	×	Laminin gamma-1 chain precursor
IPI00298994	271.2	×	×	×	271 kDa protein
IPI00299059	136.7	×	×	×	Isoform 2 of Neural cell adhesion molecule L1-like protein precursor
IPI00299547	22.6	×	×	×	Neutrophil gelatinase-associated lipocalin precursor
IPI00300376	76.6	×	×	×	Protein-glutamine gamma-glutamyltransferase E precursor
IPI00300865	64	×	×	×	Novel protein
IPI00301143	49.5	×	×	×	protease inhibitor 16 precursor
IPI00305064	81.6	×	×	×	Isoform CD44 of CD44 antigen precursor
IPI00305719	52.4	×	×	×	selenium binding protein 1
IPI00306378	20.6	×	×	×	Isoform 2 of Mannan-binding lectin serine protease 2 precursor
IPI00328550	105.9	×	×	×	Thrombospondin-4 precursor
IPI00328745	49.1	×	×	×	Reticulon-4 receptor-like 1 precursor

IPI00328746	46.1	×	×	×	Reticulon-4 receptor-like 2 precursor
IPI00329352	134.4	×	×	×	Nodal modulator 1 precursor
IPI00373968	70.1	×	×	×	IMP dehydrogenase/GMP reductase family protein
IPI00374065	213.7	×	×	×	similar to melanoma inhibitory activity 3 isoform 1
IPI00376427	92.9	×	×	×	Neural cell adhesion molecule 2 precursor
IPI00382420	11.9	×	×	×	Ig lambda chain V-I region HA
IPI00382436	11.4	×	×	×	Ig lambda chain V-III region SH
IPI00382478	12.4	×	×	×	Ig heavy chain V-III region TIL
IPI00382606	75.6	×	×	×	Factor VII active site mutant immunoconjugate
IPI00383814	110.2	×	×	×	transmembrane protein 132A isoform a
IPI00384402	11.9	×	×	×	Myosin-reactive immunoglobulin kappa chain variable region (Fragment)
IPI00384404	7.5	×	×	×	7 kDa protein
IPI00384542	122.1	×	×	×	Isoform 2 of Nidogen-1 precursor
IPI00384697	47.4	×	×	×	Isoform 2 of Serum albumin precursor
IPI00384938	52.9	×	×	×	Hypothetical protein DKFZp686N02209
IPI00385555	11.8	×	×	×	Ig kappa chain V-I region BAN
IPI00385985	11.9	×	×	×	Ig lambda chain V-III region LOI
IPI00387026	11.8	×	×	×	Ig kappa chain V-I region EU
IPI00387100	11.8	×	×	×	Ig kappa chain V-I region Roy
IPI00387101	11.8	×	×	×	Ig kappa chain V-I region Scw
IPI00387110	12.1	×	×	×	Ig kappa chain V-II region MIL
IPI00387113	11.6	×	×	×	Ig kappa chain V-III region B6
IPI00395488	71.7	×	×	×	Vasorin precursor
IPI00397522	631.8	×	×	×	632 kDa protein
IPI00397801	248.1	×	×	×	Ifapsoriasin
IPI00398837	164.8	×	×	×	coiled-coil domain containing 88
IPI00411356	48.9	×	×	×	Vacuolar protein sorting-associating protein 4A
IPI00412492	212.1	×	×	×	Isoform 1 of Plexin-D1 precursor

IPI00413778	15.7	×	×	×	FKBP1A protein
IPI00414315	80.6	×	×	×	Isoform 1 of Epidermal growth factor receptor kinase substrate 8-like protein 2
IPI00419237	56.2	×	×	×	leucine aminopeptidase 3
IPI00419424	26.2	×	×	×	IGKV1-5 protein
IPI00419517	13	×	×	×	IGHV1-69 protein
IPI00442294	38	×	×	×	Isoform 1 of Neurotrimin precursor
IPI00465028	30.8	×	×	×	Triosephosphate isomerase 1 variant
IPI00465439	39.3	×	×	×	Fructose-bisphosphate aldolase A
IPI00470535	125.3	×	×	×	Dihydropyridine receptor alpha 2 subunit
IPI00472610	52.7	×	×	×	IGHM protein
IPI00478816	121	×	×	×	Serine protease inhibitor Kazal-type 5 precursor
IPI00479359	69.4	×	×	×	villin 2
IPI00554711	81.5	×	×	×	Junction plakoglobin
IPI00643920	67.9	×	×	×	Transketolase
IPI00647704	53.3	×	×	×	CDNA FLJ41552 fis, clone COLON2004478, highly similar to Protein Tro alpha1 H,myeloma
IPI00736507	13.4	×	×	×	Immunglobulin heavy chain variable region (Fragment)
IPI00739099	144.7	×	×	×	Collagen alpha-2(V) chain precursor
IPI00785200	25	×	×	×	Hypothetical protein
IPI00003362	72.4	×	×	×	Hypothetical protein
IPI00010154	50.6	×	×	×	Rab GDP dissociation inhibitor alpha
IPI00013890	27.8	×	×	×	Isoform 1 of 14-3-3 protein sigma
IPI00027452	55	×	×	×	Solute carrier family 2, facilitated glucose transporter member 5
IPI00169383	44.5	×	×	×	Phosphoglycerate kinase 1
IPI00304925	70	×	×	×	Heat shock 70 kDa protein 1
IPI00411706	31.5	×	×	×	S-formylglutathione hydrolase
IPI00419585	17.9	×	×	×	Peptidyl-prolyl cis-trans isomerase A

IPI00020008	9.1	×	×	×	NEDD8 precursor
IPI00027038	44	×	×	×	Isoform 1 of V-set and immunoglobulin domain-containing protein 4 precursor
IPI00029268	78.9	×	×	×	Neutral and basic amino acid transport protein rBAT
IPI00030364	21.7	×	×	×	Transcription initiation factor TFIID subunit 10
IPI00215917	20.5	×	×	×	ADP-ribosylation factor 3
IPI00008274	51.7	×	×	×	Adenylyl cyclase-associated protein 1
IPI00292657	35.9	×	×	×	NADP-dependent leukotriene B4 12-hydroxydehydrogenase
IPI00384391	13.2	×	×	×	Myosin-reactive immunoglobulin heavy chain variable region (Fragment)

abbreviations: TheoMW(theoretical Molecular weight), ExpMW_pla(experimental molecular weight in plasma),
