# Supplementary material for: An Attempt to Understand Kidney's Protein Handling Function by Comparing Plasma and Urine Proteomes
Source: PLoS One. 2009 Apr 20;4(4):e5146. doi: 10.1371/journal.pone.0005146 (PMC2668176; doi:10.1371/journal.pone.0005146)
Supplement: Table S3 — Urine-only subproteome (0.49 MB PDF) [file pone.0005146.s003.pdf]

Table S3 Urine-only subproteome

IPI3.24	TheoMW(kD)	ExpMW_uri(kD)	(Exp_uri-TheoMW)/TheoMW	Protein Name
IPI00000070	95.4	78	-18.24%	Low-density lipoprotein receptor precursor
IPI00000760	29.6	30	1.35%	NG,NG-dimethylarginine dimethylaminohydrolase 2
IPI00003562	27.2	23	-15.44%	Cofactor required for Sp1 transcriptional activation subunit 9
IPI00003807	48.3	48	-0.62%	Lysosomal acid phosphatase precursor
IPI00003831	134.2	f:29	-78.39%	Isoform XB of Plasma membrane calcium-transporting ATPase
IPI00003919	40.9	42	2.69%	Glutaminyl-peptide cyclotransferase precursor
IPI00004392	22.6	23	1.77%	Isoform p26 of 7,8-dihydro-8-oxoguanine triphosphatase
IPI00007155	25.4	33	29.92%	Vacuolar protein sorting-associated protein 28 homolog
IPI00008530	34.3	41	19.53%	60S acidic ribosomal protein P0
IPI00009027	18.7	20	6.95%	Lithostathine 1 alpha precursor
IPI00009236	20.5	20	-2.44%	caveolin 1
IPI00009276	30.7	37, ptm	20.52%	Endothelial protein C receptor precursor
IPI00009305	32.7	34	3.98%	Glucosamine-6-phosphate isomerase
IPI00009316	33.4	36	7.78%	Isoform A of Peptidyl-prolyl cis-trans isomerase E
IPI00009375	32.5	34	4.62%	3-hydroxyanthranilate 3,4-dioxygenase
IPI00009650	19.3	f:13	-32.64%	Lipocalin-1 precursor
IPI00009744	42.7	46	7.73%	Aldehyde dehydrogenase 3B2
IPI00009950	40.2	33	-17.91%	Vesicular integral-membrane protein VIP36 precursor
IPI00010290	15.1	14	-7.28%	FABP1 protein (Fragment)
IPI00010675	14.3	14	-2.10%	Trefoil factor 2 precursor
IPI00010796	57.1	58	1.58%	Protein disulfide-isomerase precursor
IPI00011302	14.2	16－20, ptm	26.76%	CD59 glycoprotein precursor
IPI00011857	61.5	f:20	-67.48%	Chromatin assembly factor 1 subunit B
IPI00012268	100.2	f:25	-75.05%	26S proteasome non-ATPase regulatory subunit 2
IPI00012284	44.1	45	2.04%	Isoform 1 of DNA excision repair protein ERCC-8
IPI00012450	35.1	34	-3.13%	N-myc-interactor

IPI00012503	58.1	f:11	-81.07%	Isoform Sap-mu-0 of Proactivator polypeptide precursor
IPI00013400	29.7	28	-5.72%	Matrilysin precursor
IPI00013698	44.6	f:14	-68.61%	Acid ceramidase precursor
IPI00013723	18.2	18	-1.10%	Peptidyl-prolyl cis-trans isomerase NIMA-interacting 1
IPI00013905	30.3	30	-0.99%	5'-AMP-activated protein kinase subunit beta-2
IPI00013945	69.8	90－110, ptm	43.27%	Isoform 1 of Uromodulin precursor
IPI00014198	31.8	32	0.63%	Exosome complex exonuclease RRP42
IPI00014537	37.1	40	7.82%	Isoform 1 of Calumenin precursor
IPI00014624	32.1	20	-37.69%	Adapter-related protein complex 3 sigma 1 subunit
IPI00015988	38.2	f:19	-50.26%	HLA class I histocompatibility antigen, alpha chain G precursor
IPI00016764	50.4	47	-6.75%	Tyrosine aminotransferase
IPI00017597	32	29	-9.38%	Isoform 1 of Microtubule-associated protein RP/EB family member 3
IPI00017672	32.6	35	7.36%	CDNA FLJ25678 fis, clone TST04067, highly similar to PURINE NUCLEOSIDE PHOSPHORYLASE
IPI00018195	43	45	4.65%	Mitogen-activated protein kinase 3
IPI00018236	20.8	21	0.96%	Ganglioside GM2 activator precursor
IPI00019171	40	38	-5.00%	SH3-containing GRB2-like protein 2
IPI00019385	20.2	19	-5.94%	Hypothetical protein SSR4 (Fragment)
IPI00020436	24.4	25	2.46%	Ras-related protein Rab-11B
IPI00020454	30.5	32	4.92%	Deoxycytidine kinase
IPI00021062	19.9	20	0.50%	Alpha crystallin A chain
IPI00021343	18.7	18	-3.74%	Isoform 1 of Interleukin-1 family member 9
IPI00021738	53.8	58	7.81%	Collagenase 3 precursor
IPI00021800	45.2	50	10.62%	Isoform Alpha of Caspase-1 precursor
IPI00023640	14.2	16	12.68%	Programmed cell death protein 5
IPI00023897	37.6	35	-6.91%	Heat shock factor 2-binding protein
IPI00024048	88.9	97	9.11%	Cadherin-15 precursor
IPI00024107	14.5	16	10.34%	Isoform 1 of Alpha-synuclein

IPI00024254	56	48	-14.29%	Interferon-induced protein with tetratricopeptide repeats
IPI00024273	96.1	various f	×	Isoform Long of Very low-density lipoprotein receptor precursor
IPI00025476	57.7	57－60, ptm	1.39%	Pancreatic alpha-amylase precursor
IPI00025829	56.3	f:29	-48.49%	Cytochrome P450 2B6
IPI00026128	33.2	34	2.41%	Syntaxin-11
IPI00027175	21.7	22	1.38%	Sorcin
IPI00027463	10.2	11	7.84%	Protein S100-A6
IPI00027510	61.1	60	-1.80%	Interleukin-2 receptor subunit beta precursor
IPI00027745	74.7	f:16	-78.58%	Isoform Long of Beta-glucuronidase precursor
IPI00029013	45.8	46	0.44%	Isoform 1 of Protein FAM53B
IPI00029753	54.7	50	-8.59%	Acetylcholine receptor protein subunit epsilon precursor
IPI00029997	27.5	28	1.82%	6-phosphogluconolactonase
IPI00030023	33.3	33	-0.90%	Histamine N-methyltransferase
IPI00030154	28.7	31	8.01%	Proteasome activator complex subunit 1
IPI00031065	31.4	36, ptm	14.65%	Deoxyribonuclease-1 precursor
IPI00031115	88.2	80	-9.30%	Golgin subfamily A member 1
IPI00032294	16.2	16	-1.23%	Cystatin S precursor
IPI00032904	14.3	13	-9.09%	Beta-synuclein
IPI00044761	75	78	4.00%	Pseudouridylate synthase 7 homolog
IPI00056478	65	f:17	-73.85%	Isoform 1 of Immunoglobulin superfamily member 8 precursor
IPI00073179	67.6	60	-11.24%	Vacuolar protein sorting-associated protein 33A
IPI00170635	27	f:18	-33.33%	Secreted and transmembrane protein 1 precursor
IPI00183968	32.9	32	-2.74%	tropomyosin 3 isoform 1
IPI00215928	19.7	20	1.52%	Centrin-2
IPI00216133	33.6	f:24	-28.57%	Bile salt sulfotransferase
IPI00216138	22.5	22	-2.22%	Transgelin
IPI00216317	84	82	-2.38%	Isoform 1 of Transcription factor SOX-5
IPI00217236	12.7	12	-5.51%	Tubulin-specific chaperone A
IPI00218131	10.4	11	5.77%	Protein S100-A12

IPI00219720	23	25	8.70%	Recoverin
IPI00219897	74.4	f:18	-75.81%	Isoform Short of Long-chain-fatty-acid--CoA ligase 4
IPI00219930	15.4	15	-2.60%	Cellular retinoic acid-binding protein 1
IPI00237671	61.4	55	-10.42%	Neurofilament triplet L protein
IPI00243742	21.8	21	-3.67%	Myosin light polypeptide 3
IPI00290279	40.5	41	1.23%	Isoform Long of Adenosine kinase
IPI00290566	60.3	47	-22.06%	T-complex protein 1 subunit alpha
IPI00290928	44	f:26	-40.91%	Guanine nucleotide-binding protein alpha-13 subunit
IPI00291136	108.5	100	-7.83%	Collagen alpha-1(VI) chain precursor
IPI00293307	48.1	46	-4.37%	Adipophilin
IPI00295741	37.8	25, ptm	-33.86%	Cathepsin B precursor
IPI00296141	54.3	54	-0.55%	Dipeptidyl-peptidase 2 precursor
IPI00299076	24.4	27	10.66%	Receptor-binding cancer antigen expressed on SiSo cells (Fragment)
IPI00300786	57.8	57－60, ptm	1.21%	Salivary alpha-amylase precursor
IPI00303954	16.7	12	-28.14%	cytochrome b5 outer mitochondrial membrane precursor
IPI00304227	88	80	-9.09%	Isoform 1 of Cadherin-11 precursor
IPI00306576	59.7	55	-7.87%	Arylsulfatase B precursor
IPI00334756	54.6	f:27	-50.55%	Isoform B of Caspase-10 precursor
IPI00383110	57.4	f:29	-49.48%	Cytochrome P450 3A3
IPI00385509	92.7	100－105, ptm	10.57%	Phosphatidylinositol-glycan-specific phospholipase D 2 precursor
IPI00387115	11.8	23－27, ptm	111.86%	Ig kappa chain V-III region SIE
IPI00394882	24.4	23	-5.74%	Isoform 1 of Ras-related protein Rab-15
IPI00413728	284.5	various f	×	Isoform 1 of Spectrin alpha chain, brain
IPI00414909	46.6	49	5.15%	Alpha-N-acetylgalactosaminidase precursor
IPI00418471	53.5	46	-14.02%	Vimentin
IPI00419531	88.5	61	-31.07%	Cleavage and polyadenylation specificity factor 100 kDa subunit
IPI00430808	25.6	23－27, ptm	-2.34%	Hypothetical protein

IPI00438923	38.7	40	3.36%	Quinone oxidoreductase-like 1
IPI00448925	60.1	45－50, ptm	-20.97%	IGHG1 protein
IPI00465165	55	53	-3.64%	Isoform Long of Cysteine sulfinic acid decarboxylase
IPI00465352	21	18	-14.29%	Calcyphosin
IPI00470779	61.9	38	-38.61%	Alpha-taxilin
IPI00549813	45.3	40	-11.70%	Protein arginine N-methyltransferase 8
IPI00554752	46.2	42	-9.09%	cAMP-dependent protein kinase type II-beta regulatory subunit
IPI00641229	36.5	55－58, ptm	54.79%	Ig alpha-2 chain C region
IPI00641737	46.7	17	-63.60%	Haptoglobin precursor
IPI00643583	10.9	13	19.27%	Roadblock-1
IPI00718819	24.7	25－28, ptm	7.29%	Hypothetical protein
IPI00744184	53.6	55	2.61%	Arylsulfatase A precursor
IPI00744692	37.5	38	1.33%	Transaldolase
IPI00746352	10.4	10	-3.85%	SH3 domain-binding glutamic acid-rich-like protein 3
IPI00783170	48.5	46	-5.15%	Protein kinase C and casein kinase substrate in neurons protein 3
IPI00783446	105.3	110－120, ptm	9.21%	Lysosomal alpha-glucosidase precursor
IPI00784090	59.5	51	-14.29%	T-complex protein 1 subunit theta
IPI00787181	37.7	55－58, ptm	49.87%	Ig alpha-1 chain C region
IPI00793318	47.9	50	4.38%	Aspartyl protease 3
IPI00023526	23.6	25	5.93%	RAB6A, member RAS oncogene family isoform b
IPI00329801	35.9	34	-5.29%	Annexin A5
IPI00022585	97.3	f:25	-74.31%	Isoform 1 of A kinase anchor protein 1, mitochondrial precursor
IPI00014964	17	×	×	Lymphocyte antigen Ly-6H precursor
IPI00015018	32.7	×	×	Inorganic pyrophosphatase
IPI00015148	20.8	×	×	Ras-related protein Rap-1b precursor
IPI00015199	25.4	×	×	T-cell antigen CD7 precursor
IPI00015688	61.7	×	×	Glypican-1 precursor

IPI00015689	68.4	×	×	Isoform 1 of Zinc transporter ZIP4 precursor
IPI00015881	60.1	×	×	Isoform 1 of Macrophage colony-stimulating factor 1 precursor
IPI00015902	124	×	×	Beta platelet-derived growth factor receptor precursor
IPI00016027	63.6	×	×	Frizzled-2 precursor
IPI00016150	46.4	×	×	Neuroserpin precursor
IPI00016179	11.5	×	×	Protein S100-A13
IPI00016255	63.3	×	×	hypothetical protein LOC79887
IPI00016339	23.5	×	×	Ras-related protein Rab-5C
IPI00016342	23.5	×	×	Ras-related protein Rab-7
IPI00016513	22.5	×	×	Ras-related protein Rab-10
IPI00016645	112.1	×	×	Isoform 1 of Ephrin type-A receptor 7 precursor
IPI00016786	21.3	×	×	Isoform 2 of Cell division control protein 42 homolog precursor
IPI00017202	30.4	×	×	Isoform 1 of Chondrolectin precursor
IPI00017342	21.3	×	×	Rho-related GTP-binding protein RhoG precursor
IPI00017344	23.7	×	×	Ras-related protein Rab-5B
IPI00017367	68.6	×	×	Radixin
IPI00017469	28	×	×	Sepiapterin reductase
IPI00017519	15	×	×	Potassium voltage-gated channel subfamily E member 1-like protein
IPI00017526	10.4	×	×	Protein S100-P
IPI00017529	28.1	×	×	Isoform 1 of Lymphocyte function-associated antigen 3 precursor
IPI00017557	39.8	×	×	Secreted frizzled-related protein 4 precursor
IPI00017704	15.8	×	×	Coactosin-like protein
IPI00017763	43	×	×	Nucleosome assembly protein 1-like 4
IPI00017987	9.9	×	×	Cornifin A
IPI00018206	47.5	×	×	Aspartate aminotransferase, mitochondrial precursor
IPI00018235	30.4	×	×	Peflin

IPI00018248	24.4	×	×	ER lumen protein retaining receptor 2
IPI00018272	30	×	×	Pyridoxine-5'-phosphate oxidase
IPI00018276	92.5	×	×	Type I transmembrane receptor precursor
IPI00018278	13.4	×	×	Histone H2AV
IPI00018279	172.1	×	×	Collagen alpha-3(V) chain precursor
IPI00018282	30.6	×	×	Isoform I of Tumor necrosis factor receptor superfamily member 5 precursor
IPI00018364	20.5	×	×	Ras-related protein Rap-2b precursor
IPI00018434	43.9	×	×	Isoform 1 of Tumor susceptibility gene 101 protein
IPI00018471	86.4	×	×	Smoothened homolog precursor
IPI00018871	21.5	×	×	ADP-ribosylation factor-like protein 8B
IPI00018880	50.5	×	×	Tumor necrosis factor receptor superfamily member 1A precursor
IPI00018909	14.3	×	×	trefoil factor 3 precursor
IPI00018920	50.3	×	×	Isoform B of Carcinoembryonic antigen-related cell adhesion molecule 1 precursor
IPI00019176	18.6	×	×	Retinoic acid receptor responder protein 2 precursor
IPI00019449	18.4	×	×	Nonsecretory ribonuclease precursor
IPI00019599	25.8	×	×	Isoform 1 of Ubiquitin-conjugating enzyme E2 variant 1
IPI00019600	16.2	×	×	Ubiquitin-conjugating enzyme E2 variant 2
IPI00019641	42	×	×	Pepsin A precursor
IPI00019771	42.2	×	×	Fractalkine precursor
IPI00019862	37.9	×	×	butyrophilin, subfamily 2, member A1 isoform 2 precursor
IPI00019869	11.1	×	×	Protein S100-A2
IPI00019906	29.2	×	×	Isoform 2 of Basigin precursor
IPI00019907	65.6	×	×	Glypican-3 precursor
IPI00019931	75.7	×	×	Isoform 1 of Amiloride-sensitive sodium channel subunit alpha
IPI00019954	16.5	×	×	Cystatin M precursor
IPI00019988	56.7	×	×	N-sulphoglucosamine sulphohydrolase precursor

IPI00020101	13.9	×	×	H2B histone family, member A
IPI00020430	52.6	×	×	53 kDa protein
IPI00020468	21.1	×	×	Selenoprotein S
IPI00020599	48.1	×	×	Calreticulin precursor
IPI00020632	50.8	×	×	ArgininosuccinAte synthetAse
IPI00020672	82.6	×	×	Isoform 1 of Dipeptidyl-peptidase 3
IPI00020687	8.5	×	×	Pancreatic secretory trypsin inhibitor precursor
IPI00020977	38.1	×	×	Isoform 1 of Connective tissue growth factor precursor
IPI00020982	85.3	×	×	Isoform 1 of Amiloride-sensitive amine oxidase [copper-containing] precursor
IPI00020990	49.5	×	×	Osteomodulin precursor
IPI00021085	21.7	×	×	Peptidoglycan recognition protein precursor
IPI00021275	117.5	×	×	Isoform 1 of Ephrin type-B receptor 2 precursor
IPI00021302	90.2	×	×	Sushi domain-containing protein 2 precursor
IPI00021347	17.9	×	×	Ubiquitin-conjugating enzyme E2 L3
IPI00021369	20.2	×	×	Alpha crystallin B chain
IPI00021382	45.4	×	×	Interleukin-1 receptor type II precursor
IPI00021421	34.2	×	×	Isoform 1 of Lysosomal thioesterase PPT2 precursor
IPI00021434	12.3	×	×	Elafin precursor
IPI00021536	15.9	×	×	Calmodulin-like protein 5
IPI00021715	161	×	×	Isoform 1 of Collagen alpha-5(IV) chain precursor
IPI00021735	22.6	×	×	dendritic cell-associated C-type lectin 1 isoform b
IPI00021766	130.1	×	×	Isoform 1 of Reticulon-4
IPI00021794	54.5	×	×	Lysosomal protective protein precursor
IPI00021807	59.7	×	×	Isoform Long of Glucosylceramidase precursor
IPI00021833	24	×	×	Isoform Long of Platelet-derived growth factor A chain precursor
IPI00021923	24.7	×	×	Protein FAM3C precursor
IPI00021983	78.4	×	×	Isoform 1 of Nicastrin precursor
IPI00021997	24.1	×	×	Protein CREG1 precursor

IPI00022039	36.9	×	×	Isoform 3 of SLAM family member 5 precursor
IPI00022052	26.8	×	×	WNT1-inducible signaling pathway protein 2 precursor
IPI00022203	39.8	×	×	Myeloid cell surface antigen CD33 precursor
IPI00022232	12	×	×	Colipase precursor
IPI00022246	26.9	×	×	Azurocidin precursor
IPI00022283	9.1	×	×	Trefoil factor 1 precursor
IPI00022284	27.7	×	×	Major prion protein precursor
IPI00022290	7.4	×	×	Beta-defensin 1 precursor
IPI00022359	37.2	×	×	Aquaporin-7
IPI00022380	15.1	×	×	Psoriasis susceptibility 1 candidate gene 2 protein precursor
IPI00022606	47.6	×	×	Isoform 1 of Proline-serine-threonine phosphatase-interacting protein 1
IPI00022608	248.4	×	×	Sortilin-related receptor precursor
IPI00022620	11.2	×	×	Secreted Ly-6/uPAR-related protein 1 precursor
IPI00022624	40.3	×	×	Retinoic acid-induced protein 3
IPI00022661	57.7	×	×	Isoform Delta of Poliovirus receptor-related protein 2 precursor
IPI00022792	28.6	×	×	Microfibril-associated glycoprotein 4 precursor
IPI00022810	51.8	×	×	Dipeptidyl-peptidase 1 precursor
IPI00022892	17.9	×	×	Thy-1 membrane glycoprotein precursor
IPI00023011	8.2	×	×	Submaxillary gland androgen-regulated protein 3 homolog B precursor
IPI00023119	43.1	×	×	Anti-Fas-induced apoptosis
IPI00023501	48.3	×	×	Isoform 1 of Tumor necrosis factor receptor superfamily member 1B precursor
IPI00023510	23.7	×	×	Ras-related protein Rab-5A
IPI00023648	46	×	×	immunoglobulin superfamily containing leucine-rich repeat
IPI00023824	126.5	×	×	Fibulin-2 precursor
IPI00023858	29.9	×	×	Fc-gamma receptor IIIb

IPI00023974	20.3	×	×	Pituitary tumor-transforming gene 1 protein-interacting protein precursor
IPI00024035	88.3	×	×	Isoform 1 of Cadherin-6 precursor
IPI00024083	40.1	×	×	Isoform 1 of N-acylethanolamine-hydrolyzing acid amidase precursor
IPI00024129	22.8	×	×	Peptidyl-prolyl cis-trans isomerase C
IPI00024307	38	×	×	Ephrin-B1 precursor
IPI00024331	30.4	×	×	Tumor necrosis factor receptor superfamily member 14 precursor
IPI00024403	60.1	×	×	Copine-3
IPI00024406	12.8	×	×	LAMA4 protein
IPI00024638	47	×	×	Ubiquitous mitochondrial creatine kinase variant
IPI00024648	58.4	×	×	Matrix extracellular phosphoglycoprotein precursor
IPI00024660	39.8	×	×	Isoform 1 of Protein KIAA0174
IPI00024664	95.8	×	×	Isoform Long of Ubiquitin carboxyl-terminal hydrolase 5
IPI00024689	28.4	×	×	Aquaporin-1
IPI00024880	110	×	×	Isoform 1 of Signal peptide, CUB and EGF-like domain-containing protein 2 precursor
IPI00024896	31.8	×	×	Probable isomerase MAWBP
IPI00024919	27.7	×	×	Thioredoxin-dependent peroxide reductase, mitochondrial precursor
IPI00024929	41.3	×	×	adipocyte-specific adhesion molecule
IPI00024933	17.8	×	×	60S ribosomal protein L12
IPI00025019	26.5	×	×	Proteasome subunit beta type 1 precursor
IPI00025084	28.3	×	×	Calpain small subunit 1
IPI00025110	68.1	×	×	Isoform 2 of Mesothelin precursor
IPI00025240	89.9	×	×	Isoform 1 of Cadherin-16 precursor
IPI00025252	56.8	×	×	Protein disulfide-isomerase A3 precursor
IPI00025277	21.9	×	×	Programmed cell death protein 6
IPI00025285	13.6	×	×	Vacuolar ATP synthase subunit G 1

IPI00025318	12.8	×	×	SH3 domain-binding glutamic acid-rich-like protein
IPI00025365	25.5	×	×	Isoform Long of Endothelin-3 precursor
IPI00025366	51.7	×	×	Citrate synthase, mitochondrial precursor
IPI00025394	53.9	×	×	Aromatic-L-amino-acid decarboxylase
IPI00025427	18.4	×	×	Eosinophil cationic protein precursor
IPI00025686	169.5	×	×	latent transforming growth factor beta binding protein 4 isoform b
IPI00025840	23.8	×	×	Isoform 1 of Ephrin-A1 precursor
IPI00025846	100	×	×	Isoform 2A of Desmocollin-2 precursor
IPI00025869	48.8	×	×	Alpha-galactosidase A precursor
IPI00025878	12.9	×	×	Motilin precursor
IPI00025974	25	×	×	Charged multivesicular body protein 4b
IPI00025992	9.4	×	×	Hepcidin precursor
IPI00026031	36.8	×	×	Protein C6orf72 precursor
IPI00026050	46.3	×	×	Ceroid-lipofuscinosis neuronal protein 5
IPI00026087	10.1	×	×	Barrier-to-autointegration factor
IPI00026104	61.9	×	×	Isoform Long of Iduronate 2-sulfatase precursor
IPI00026125	33.9	×	×	Deoxyribonuclease I-like 1 precursor
IPI00026182	32.8	×	×	F-actin capping protein alpha-2 subunit
IPI00026185	31.4	×	×	Isoform 1 of F-actin capping protein subunit beta
IPI00026216	103.3	×	×	Puromycin-sensitive aminopeptidase
IPI00026237	69.1	×	×	Myelin-associated glycoprotein precursor
IPI00026256	435.2	×	×	Filaggrin
IPI00026268	37.2	×	×	Guanine nucleotide-binding protein G(I)/G(S)/G(T) subunit beta 1
IPI00026270	50.5	×	×	Carboxypeptidase M precursor
IPI00026299	13.8	×	×	Isoform Glycophorin C of Glycophorin C
IPI00026303	29.1	×	×	protease inhibitor 15 preproprotein
IPI00026569	40.8	×	×	HLA class I histocompatibility antigen, A-1 alpha chain precursor

IPI00027009	55.7	×	×	Isoform 1 of Protein kinase C and casein kinase substrate in neurons protein 2
IPI00027144	27.6	×	×	Cytochrome b561
IPI00027165	61.8	×	×	Isoform R-type of Pyruvate kinase isozymes R/L
IPI00027166	24.4	×	×	Metalloproteinase inhibitor 2 precursor
IPI00027240	7.2	×	×	Guanine nucleotide-binding protein G(I)/G(S)/G(O) gamma-5 subunit precursor
IPI00027255	22.8	×	×	Myosin light polypeptide 6B
IPI00027264	31.5	×	×	Calretinin
IPI00027377	250.8	×	×	Isoform 1 of Aggrecan core protein precursor
IPI00027409	27.8	×	×	Myeloblastin precursor
IPI00027416	44.7	×	×	Gamma-butyrobetaine dioxygenase
IPI00027434	22	×	×	Rho-related GTP-binding protein RhoC precursor
IPI00027436	45.2	×	×	Tumor necrosis factor receptor superfamily member 16 precursor
IPI00027438	47.4	×	×	Flotillin-1
IPI00027466	35	×	×	Carbonic anhydrase 4 precursor
IPI00027486	76.8	×	×	Carcinoembryonic antigen-related cell adhesion molecule 5 precursor
IPI00027491	36.5	×	×	Isoform A of Low affinity immunoglobulin epsilon Fc receptor
IPI00027703	129.3	×	×	Isoform Long of Alpha-mannosidase IIx
IPI00027769	28.5	×	×	Leukocyte elastase precursor
IPI00027806	56.9	×	×	Cysteine-rich secretory protein LCCL domain-containing 1 precursor
IPI00027821	55.9	×	×	Isoform 1 of Cysteine-rich secretory protein LCCL domain-containing 2 precursor
IPI00027827	25.9	×	×	Extracellular superoxide dismutase [Cu-Zn] precursor
IPI00027851	60.7	×	×	Beta-hexosaminidase alpha chain precursor
IPI00027862	48.6	×	×	Isoform 1 of Probable serine protease HTRA3 precursor

IPI00027993	23.5	×	×	Ras-related protein Rab-25
IPI00028004	22.9	×	×	Proteasome subunit beta type 3
IPI00028006	22.8	×	×	Proteasome subunit beta type 2
IPI00028015	29.8	×	×	Isoform 2 of Leukocyte-associated immunoglobulin-like receptor 1 precursor
IPI00028082	106.5	×	×	Reversion-inducing cysteine-rich protein with Kazal motifs precursor
IPI00028091	47.2	×	×	Actin-like protein 3
IPI00028135	87	×	×	Cadherin-19 precursor
IPI00028136	45.8	×	×	Isoform 1 of CD209 antigen
IPI00028381	40.5	×	×	Isoform 1 of EGF-like domain-containing protein 9
IPI00028450	108.5	×	×	Isoform 1 of Sodium/calcium exchanger 1 precursor
IPI00028509	7.5	×	×	Guanine nucleotide-binding protein G(I)/G(S)/G(O) gamma-7 subunit precursor
IPI00028514	84.3	×	×	Isoform PSMA-1 of Glutamate carboxypeptidase 2
IPI00028910	56.6	×	×	Dihydropyrimidinase
IPI00029046	32.2	×	×	Protein KIAA0152 precursor
IPI00029111	73.9	×	×	DPYSL3 protein
IPI00029123	49.6	×	×	Isoform A of Endothelin B receptor precursor
IPI00029275	80.2	×	×	Isoform 1 of Melanotransferrin precursor
IPI00029510	13.3	×	×	Lymphocyte antigen Ly-6D precursor
IPI00029601	61.8	×	×	Src substrate cortactin
IPI00029605	58	×	×	N-acetylgalactosamine-6-sulfatase precursor
IPI00029623	27.4	×	×	Proteasome subunit alpha type 6
IPI00029625	41.7	×	×	Flotillin-2
IPI00029723	35	×	×	Follistatin-related protein 1 precursor
IPI00029733	36.8	×	×	Aldo-keto reductase family 1 member C1
IPI00029817	45.5	×	×	Sialidase-1 precursor
IPI00030075	50.2	×	×	Fibroleukin precursor
IPI00030255	84.8	×	×	Procollagen-lysine,2-oxoglutarate 5-dioxygenase 3

IPI00030431	62.8	×	×	Isoform 1 of Anthrax toxin receptor 1 precursor
IPI00030877	18	×	×	15 kDa selenoprotein isoform 1 precursor
IPI00030911	11.4	×	×	Vesicle-associated membrane protein 8
IPI00030936	26.3	×	×	Tetraspanin-1
IPI00030941	28	×	×	Tetraspanin-3
IPI00030968	21.6	×	×	Chromosome 9 open reading frame 142
IPI00031008	240.9	×	×	Isoform 1 of Tenascin precursor
IPI00031030	87	×	×	Isoform 1 of Amyloid-like protein 2 precursor
IPI00031091	26.9	×	×	EF-hand domain-containing protein 1
IPI00031121	63.7	×	×	Carboxypeptidase E precursor
IPI00031138	21	×	×	Protease-associated domain-containing protein of 21 kDa precursor
IPI00031171	51.5	×	×	Isoform 1 of Interleukin-6 receptor alpha chain precursor
IPI00031420	55	×	×	UDP-glucose 6-dehydrogenase
IPI00031523	35.7	×	×	Heat shock protein 86 (Fragment)
IPI00031564	21	×	×	Uncharacterized protein C7orf24
IPI00031616	21.6	×	×	hypothetical protein LOC84299
IPI00031655	20.7	×	×	Vacuolar protein sorting-associated protein 25
IPI00031801	40.1	×	×	Isoform 1 of DNA-binding protein A
IPI00031821	30.3	×	×	Integral membrane protein 2B
IPI00032103	48.5	×	×	Isoform Mitochondrial of Glycine amidinotransferase, mitochondrial precursor
IPI00032313	11.7	×	×	Protein S100-A4
IPI00032425	16.5	×	×	Receptor activity-modifying protein 3 precursor
IPI00032825	25.2	×	×	Transmembrane emp24 domain-containing protein 7 precursor
IPI00032826	41.3	×	×	Hsc70-interacting protein
IPI00034319	20.9	×	×	Isoform A of Protein CutA precursor
IPI00034558	58.7	×	×	Neurexin-3-beta precursor
IPI00037448	35.7	×	×	Glyoxylate reductase/hydroxypyruvate reductase
IPI00042295	17.3	×	×	LOC87769 protein

IPI00043992	55.5	×	×	CDNA FLJ14847 fis, clone PLACE1000401, weakly similar to POLIOVIRUS RECEPTOR
IPI00045957	49.1	×	×	CREB/ATF family transcription factor
IPI00054521	71.2	×	×	Frizzled-1 precursor
IPI00056309	8.8	×	×	Liver-expressed antimicrobial peptide 2 precursor
IPI00056357	18.8	×	×	Uncharacterized protein C19orf10 precursor
IPI00059185	14	×	×	CDNA FLJ25219 fis, clone STM00503
IPI00059187	28.9	×	×	Transmembrane protein 106A
IPI00059476	45.7	×	×	Dipeptidase 1 precursor
IPI00059928	30.7	×	×	Transmembrane and immunoglobulin domain containing 2
IPI00060200	37.8	×	×	Aldose 1-epimerase
IPI00060800	22.7	×	×	hypothetical protein LOC124220
IPI00061116	9	×	×	Small breast epithelial mucin precursor
IPI00062047	71.1	×	×	Kinesin-like protein KIF12
IPI00063025	35.2	×	×	Aspartoacylase-2
IPI00063048	51.4	×	×	ST6GAL2 protein
IPI00063827	22.3	×	×	Isoform 1 of Abhydrolase domain-containing protein 14B
IPI00064377	46.1	×	×	Tumor necrosis factor receptor superfamily member 19L precursor
IPI00065500	46.5	×	×	Novel protein
IPI00066856	13.1	×	×	Ly6/PLAUR domain-containing protein 2 precursor
IPI00067738	26	×	×	Isoform B of Protein FAM3B precursor
IPI00069058	67.3	×	×	VGF nerve growth factor inducible precursor
IPI00072044	35.1	×	×	Isoform 1 of Ester hydrolase C11orf54
IPI00073196	139.4	×	×	Isoform 1 of Latent-transforming growth factor beta-binding protein 3 precursor
IPI00073772	36.7	×	×	Fructose-1,6-bisphosphatase 1
IPI00082931	18.2	×	×	Small proline-rich protein 3
IPI00095891	111	×	×	Isoform XLas-1 of Guanine nucleotide-binding protein G(s) subunit alpha isoforms XLas

IPI00098827	16.3	×	×	Placental protein 25
IPI00099386	54.6	×	×	Isoform 1 of Tubulointerstitial nephritis antigen
IPI00099670	79.7	×	×	Carboxyl ester lipase
IPI00099883	48.2	×	×	G-protein coupled receptor family C group 5 member C precursor
IPI00100067	46	×	×	Isoform 1 of Tumor necrosis factor receptor superfamily member 19 precursor
IPI00100673	24.9	×	×	Charged multivesicular body protein 3
IPI00100796	24.6	×	×	Charged multivesicular body protein 5
IPI00101037	37.5	×	×	Reticulocalbin-3 precursor
IPI00101524	28.9	×	×	Isoform 1 of Vacuolar sorting protein SNF8
IPI00102300	67.3	×	×	Isoform 3 of Platelet glycoprotein VI precursor
IPI00102543	77.7	×	×	SLIT and NTRK-like protein 1 precursor
IPI00102821	20.7	×	×	PACAP protein
IPI00103065	29.3	×	×	MIT domain-containing protein 1
IPI00103067	27	×	×	27 kDa protein
IPI00103175	44.8	×	×	Isoform 1 of Soluble calcium-activated nucleotidase 1
IPI00103636	8.1	×	×	Isoform 2 of WAP four-disulfide core domain protein 2 precursor
IPI00103871	107.5	×	×	Isoform 1 of Roundabout homolog 4 precursor
IPI00104341	62.6	×	×	Epoxide hydrolase 2
IPI00105407	36	×	×	Aldo-keto reductase family 1 member B10
IPI00106687	25.8	×	×	Latexin
IPI00106808	29.1	×	×	LR8-like protein
IPI00107555	15.1	×	×	profilin 2 isoform b
IPI00140177	51.9	×	×	Isoform 1 of Kremen protein 1 precursor
IPI00151036	42.8	×	×	RING finger protein 13
IPI00152018	36.1	×	×	RLLV422
IPI00152491	28	×	×	CD99 antigen-like 2 isoform E3'-E4'-E3-E4
IPI00154588	58.1	×	×	Signal peptide peptidase-like 2A

IPI00156171	99	×	×	Isoform 1 of Ectonucleotide pyrophosphatase/phosphodiesterase 2
IPI00156984	22.1	×	×	Charged multivesicular body protein 1b
IPI00157414	50.2	×	×	Ectonucleotide pyrophosphatase/phosphodiesterase 6 precursor
IPI00157417	111.8	×	×	Isoform 4 of Seizure 6-like protein precursor
IPI00157687	81.4	×	×	Isoform Delta15 of Platelet endothelial cell adhesion molecule precursor
IPI00158145	32.8	×	×	leukocyte immunoglobulin-like receptor subfamily A member 5 isoform 1
IPI00159927	143	×	×	Neurocan core protein precursor
IPI00160384	41.1	×	×	Isoform Long of Delta-like protein precursor
IPI00160552	149.5	×	×	Isoform 1 of Tenascin-R precursor
IPI00162329	39.3	×	×	Isoform 1 of Transmembrane protein 25 precursor
IPI00163563	25.7	×	×	phosphatidylethanolamine-binding protein 4
IPI00165044	58.5	×	×	Chromosome 4 open reading frame 18
IPI00165360	33	×	×	3-mercaptopyruvate sulfurtransferase
IPI00165949	107.8	×	×	type 1 tumor necrosis factor receptor shedding aminopeptidase regulator isoform a
IPI00166039	25.6	×	×	scotin
IPI00166450	57	×	×	Transmembrane mucin MUC20S
IPI00166483	11.7	×	×	Chromosome 17 open reading frame 61
IPI00166766	44.6	×	×	MGC45438 protein
IPI00166768	37	×	×	TUBA6 protein
IPI00168112	23.3	×	×	Parkinson disease 7 domain containing 1
IPI00168421	57.3	×	×	F-box protein 15
IPI00168565	76.1	×	×	cyclin M3 isoform 1
IPI00168812	112.3	×	×	PTK7 protein tyrosine kinase 7 isoform d precursor
IPI00168847	45.1	×	×	Isoform 2 of Hyaluronidase-1 precursor
IPI00168884	39	×	×	Renin receptor precursor

IPI00169259	8.4	×	×	Small VCP/p97-interacting protein
IPI00169285	65.5	×	×	Hypothetical protein LOC196463
IPI00171199	27.6	×	×	proteasome alpha 3 subunit isoform 2
IPI00171391	53.4	×	×	aldehyde dehydrogenase 8A1 isoform 1
IPI00171411	46.3	×	×	Golgi phosphoprotein 2
IPI00171438	47.6	×	×	Thioredoxin domain-containing protein 5 precursor
IPI00171527	24.1	×	×	Protein C14orf32
IPI00171903	77.5	×	×	heterogeneous nuclear ribonucleoprotein M isoform a
IPI00172421	18.1	×	×	S-phase kinase-associated protein 1A isoform a
IPI00175092	43.2	×	×	E3 ubiquitin-protein ligase RNF149 precursor
IPI00176193	193.5	×	×	Isoform 1 of Collagen alpha-1(XIV) chain precursor
IPI00176221	38.7	×	×	Neuronal growth regulator 1 precursor
IPI00176427	42.8	×	×	TSLC1-like 2
IPI00177543	108.5	×	×	Isoform 1 of Peptidyl-glycine alpha-amidating monooxygenase precursor
IPI00177880	86.3	×	×	CDNA FLJ34089 fis, clone FCBBF3006249, moderately similar to Mus musculus prominin-like protein mRNA
IPI00179330	18	×	×	ubiquitin and ribosomal protein S27a precursor
IPI00179529	35.1	×	×	Isoform 1 of Sodium/potassium-transporting ATPase subunit beta-1
IPI00179589	12.8	×	×	Myotrophin
IPI00180240	5.1	×	×	thymosin-like 3
IPI00180687	53.6	×	×	Isoform 3 of Mucin and cadherin-like protein precursor
IPI00181126	9.5	×	×	similar to Nonhistone chromosomal protein HMG-17 (High-mobility group nucleosome-binding domain-containing protein 2) isoform 3
IPI00181753	44.1	×	×	CDNA FLJ13286 fis, clone OVARC1001154, highly similar to Homo sapiens clone 24720 epithelin 1 and 2 mRNA
IPI00182138	47.2	×	×	Isoform 2 of Granulins precursor
IPI00182728	49.3	×	×	Vacuolar protein sorting-associating protein 4B

IPI00183280	85.2	×	×	egf-like module containing, mucin-like, hormone receptor-like sequence 2 isoform b
IPI00183445	162.7	×	×	Isoform 1 of Latrophilin-1 precursor
IPI00183487	107.6	×	×	Xylosyltransferase 1
IPI00183703	43.1	×	×	Isoform 2 of Junctional adhesion molecule-like precursor
IPI00184363	23.7	×	×	Glycolipid transfer protein
IPI00185662	24.3	×	×	Hypothetical protein DKFZp761H2024
IPI00186290	95.2	×	×	Elongation factor 2
IPI00215612	89	×	×	Transferrin receptor 2
IPI00215746	14.6	×	×	Fatty acid-binding protein, adipocyte
IPI00215767	43.9	×	×	Isoform Long of Beta-1,4-galactosyltransferase 1
IPI00215881	20.6	×	×	Gamma crystallin D
IPI00215914	20.6	×	×	ADP-ribosylation factor 1
IPI00215918	20.4	×	×	ADP-ribosylation factor 4
IPI00215919	20.4	×	×	ADP-ribosylation factor 5
IPI00215920	20	×	×	ADP-ribosylation factor 6
IPI00215926	33.1	×	×	Asialoglycoprotein receptor 1
IPI00215997	25.3	×	×	CD9 antigen
IPI00215998	25.5	×	×	CD63 antigen
IPI00216008	63.8	×	×	Isoform Long of Glucose-6-phosphate 1-dehydrogenase
IPI00216088	15.6	×	×	Cellular retinoic acid-binding protein 2
IPI00216106	31.4	×	×	Isoform 3 of Putative GTP-binding protein 9
IPI00216136	32.5	×	×	Isoform C of Ketohexokinase
IPI00216304	10.1	×	×	Isoform Alpha of Stromal cell-derived factor 1 precursor
IPI00216308	30.6	×	×	Voltage-dependent anion-selective channel protein 1
IPI00216345	78.8	×	×	Uncharacterized protein C20orf75 precursor
IPI00216438	113.1	×	×	Solute carrier family 12 member 3
IPI00216457	14	×	×	Histone H2A type 2-A
IPI00216550	48.7	×	×	Isoform 1 of Complement decay-accelerating factor

IPI00216592	32.3	×	×	Isoform C1 of Heterogeneous nuclear ribonucleoproteins C1/C2
IPI00216780	126.9	×	×	Similar to Cartilage intermediate layer protein
IPI00216914	21.5	×	×	Vitelline membrane outer layer protein 1 homolog precursor
IPI00216984	16.8	×	×	Calmodulin-like protein 3
IPI00217253	9.6	×	×	GTP cyclohydrolase 1 feedback regulatory protein
IPI00217458	54.5	×	×	Alanine aminotransferase 1
IPI00217468	22.4	×	×	Histone H1.5
IPI00217512	77.7	×	×	Isoform 2 of Probable G-protein coupled receptor 115
IPI00217766	54.2	×	×	Lysosome membrane protein 2
IPI00217775	26.4	×	×	Isoform Short of HLA class II histocompatibility antigen gamma chain
IPI00218297	44.8	×	×	4-hydroxyphenylpyruvate dioxygenase
IPI00218343	49.9	×	×	Tubulin alpha-6 chain
IPI00218477	15.1	×	×	Fatty acid-binding protein, intestinal
IPI00218493	24.4	×	×	Hypoxanthine-guanine phosphoribosyltransferase
IPI00218568	11.9	×	×	Pterin-4-alpha-carbinolamine dehydratase
IPI00218570	28.6	×	×	Phosphoglycerate mutase 2
IPI00218693	19.5	×	×	Adenine phosphoribosyltransferase
IPI00218733	16.1	×	×	16 kDa protein
IPI00218782	33.8	×	×	Capping protein (Actin filament) muscle Z-line, beta
IPI00218834	29.1	×	×	Low affinity immunoglobulin gamma Fc region receptor III-A precursor
IPI00218851	14	×	×	Ly-6 neurotoxin-like protein 1 isoform a
IPI00218874	33.8	×	×	Isoform B of Osteopontin precursor
IPI00218875	32.4	×	×	Isoform C of Osteopontin precursor
IPI00219025	11.6	×	×	Glutaredoxin-1
IPI00219029	46.1	×	×	Aspartate aminotransferase, cytoplasmic
IPI00219067	25.6	×	×	Glutathione S-transferase Mu 2
IPI00219129	25.8	×	×	Ribosyldihydronicotinamide dehydrogenase

IPI00219156	12.7	×	×	60S ribosomal protein L30
IPI00219219	14.6	×	×	Galectin-1
IPI00219301	31.4	×	×	Myristoylated alanine-rich C-kinase substrate
IPI00219365	67.7	×	×	Moesin
IPI00219425	40.1	×	×	Isoform Beta of Poliovirus receptor precursor
IPI00219426	39.3	×	×	Isoform Gamma of Poliovirus receptor precursor
IPI00219465	47.4	×	×	Transcobalamin-2 precursor
IPI00219622	25.8	×	×	Proteasome subunit alpha type 2
IPI00219703	11.9	×	×	Parvalbumin alpha
IPI00219782	15.8	×	×	Retinol-binding protein III, cellular
IPI00219910	22.6	×	×	23 kDa protein
IPI00219953	25.9	×	×	cytidylate kinase
IPI00220143	209.7	×	×	Maltase-glucoamylase, intestinal
IPI00220219	102.4	×	×	Coatomer subunit beta'
IPI00220342	31	×	×	NG,NG-dimethylarginine dimethylaminohydrolase 1
IPI00220362	10.8	×	×	10 kDa heat shock protein, mitochondrial
IPI00220503	44.8	×	×	dynactin 2
IPI00220525	21.1	×	×	interleukin 18 binding protein precursor
IPI00220578	40.4	×	×	Guanine nucleotide-binding protein G
IPI00220613	97.4	×	×	Isoform 2C2A of Collagen alpha-2(VI) chain precursor
IPI00220739	21.5	×	×	Membrane-associated progesterone receptor component 1
IPI00220791	72	×	×	Isoform 2 of Amphiphysin
IPI00220827	4.9	×	×	Thymosin beta-10
IPI00220993	45.1	×	×	Isoform CNPI of 2',3'-cyclic-nucleotide 3'-phosphodiesterase
IPI00221088	22.5	×	×	40S ribosomal protein S9
IPI00221225	25.4	×	×	Annexin IV variant (Fragment)
IPI00221232	7.9	×	×	Guanine nucleotide-binding protein G(I)/G(S)/G(O) gamma-12 subunit precursor
IPI00221234	58.5	×	×	Similar to Aldehyde dehydrogenase family 7 member A1

IPI00221255	203.1	×	×	Isoform 2 of Myosin light chain kinase, smooth muscle
IPI00221384	205.6	×	×	Isoform Short of Collagen alpha-1(XII) chain precursor
IPI00232571	62.4	×	×	Glypican-4 precursor
IPI00240345	51.6	×	×	C-type lectin domain family 14 member A precursor
IPI00243602	32.6	×	×	C-type lectin domain family 4 member G
IPI00244512	31.4	×	×	Transmembrane protein 46 precursor
IPI00247616	81.6	×	×	Scavenger receptor with C-type lectin type I
IPI00248596	88	×	×	similar to slit homolog 1
IPI00000005	21.2	×	×	GTPase NRas precursor
IPI00000006	21.3	×	×	GTPase HRas precursor
IPI00000024	111.3	×	×	Isoform 1 of Protocadherin-1 precursor
IPI00000041	22.1	×	×	Rho-related GTP-binding protein RhoB precursor
IPI00000044	27.3	×	×	Platelet-derived growth factor B chain precursor
IPI00000073	133.9	×	×	Pro-epidermal growth factor precursor
IPI00000130	12.7	×	×	Somatostatin precursor
IPI00000160	29.4	×	×	Corticotropin-lipotropin precursor
IPI00000201	82.9	×	×	Isoform 1 of Low-density lipoprotein receptor-related protein 3 precursor
IPI00000265	97.9	×	×	hypothetical protein LOC221061
IPI00000695	41.6	×	×	Guanine nucleotide-binding protein alpha-14 subunit
IPI00000792	35.2	×	×	Quinone oxidoreductase
IPI00000811	25.4	×	×	Proteasome subunit beta type 6 precursor
IPI00000828	30.8	×	×	Proenkephalin A precursor
IPI00000849	8.2	×	×	Small proline-rich protein 2G
IPI00000853	14.7	×	×	Follitropin subunit beta precursor
IPI00000860	43.2	×	×	Fibromodulin precursor
IPI00000885	58.3	×	×	Tyrosine-protein kinase FRK
IPI00000914	15.5	×	×	Isoform 1 of Calcitonin precursor
IPI00000949	33.8	×	×	Mu-crystallin homolog
IPI00001469	9.9	×	×	Lipophilin-B precursor

IPI00001477	101.1	×	×	Isoform 1 of Epithelial discoidin domain-containing receptor 1 precursor
IPI00001592	62.6	×	×	Isoform 2 of Transmembrane glycoprotein NMB precursor
IPI00001593	55.8	×	×	Lysosomal Pro-X carboxypeptidase precursor
IPI00001611	20.1	×	×	Isoform 1 of Insulin-like growth factor II precursor
IPI00001633	74	×	×	Leucine-rich repeat transmembrane protein FLRT2 precursor
IPI00001639	97.2	×	×	Importin beta-1 subunit
IPI00001662	38	×	×	Opioid-binding protein/cell adhesion molecule precursor
IPI00001675	21.6	×	×	Isoform 1 of C-type lectin domain family 5 member
IPI00001734	40.4	×	×	Isoform 1 of Phosphoserine aminotransferase
IPI00001754	32.6	×	×	Junctional adhesion molecule A precursor
IPI00001759	31	×	×	Oxidized low-density lipoprotein receptor 1
IPI00001767	138.3	×	×	receptor-type protein tyrosine phosphatase O isoform a precursor
IPI00001793	36.4	×	×	Beta-1,3-N-acetylglucosaminyltransferase radical fringe
IPI00001872	101.1	×	×	Isoform 1 of Protocadherin gamma C3 precursor
IPI00001952	55	×	×	Endonuclease domain-containing 1 protein precursor
IPI00002147	42.6	×	×	Chitinase-3-like protein 1 precursor
IPI00002212	49.3	×	×	Isoform B of Serine/threonine-protein kinase 24
IPI00002280	27.4	×	×	ProSAAS precursor
IPI00002294	11.7	×	×	Potassium voltage-gated channel subfamily E member 3
IPI00002320	73	×	×	Leucine-rich repeat transmembrane protein FLRT3 precursor
IPI00002406	67.4	×	×	Lutheran blood group glycoprotein precursor
IPI00002412	34.2	×	×	Palmitoyl-protein thioesterase 1 precursor
IPI00002435	29.2	×	×	Tumor necrosis factor receptor superfamily member 7 precursor
IPI00002441	32.5	×	×	Syndecan-1 precursor
IPI00002460	52.7	×	×	Isoform 1 of Annexin A7
IPI00002519	53.1	×	×	Isoform 1 of Serine hydroxymethyltransferase, cytosolic
IPI00002543	45.7	×	×	Procollagen C-endopeptidase enhancer 2 precursor

IPI00002570	12.9	×	×	Eukaryotic translation initiation factor 4E-binding protein 2
IPI00002732	37.6	×	×	EXTL2 protein (Fragment)
IPI00002816	53.4	×	×	Cathepsin F precursor
IPI00002818	27.5	×	×	Isoform 1 of Kallikrein-11 precursor
IPI00002851	16.1	×	×	Cystatin D precursor
IPI00002884	35.6	×	×	CDNA: FLJ22222 fis, clone HRC01658
IPI00002910	37.9	×	×	CDNA: FLJ22573 fis, clone HSI02387
IPI00002926	31.3	×	×	CDNA FLJ12750 fis, clone NT2RP2001168, weakly similar to VERPROLIN
IPI00002966	94.3	×	×	Heat shock 70 kDa protein 4
IPI00002968	9.8	×	×	Molybdenum cofactor synthesis protein 2 small subunit
IPI00003101	30.8	×	×	Interleukin-2 receptor alpha chain precursor
IPI00003102	40.6	×	×	Ciliary neurotrophic factor receptor alpha precursor
IPI00003111	11.9	×	×	Ig kappa chain V-I region AU
IPI00003327	20.5	×	×	ADP-ribosylation factor-like protein 3
IPI00003348	37.2	×	×	Guanine nucleotide-binding protein G(I)/G(S)/G(T) subunit beta 2
IPI00003482	36.1	×	×	2,4-dienoyl-CoA reductase, mitochondrial precursor
IPI00003527	38.7	×	×	Ezrin-radixin-moesin-binding phosphoprotein 50
IPI00003648	57.2	×	×	Isoform Delta of Poliovirus receptor-related protein 1 precursor
IPI00003765	92.7	×	×	Calpain-7
IPI00003802	131.1	×	×	Alpha-mannosidase 2
IPI00003813	48.5	×	×	Nectin-like protein 2
IPI00003815	23.1	×	×	Rho GDP-dissociation inhibitor 1
IPI00003817	22.9	×	×	Rho GDP-dissociation inhibitor 2
IPI00003856	26.3	×	×	Vacuolar ATP synthase subunit
IPI00003949	17.1	×	×	Ubiquitin-conjugating enzyme E2 N
IPI00004409	96.8	×	×	Discoidin domain-containing receptor 2 precursor

IPI00004416	25.1	×	×	Charged multivesicular body protein 2a
IPI00004440	105.8	×	×	Receptor-type tyrosine-protein phosphatase-like N
IPI00004480	52.8	×	×	ADAM DEC1 precursor
IPI00004901	17.8	×	×	CDNA FLJ20242 fis, clone COLF6369
IPI00004962	81.9	×	×	golgi phosphoprotein 4
IPI00005038	14.5	×	×	Ribonuclease UK114
IPI00005040	46.6	×	×	Medium-chain specific acyl-CoA dehydrogenase, mitochondrial precursor
IPI00005118	98.9	×	×	Hexokinase-3
IPI00005142	91.9	×	×	Isoform 1 of Basic fibroblast growth factor receptor 1 precursor
IPI00005159	44.8	×	×	Actin-like protein 2
IPI00005162	20.4	×	×	Actin-related protein 2/3 complex subunit 3
IPI00005181	35	×	×	Phospholipid scramblase 1
IPI00005222	110.7	×	×	EPHB6 protein
IPI00005517	26.3	×	×	Ephrin-A5 precursor
IPI00005563	52.4	×	×	Isoform 1 of Tubulointerstitial nephritis antigen-like precursor
IPI00005564	27.6	×	×	Stanniocalcin-1 precursor
IPI00005573	23.4	×	×	Isoform 1 of 5'(3')-deoxyribonucleotidase, cytosolic type
IPI00005732	56.8	×	×	Isoform 1 of Activin receptor type 1B precursor
IPI00005733	56	×	×	TGF-beta receptor type-1 precursor
IPI00005800	48	×	×	48 kDa protein
IPI00005908	105.4	×	×	ADAMTS-1 precursor
IPI00006003	23	×	×	CD83 antigen precursor
IPI00006130	55	×	×	Uncharacterized calcium-binding protein KIAA0494
IPI00006273	37.2	×	×	CYR61 protein
IPI00006443	35.3	×	×	Lambda-crystallin homolog
IPI00006444	73.7	×	×	Isoform 1 of Sodium/potassium/calcium exchanger 2

IPI00006482	112.9	×	×	Isoform Long of Sodium/potassium-transporting ATPase alpha-1 chain precursor
IPI00006547	68.1	×	×	T-cell immunomodulatory protein precursor
IPI00006560	44.3	×	×	Isoform 1 of Serpin B13
IPI00006592	26.1	×	×	Peptide methionine sulfoxide reductase
IPI00006601	78.2	×	×	Secretogranin-1 precursor
IPI00006608	86.9	×	×	Isoform APP770 of Amyloid beta A4 protein precursor (Fragment)
IPI00006705	10	×	×	Uteroglobin precursor
IPI00006935	16.7	×	×	Eukaryotic translation initiation factor 5A-2
IPI00006967	132.3	×	×	Protocadherin-9 precursor
IPI00006971	80.9	×	×	Isoform 1 of Endosialin precursor
IPI00006988	11.4	×	×	Resistin precursor
IPI00007102	55	×	×	CGI-150 protein
IPI00007244	83.9	×	×	Isoform H17 of Myeloperoxidase precursor
IPI00007249	51.6	×	×	ectonucleotide pyrophosphatase/phosphodiesterase 4
IPI00007321	24.7	×	×	Isoform 1 of Acyl-protein thioesterase 1
IPI00007682	68.3	×	×	Vacuolar ATP synthase catalytic subunit A, ubiquitous isoform
IPI00007755	24.2	×	×	Ras-related protein Rab-21
IPI00007797	15	×	×	Fatty acid-binding protein, epidermal
IPI00007800	57.1	×	×	Angiopoietin-related protein 2 precursor
IPI00007853	29.1	×	×	Gamma-interferon-inducible lysosomal thiol reductase precursor
IPI00007926	19.1	×	×	c-Myc-responsive protein Rcl
IPI00008178	7.3	×	×	Isoform 1 of Sodium/potassium-transporting ATPase gamma chain
IPI00008239	44.8	×	×	G-protein coupled receptor family C group 5 member B precursor
IPI00008301	11	×	×	Defensin 6 precursor

IPI00008405	66	×	×	Arylsulfatase F precursor
IPI00008485	98.4	×	×	Iron-responsive element-binding protein 1
IPI00008554	16.6	×	×	Angiogenin precursor
IPI00008580	14.3	×	×	Antileukoproteinase 1 precursor
IPI00008586	60.1	×	×	Isoform 1 of Chondroitin sulfate proteoglycan 5 precursor
IPI00008727	14.7	×	×	ATP synthase lipid-binding protein, mitochondrial
IPI00008790	77.1	×	×	galactosylceramidase isoform a precursor
IPI00008820	53.2	×	×	Rhesus-associated C glycoprotein
IPI00008822	27.3	×	×	Isoform PMX1-B of Paired mesoderm homeobox protein 1
IPI00008922	14.5	×	×	Interferon-induced transmembrane protein 2
IPI00008964	22.2	×	×	Ras-related protein Rab-1B
IPI00009268	45.9	×	×	Aminoacylase-1
IPI00009328	46.7	×	×	Probable ATP-dependent RNA helicase DDX48
IPI00009607	20.7	×	×	Ras-related protein Rap-2c precursor
IPI00009619	47	×	×	Isoform 2 of Immunoglobulin superfamily member 4B
IPI00009653	42.3	×	×	Leucine-rich repeat-containing protein 19 precursor
IPI00009790	85.6	×	×	6-phosphofructokinase type C
IPI00009794	39.6	×	×	Calcium binding protein Cab45
IPI00009802	372.8	×	×	Isoform V0 of Versican core protein precursor
IPI00009851	129.2	×	×	PCDH12 protein
IPI00009852	96.4	×	×	Vacuolar proton translocating ATPase 116 kDa subunit a isoform 4
IPI00009901	14.5	×	×	Nuclear transport factor 2
IPI00009938	57.6	×	×	Isoform A of Carcinoembryonic antigen-related cell adhesion molecule 1 precursor
IPI00009997	47.1	×	×	N-acetyllactosaminide beta-1,3-N-acetylglucosaminyltransferase
IPI00010105	26.6	×	×	Eukaryotic translation initiation factor 6
IPI00010137	14.7	×	×	Isoform 2 of Leukocyte-associated immunoglobulin-like receptor 2 precursor

IPI00010191	25.2	×	×	Collectrin precursor
IPI00010277	13.9	×	×	Isoform 1 of Tumor necrosis factor receptor superfamily member 12A precursor
IPI00010314	36.3	×	×	Delta-aminolevulinic acid dehydratase
IPI00010341	25.2	×	×	Bone-marrow proteoglycan precursor
IPI00010343	100.4	×	×	Sodium/calcium exchanger 2 precursor
IPI00010348	39.6	×	×	Deoxyribonuclease-2-alpha precursor
IPI00010402	23.8	×	×	Hypothetical protein
IPI00010405	104.3	×	×	Isoform Long of Tyrosine-protein kinase transmembrane receptor ROR1 precursor
IPI00010418	118	×	×	Myosin Ic
IPI00010477	39.5	×	×	Isoform Long of Galectin-9
IPI00010676	37	×	×	Isoform 1 of Urokinase plasminogen activator surface receptor precursor
IPI00010680	94	×	×	Fibroblast growth factor receptor 2 precursor
IPI00010737	60.3	×	×	Thrombomodulin precursor
IPI00010790	41.7	×	×	Biglycan precursor
IPI00010896	26.8	×	×	Chloride intracellular channel protein 1
IPI00010949	58.3	×	×	Isoform 1 of Sialate O-acetylesterase precursor
IPI00011107	50.9	×	×	Isocitrate dehydrogenase [NADP], mitochondrial precursor
IPI00011140	39.2	×	×	Protein NOV homolog precursor
IPI00011194	24.6	×	×	Fibroblast growth factor-binding protein 2 precursor
IPI00011253	26.7	×	×	40S ribosomal protein S3
IPI00011279	28.9	×	×	Uroplakin-1a
IPI00011284	30	×	×	Isoform Membrane-bound of Catechol O-methyltransferase
IPI00011285	81.9	×	×	Calpain-1 catalytic subunit
IPI00011289	71.6	×	×	Sodium/nucleoside cotransporter 1
IPI00011564	21.6	×	×	Syndecan-4 precursor
IPI00011578	31.3	×	×	Isoform 1 of Neuroplastin precursor
IPI00011604	18.9	×	×	Glycine cleavage system H protein, mitochondrial precursor

IPI00011650	66.6	×	×	Trehalase precursor
IPI00011662	28.2	×	×	Kunitz-type protease inhibitor 2 precursor
IPI00011695	26.5	×	×	Trypsin-2 precursor
IPI00011858	12.2	×	×	PDZK1-interacting protein 1
IPI00011899	29.1	×	×	BMP and activin membrane-bound inhibitor homolog precursor
IPI00012007	47.6	×	×	Adenosylhomocysteinase
IPI00012102	62.1	×	×	N-acetylglucosamine-6-sulfatase precursor
IPI00012113	19.4	×	×	Uroplakin-2 precursor
IPI00012320	40.8	×	×	High affinity interleukin-8 receptor B
IPI00012325	55.3	×	×	matrilin 4 isoform 3 precursor
IPI00012386	59.5	×	×	Cochlin precursor
IPI00012426	50.8	×	×	Isoform 1 of Retinoid-inducible serine carboxypeptidase precursor
IPI00012512	23.4	×	×	Ras-related protein R-Ras2
IPI00012540	97.2	×	×	Prominin-1 precursor
IPI00012545	51	×	×	Isoform TGN51 of Trans-Golgi network integral membrane protein 2 precursor
IPI00012585	63.1	×	×	Beta-hexosaminidase beta chain precursor
IPI00012818	28.8	×	×	Aquaporin-2
IPI00012887	37.6	×	×	Cathepsin L precursor
IPI00012948	23.1	×	×	Heparin-binding EGF-like growth factor precursor
IPI00013004	35.1	×	×	Isoform 1 of Pyridoxal kinase
IPI00013299	23.2	×	×	neuroblastoma, suppression of tumorigenicity 1 1
IPI00013382	16.4	×	×	Cystatin SA precursor
IPI00013438	23	×	×	Immunoglobulin lambda-like polypeptide 1 precursor
IPI00013446	12.9	×	×	Prostate stem cell antigen precursor
IPI00013576	59.1	×	×	Isoform 1 of Butyrophilin subfamily 2 member A2 precursor
IPI00013682	43.9	×	×	Isoform 3 of Ecto-ADP-ribosyltransferase 3 precursor
IPI00013826	59.8	×	×	Isoform 1 of Atrial natriuretic peptide clearance receptor precursor

IPI00013895	11.7	×	×	Protein S100-A11
IPI00013955	122.1	×	×	Isoform 1 of Mucin-1 precursor
IPI00013972	38.2	×	×	Carcinoembryonic antigen-related cell adhesion molecule 8 precursor
IPI00014048	17.6	×	×	Ribonuclease pancreatic precursor
IPI00014260	259.6	×	×	deleted in malignant brain tumors 1 isoform c precursor
IPI00014363	40.4	×	×	betaine-homocysteine methyltransferase 2
IPI00014424	50.5	×	×	Elongation factor 1-alpha 2
IPI00252950	11.1	×	×	Novel protein
IPI00253036	18.8	×	×	Isoform I of CD99 antigen precursor
IPI00256974	14.7	×	×	Osteocrin precursor
IPI00259102	25.4	×	×	Mammalian ependymin-related protein 1 precursor
IPI00288947	42.1	×	×	Guanine nucleotide binding protein (G protein), q polypeptide
IPI00289204	50.7	×	×	Reticulon-4 receptor precursor
IPI00289275	132.6	×	×	Cartilage intermediate layer protein 1 precursor
IPI00289329	110.3	×	×	Ephrin type-B receptor 3 precursor
IPI00289346	51.7	×	×	Angiopoietin-related protein 6 precursor
IPI00289926	49.3	×	×	Isoform 1 of Leukocyte immunoglobulin-like receptor subfamily B member 4 precursor
IPI00289931	42.7	×	×	Isoform 1 of Mucosal addressin cell adhesion molecule 1 precursor
IPI00290089	92.1	×	×	Cadherin-17 precursor
IPI00290315	50.7	×	×	Chromogranin A precursor
IPI00290337	92	×	×	92 kDa protein
IPI00290358	15	×	×	Hypothetical protein gs103
IPI00290452	34.6	×	×	Transmembrane BAX inhibitor motif-containing protein 1
IPI00290553	98.8	×	×	10-formyltetrahydrofolate dehydrogenase
IPI00290826	20	×	×	Transmembrane protein 157
IPI00291006	35.5	×	×	Malate dehydrogenase, mitochondrial precursor

IPI00291395	74.1	×	×	fibronectin leucine rich transmembrane protein 1
IPI00291483	36.8	×	×	Aldo-keto reductase family 1 member C3
IPI00291488	13	×	×	Isoform 1 of WAP four-disulfide core domain protein 2 precursor
IPI00291928	23.8	×	×	Ras-related protein Rab-14
IPI00292130	24	×	×	Dermatopontin precursor
IPI00292532	19.6	×	×	Antibacterial protein FALL-39 precursor
IPI00292858	50	×	×	Thymidine phosphorylase precursor
IPI00292993	53.9	×	×	bactericidal/permeability-increasing protein precursor
IPI00293088	105.9	×	×	106 kDa protein
IPI00293303	49.4	×	×	Legumain precursor
IPI00293648	47.7	×	×	N-acylglucosamine 2-epimerase
IPI00293757	103.1	×	×	Isoform 1 of Netrin receptor UNC5C precursor
IPI00293853	35.6	×	×	Cell surface A33 antigen precursor
IPI00293867	12.6	×	×	D-dopachrome decarboxylase
IPI00293877	20.1	×	×	Tumor necrosis factor receptor superfamily member 17
IPI00294250	108.1	×	×	Ephrin type-A receptor 1 precursor
IPI00294615	50.2	×	×	Fibulin-5 precursor
IPI00295414	142.6	×	×	Collagen alpha-1(XV) chain precursor
IPI00295542	53.9	×	×	Nucleobindin-1 precursor
IPI00295777	37.5	×	×	Glycerol-3-phosphate dehydrogenase [NAD+], cytoplasmic
IPI00296058	49.4	×	×	EGF-containing fibulin-like extracellular matrix protein 2 precursor
IPI00296067	19.8	×	×	CD160 antigen precursor
IPI00296215	34.9	×	×	Tumor-associated calcium signal transducer 1 precursor
IPI00296351	33.2	×	×	Leucine-rich repeat-containing protein 25 precursor
IPI00296374	34.1	×	×	Isoform 1 of Zinc finger protein-like 1
IPI00296461	69.9	×	×	Isoform 1 of Sphingomyelin phosphodiesterase precursor
IPI00296713	63.5	×	×	Isoform 1 of Granulins precursor
IPI00296777	75.2	×	×	SPARC-like protein 1 precursor

IPI00296922	196.1	×	×	Laminin beta-2 chain precursor
IPI00296992	98.3	×	×	AXL receptor tyrosine kinase isoform 1
IPI00297056	53.5	×	×	Cornulin
IPI00297124	103.5	×	×	Isoform 1 of Interleukin-6 receptor subunit beta precursor
IPI00297224	88.2	×	×	sushi domain containing 5
IPI00297252	100.5	×	×	Isoform 1 of Extracellular sulfatase Sulf-2 precursor
IPI00297263	147.5	×	×	similar to HEG homolog 1
IPI00297420	60.2	×	×	Frizzled 4 precursor
IPI00297487	37.4	×	×	Cathepsin H precursor
IPI00297646	138.9	×	×	Collagen alpha-1(I) chain precursor
IPI00297910	35.7	×	×	Tumor-associated calcium signal transducer 2 precursor
IPI00298237	61.2	×	×	Isoform 1 of Tripeptidyl-peptidase 1 precursor
IPI00298388	28.2	×	×	HGFL protein
IPI00298558	24.7	×	×	Programmed cell death protein 10
IPI00298793	100.9	×	×	Beta-mannosidase precursor
IPI00299026	53.7	×	×	Alpha-L-fucosidase
IPI00299116	55.6	×	×	Podocalyxin-like protein 1 precursor
IPI00299485	68.6	×	×	Complement component C1q receptor precursor
IPI00299571	53.9	×	×	Isoform 2 of Protein disulfide-isomerase A6 precursor
IPI00299669	70.9	×	×	Mannosyl-oligosaccharide 1,2-alpha-mannosidase IC
IPI00299724	43.3	×	×	Isoform 1 of Signal-regulatory protein beta-1 precursor
IPI00299977	13.8	×	×	14 kDa phosphohistidine phosphatase
IPI00300086	30.8	×	×	Nicotinate-nucleotide pyrophosphorylase
IPI00300620	13.9	×	×	Interferon-induced transmembrane protein 1
IPI00301395	54.2	×	×	Probable serine carboxypeptidase CPVL precursor
IPI00301459	46.7	×	×	1-O-acylceramide synthase precursor
IPI00301464	42.9	×	×	Isoform SGCA-1 of Alpha-sarcoglycan precursor
IPI00301869	69.5	×	×	Isoform 2 of Mucin and cadherin-like protein precursor
IPI00302592	280	×	×	filamin 1
IPI00302614	31.3	×	×	Immune costimulatory protein B7-H4

IPI00303161	41.2	×	×	Endothelial cell-selective adhesion molecule precursor
IPI00303174	50	×	×	Homogentisate 1,2-dioxygenase
IPI00303476	56.6	×	×	ATP synthase subunit beta, mitochondrial precursor
IPI00304814	22.1	×	×	Isoform Mitochondrial of Phospholipid hydroperoxide glutathione peroxidase, mitochondrial precursor
IPI00304865	93.5	×	×	transforming growth factor, beta receptor III
IPI00304903	9.9	×	×	Cornifin B
IPI00304999	104.8	×	×	Tyrosine-protein kinase transmembrane receptor ROR2 precursor
IPI00305010	35.5	×	×	Hypothetical protein FLJ11151
IPI00305286	29	×	×	15-hydroxyprostaglandin dehydrogenase
IPI00305551	42.1	×	×	Guanine nucleotide-binding protein subunit alpha-11
IPI00305978	39.6	×	×	Aflatoxin B1 aldehyde reductase member 2
IPI00306322	167.5	×	×	Collagen alpha-2(IV) chain precursor
IPI00306339	33.8	×	×	secreted phosphoprotein 1 isoform b
IPI00307446	142.2	×	×	142 kDa protein
IPI00328113	312.3	×	×	Fibrillin-1 precursor
IPI00328243	54.7	×	×	Phospholipase D family, member 3
IPI00328431	103.6	×	×	Isoform 1 of Netrin receptor UNC5B precursor
IPI00328488	113.9	×	×	Isoform 1 of Epididymis-specific alpha-mannosidase precursor
IPI00328703	30.7	×	×	OAF homolog
IPI00328829	106.1	×	×	inter-alpha trypsin inhibitor heavy chain precursor 5 isoform 1
IPI00329538	36.8	×	×	Prostasin precursor
IPI00329573	333.2	×	×	Isoform Long of Collagen alpha-1(XII) chain precursor
IPI00329685	53.8	×	×	Hypothetical protein DKFZp686G12235
IPI00329688	38.2	×	×	Protein YIPF3
IPI00332887	55	×	×	signal-regulatory protein alpha precursor

IPI00333140	78.5	×	×	Delta and Notch-like epidermal growth factor-related receptor precursor
IPI00333234	12.5	×	×	12 kDa protein
IPI00333776	144.1	×	×	Isoform 1 of Neuronal cell adhesion molecule precursor
IPI00334238	52.8	×	×	neuronal pentraxin receptor
IPI00334775	84.8	×	×	85 kDa protein
IPI00335009	106.2	×	×	IMP dehydrogenase/GMP reductase family protein
IPI00337415	40.2	×	×	Guanine nucleotide-binding protein G(i), alpha-1 subunit
IPI00337687	39.7	×	×	Isoform 3 of Interleukin-1 receptor accessory protein precursor
IPI00337741	81.2	×	×	Acylamino-acid-releasing enzyme
IPI00374068	116.5	×	×	Isoform 1 of ADAMTS-like protein 4 precursor
IPI00374354	15.8	×	×	VPS37D protein
IPI00375676	21.3	×	×	Ferritin light polypeptide variant
IPI00375704	28.8	×	×	Hypothetical protein DKFZp686I0180 (Fragment)
IPI00376035	20.8	×	×	Hypothetical protein
IPI00376243	32.8	×	×	B- and T-lymphocyte attenuator precursor
IPI00376258	53.2	×	×	Solute carrier family 36 (Proton/amino acid symporter), member 2
IPI00376403	58.4	×	×	Isoform 1 of Kunitz-type protease inhibitor 1 precursor
IPI00376457	25.9	×	×	Immunoglobulin-like domain containing protein
IPI00382452	21.7	×	×	Isoform 1 of Charged multivesicular body protein 1a
IPI00382456	×	×	×	Ig heavy chain V-I region ND precursor (Fragment)
IPI00382470	98.2	×	×	Heat shock protein HSP 90-alpha 2
IPI00382474	13.5	×	×	Ig heavy chain V-III region TRO
IPI00382492	12.9	×	×	Ig heavy chain V-III region LAY
IPI00382821	10.6	×	×	putative nuclear protein ORF1-FL49
IPI00383046	28	×	×	carboxymethylenebutenolidase-like
IPI00383717	23.8	×	×	Intron-containing kallikrein (Fragment)
IPI00383951	242.6	×	×	Isoform 3 of Protein sidekick-1 precursor

IPI00384016	29.6	×	×	Full-length cDNA 5-PRIME end of clone CS0DJ009YL13 of T cells (Jurkat cell line) of Homo sapiens (Fragment)
IPI00384401	11.8	×	×	Myosin-reactive immunoglobulin kappa chain variable region (Fragment)
IPI00384576	14.1	×	×	Ig kappa chain V-III region HIC precursor
IPI00384643	35.5	×	×	Isoform 1 of Putative quinone oxidoreductase
IPI00384722	28.4	×	×	INM02
IPI00384746	63.1	×	×	Copine-8
IPI00384770	25.3	×	×	HGFL(S) protein
IPI00384998	139.9	×	×	Isoform 7 of Neurofascin precursor
IPI00385172	24.4	×	×	Mucin 1, transmembrane
IPI00386032	12.3	×	×	12 kDa protein
IPI00386035	115.3	×	×	Profilaggrin (Fragment)
IPI00386131	14.1	×	×	Ig kappa chain V-III region IARC/BL41 precursor
IPI00386132	14.6	×	×	Ig kappa chain V-IV region JI precursor
IPI00386354	17	×	×	CDNA FLJ14048 fis, clone HEMBA1006650, weakly similar to ARP2/3 COMPLEX 20 KD SUBUNIT
IPI00386755	54.4	×	×	ERO1-like protein alpha precursor
IPI00387096	12.1	×	×	Ig kappa chain V-I region Kue
IPI00387109	12.7	×	×	Ig kappa chain V-II region FR
IPI00387118	11.7	×	×	Ig kappa chain V-III region WOL
IPI00395783	142.6	×	×	CDNA FLJ90018 fis, clone HEMBA1000732, highly similar to Homo sapiens latent transforming growth factor-beta binding protein-4
IPI00395826	45	×	×	Serine protease hepsin
IPI00395847	9.4	×	×	LY6/PLAUR domain containing 1 isoform b
IPI00396383	46.8	×	×	von Willebrand factor A domain-related protein isoform 1
IPI00396423	107	×	×	Alcadein beta
IPI00397393	203.6	×	×	similar to K06A9.1b isoform 2
IPI00397949	77.1	×	×	G protein-coupled receptor 56 isoform b

IPI00398129	26.8	×	×	Similar to dehydrogenase/reductase (SDR family) member 6
IPI00398625	282.4	×	×	Hornerin
IPI00398918	50.8	×	×	Hypothetical protein DKFZp686I21167
IPI00399007	46.1	×	×	Hypothetical protein DKFZp686I04196 (Fragment)
IPI00401264	47	×	×	Thioredoxin domain-containing protein 4 precursor
IPI00409659	65.8	×	×	Ubiquilin-2
IPI00410122	55.8	×	×	Isoform 1 of Plexin domain-containing protein 1 precursor
IPI00410214	33.4	×	×	Isoform 1 of 3'(2'),5'-bisphosphate nucleotidase 1
IPI00410240	33.3	×	×	Isoform 2 of Retinoic acid receptor responder protein 1
IPI00410487	25	×	×	CDNA PSEC0250 fis, clone NT2RP3001874, highly similar to Twisted gastrulation
IPI00410488	57.2	×	×	Isoform 1 of CD276 antigen precursor
IPI00410585	134.3	×	×	Isoform 1 of Crumbs homolog 2 precursor
IPI00411478	84	×	×	Isoform C of Neural cell adhesion molecule 1, 120 kDa isoform precursor
IPI00412212	43	×	×	43 kDa protein
IPI00412272	12.3	×	×	SH3 domain-binding glutamic acid-rich-like protein 2
IPI00412713	52	×	×	Sorting and assembly machinery component 50 homolog
IPI00412771	71.5	×	×	CD2-associated protein
IPI00412982	272.5	×	×	Neurogenic locus notch homolog protein 1 precursor
IPI00413344	18.7	×	×	Cofilin-2
IPI00413587	22	×	×	Isoform 1 of BH3-interacting domain death agonist
IPI00413641	35.7	×	×	Aldose reductase
IPI00414231	76.2	×	×	Isoform 1 of Low-density lipoprotein receptor-related protein 10 precursor
IPI00414294	53	×	×	53 kDa protein
IPI00414542	31	×	×	Isoform 1 of Programmed cell death 1 ligand 2 precursor
IPI00414684	45.5	×	×	Isoform 2 of Semenogelin-1 precursor
IPI00414717	137.2	×	×	golgi apparatus protein 1
IPI00414784	33.2	×	×	Isoform 1 of CMRF35-H antigen precursor

IPI00414896	29.5	×	×	Isoform 1 of Ribonuclease T2 precursor
IPI00418125	43.1	×	×	Isoform 1 of Layilin precursor
IPI00418163	192.8	×	×	complement component 4B preproprotein
IPI00418418	56.8	×	×	ATPase, H+ transporting, lysosomal 56/58kDa, V1 subunit B1
IPI00418446	46.5	×	×	N-acylsphingosine amidohydrolase (acid ceramidase) 1 isoform b
IPI00418594	66.1	×	×	CDNA FLJ16275 fis, clone NT2RI2027157, moderately similar to Mouse SDR2 mRNA
IPI00419215	161.3	×	×	CDNA FLJ41598 fis, clone CTONG2025496, weakly similar to ALPHA-2- MACROGLOBULIN
IPI00419327	536.6	×	×	FAT tumor suppressor homolog 4
IPI00419442	16	×	×	IGLV6-57 protein
IPI00419668	59.1	×	×	Ectonucleotide pyrophosphatase/phosphodiesterase 7
IPI00419720	42.2	×	×	KFQG729
IPI00419724	92.8	×	×	semaphorin 4B precursor
IPI00419916	57.3	×	×	Alkaline phosphatase, tissue-nonspecific isozyme precursor
IPI00419966	53.9	×	×	Isoform 2 of Target of Nesh-SH3 precursor
IPI00426051	51.1	×	×	Hypothetical protein DKFZp686C15213
IPI00428511	46.6	×	×	Neurexin-1-beta precursor
IPI00428691	115.5	×	×	Isoform 2 of Desmoglein-4 precursor
IPI00430842	52.9	×	×	IGHA1 protein
IPI00431197	37.1	×	×	Soluble form of receptor for advanced glycation endproducts precursor
IPI00432723	96.7	×	×	Isoform 1 of Xylosyltransferase 2
IPI00439344	75.6	×	×	Xaa-Pro aminopeptidase 2 precursor
IPI00440160	39.8	×	×	UEVLD protein
IPI00440493	59.8	×	×	ATP synthase subunit alpha, mitochondrial precursor
IPI00440577	26.2	×	×	Hypothetical protein LOC651928
IPI00440764	50.5	×	×	GGT6 protein
IPI00440822	118.6	×	×	Isoform 1 of Target of Nesh-SH3 precursor

IPI00440932	90.6	×	×	Isoform 1 of ADAM 9 precursor
IPI00441344	76.1	×	×	Beta-galactosidase precursor
IPI00441498	29.8	×	×	Folate receptor alpha precursor
IPI00442865	37.8	×	×	CDNA FLJ26488 fis, clone KDN05770, highly similar to Bumetanide- sensitive sodium-(potassium)-chloride cotransporter 2
IPI00443799	119.8	×	×	hypothetical protein LOC124565 isoform a
IPI00443898	121.3	×	×	Solute carrier family 12 member 1
IPI00444383	89.7	×	×	Isoform 1 of Interleukin-4 receptor alpha chain precursor
IPI00445227	57.6	×	×	Isoform 2 of Cell surface glycoprotein MUC18 precursor
IPI00445716	44.5	×	×	Isoform 1 of GDNF family receptor alpha-3 precursor
IPI00445971	61.2	×	×	CDNA FLJ43251 fis, clone HEART2006131, weakly similar to Mus musculus 2-hydroxyphytanoyl-CoA lyase
IPI00447485	22.6	×	×	WISP2 protein
IPI00448095	25.9	×	×	L-xylulose reductase
IPI00450309	24.9	×	×	IGLC2 protein
IPI00451401	27	×	×	Isoform 2 of Triosephosphate isomerase
IPI00452565	27.5	×	×	Isoform 1 of Endomucin precursor
IPI00455739	41.8	×	×	Isoform 1 of Beta-1,3-N-acetylglucosaminyltransferase lunatic fringe
IPI00456030	14	×	×	Novel protein
IPI00456145	71.1	×	×	Sodium-dependent neutral amino acid transporter B
IPI00456238	15.6	×	×	hemoglobin mu chain
IPI00456623	99.1	×	×	Isoform 1 of Brevican core protein precursor
IPI00456942	34.3	×	×	poliovirus receptor related immunoglobulin domain containing
IPI00465070	15.3	×	×	Histone H3.1
IPI00465085	60.3	×	×	Nicotinate phosphoribosyltransferase-like protein
IPI00465121	41.5	×	×	Galphai2 protein
IPI00465184	51	×	×	Guanine deaminase

IPI00465187	92.5	×	×	Isoform 1 of Angiotensin-converting enzyme 2 precursor
IPI00465255	17.2	×	×	Uterine-specific proline-rich acidic protein
IPI00465315	11.6	×	×	Cytochrome c
IPI00465325	69.9	×	×	leucine-rich repeat neuronal 6A
IPI00465431	26.1	×	×	Galectin-3
IPI00470360	83.5	×	×	Isoform 1 of Kin of IRRE-like protein 1 precursor
IPI00470652	26.1	×	×	Single-chain Fv (Fragment)
IPI00472082	42.3	×	×	42 kDa protein
IPI00472151	40.7	×	×	HLA class I histocompatibility antigen, A-23 alpha chain precursor
IPI00472961	25.9	×	×	IGKC protein
IPI00473014	18.4	×	×	Destrin
IPI00473104	25.3	×	×	NKG2-D type II integral membrane protein
IPI00477432	36.7	×	×	37 kDa protein
IPI00478600	25.9	×	×	Hypothetical protein
IPI00479018	34.8	×	×	Syntenin iSoform 3
IPI00479708	68.6	×	×	IGHM protein
IPI00479877	56.3	×	×	aldehyde dehydrogenase 9A1
IPI00479928	11.4	×	×	11 kDa protein
IPI00513767	22.9	×	×	Prostaglandin D2 synthase 21kDa
IPI00514755	28.1	×	×	Calcium binding protein Cab45
IPI00514824	32.4	×	×	Complement component C4B (Fragment)
IPI00514908	64.1	×	×	Novel protein
IPI00549291	66.2	×	×	IGHM protein
IPI00549467	30.6	×	×	Nitrilase family, member 2
IPI00549521	80.2	×	×	Isoform 1 of Choline transporter-like protein 2
IPI00549725	28.7	×	×	Phosphoglycerate mutase 1
IPI00550069	49.8	×	×	Ribonuclease inhibitor
IPI00550181	23.9	×	×	Charged multivesicular body protein 2b
IPI00550363	22.3	×	×	Transgelin-2

IPI00550488	35.3	×	×	TALDO1 protein
IPI00551024	59	×	×	Dihydroxyacetone kinase
IPI00552528	37	×	×	OTTHUMP00000029984
IPI00552937	38.3	×	×	hypothetical protein LOC387921 isoform a
IPI00553215	10.4	×	×	V1-5 protein (Fragment)
IPI00553238	38.5	×	×	CDNA FLJ42617 fis, clone BRACE3014807
IPI00554521	21.1	×	×	Ferritin heavy chain
IPI00554811	19.5	×	×	Actin-related protein 2/3 complex subunit 4
IPI00555628	89.2	×	×	Neural cell adhesion molecule 1, 120 kDa isoform variant (Fragment)
IPI00555734	32.1	×	×	asparaginase-like 1 protein
IPI00555945	24.8	×	×	IGLC2 protein
IPI00555956	29.2	×	×	Proteasome subunit beta type 4 precursor
IPI00556431	16.1	×	×	Chitinase 3-like 1 variant (Fragment)
IPI00604590	32.6	×	×	NME1-NME2 protein
IPI00607799	26.7	×	×	Isoform 1 of 3-hydroxybutyrate dehydrogenase type 2
IPI00640083	223.7	×	×	Complement component (3b/4b) receptor 1, including Knops blood group system
IPI00641251	29	×	×	CD320 antigen precursor
IPI00641693	400.3	×	×	400 kDa protein
IPI00642632	11.4	×	×	Protein
IPI00643202	48.4	×	×	SERPINB12 protein
IPI00643994	77.3	×	×	CDNA FLJ46245 fis, clone TESTI4020596, highly similar to Homo sapiens calpain 5
IPI00644034	20.6	×	×	21 kDa protein
IPI00644131	113.6	×	×	Truncated lysosomal acid alpha-mannosidase
IPI00645363	51.7	×	×	Hypothetical protein DKFZp686P15220
IPI00646217	37.4	×	×	CDNA FLJ42063 fis, clone SYNOV2005817, highly similar to CYTOKINE RECEPTOR CLASS-II CRF2-4
IPI00646291	49.4	×	×	Integral membrane protein GPR180 precursor

IPI00646304	23.7	×	×	peptidylprolyl isomerase B precursor
IPI00646689	13.9	×	×	Thioredoxin-like protein 5
IPI00647357	30.5	×	×	OSCAR protein
IPI00651768	33.4	×	×	Isoform 1 of Hepatitis A virus cellular receptor 2 precursor
IPI00654656	124.1	×	×	Serine protease inhibitor Kazal-type 5 long isoform
IPI00655531	79.2	×	×	Choline transporter-like protein 4
IPI00719233	53.2	×	×	IGHA1 protein
IPI00719373	25.1	×	×	IGLC1 protein
IPI00719622	7.8	×	×	40S ribosomal protein S28
IPI00741107	203.1	×	×	similar to melanoma inhibitory activity 3 isoform 3
IPI00744702	28.8	×	×	Isoform 1 of Protein FAM125A
IPI00745233	25.5	×	×	Glutathione S-transferase A2
IPI00746777	41.6	×	×	Class III alCohol dehydrogenase 5 Chi subunit
IPI00748158	65	×	×	IGHM protein
IPI00784430	12.6	×	×	Ig kappa chain V-III region VG precursor (Fragment)
IPI00784519	25	×	×	Hypothetical protein
IPI00784589	25	×	×	Hypothetical protein DKFZp781M0386
IPI00784661	25.8	×	×	Hypothetical protein
IPI00784669	25.8	×	×	Hypothetical protein
IPI00784713	24.9	×	×	Hypothetical protein
IPI00784758	52.2	×	×	Hypothetical protein DKFZp686M08189
IPI00784828	52.1	×	×	Hypothetical protein DKFZp686C11235
IPI00784842	52	×	×	Hypothetical protein DKFZp686G11190
IPI00784865	25.8	×	×	Hypothetical protein
IPI00784985	25.5	×	×	Hypothetical protein
IPI00785050	24.9	×	×	Hypothetical protein
IPI00785196	24.6	×	×	Hypothetical protein
IPI00000190	25.8	×	×	CD81 antigen
IPI00003799	20.9	×	×	Isoform 2 of Heme-binding protein 2

IPI00004397	23.4	×	×	Ras-related protein Ral-B
IPI00005719	22.7	×	×	RAB1A, member RAS oncogene family
IPI00006212	53.9	×	×	Transmembrane protease, serine 2 precursor
IPI00007067	17.1	×	×	Golgi-associated plant pathogenesis-related protein 1
IPI00010182	10	×	×	Endozepine
IPI00010491	24.5	×	×	Ras-related protein Rab-27B
IPI00012011	18.4	×	×	Cofilin-1
IPI00016381	24.9	×	×	Isoform Long of Ras-related protein Rab-27A
IPI00018146	27.8	×	×	14-3-3 protein theta
IPI00021263	27.7	×	×	14-3-3 protein zeta/delta
IPI00022213	42.4	×	×	Gastricsin precursor
IPI00023504	25	×	×	Ras-related protein Rab-3A
IPI00024915	22	×	×	Isoform Mitochondrial of Peroxiredoxin-5, mitochondrial precursor
IPI00025512	22.8	×	×	Heat-shock protein beta-1
IPI00027341	38.5	×	×	Macrophage capping protein
IPI00027500	21.8	×	×	Transforming protein RhoA precursor
IPI00028481	23.7	×	×	Ras-related protein Rab-8A
IPI00031169	23.5	×	×	Ras-related protein Rab-2A
IPI00075248	16.7	×	×	Calmodulin
IPI00100154	30.3	×	×	Toll-interacting protein
IPI00148063	21.1	×	×	Heme-binding protein 1
IPI00156689	41.9	×	×	Synaptic vesicle membrane protein VAT-1 homolog
IPI00216057	38.2	×	×	Sorbitol dehydrogenase
IPI00216318	28.1	×	×	tyrosine 3-monooxygenase/tryptophan 5-monooxygenase activation protein, beta polypeptide
IPI00219446	20.9	×	×	Phosphatidylethanolamine-binding protein 1
IPI00219525	53	×	×	6-phosphogluconate dehydrogenase, decarboxylating
IPI00220271	36.4	×	×	Alcohol dehydrogenase
IPI00220301	24.9	×	×	Peroxiredoxin-6

IPI00220642	28.2	×	×	14-3-3 protein gamma
IPI00220766	20.6	×	×	Lactoylglutathione lyase
IPI00291005	36.3	×	×	Malate dehydrogenase, cytoplasmic
IPI00299024	22.6	×	×	Brain acid soluble protein 1
IPI00300562	24.8	×	×	Ras-related protein Rab-3B
IPI00413451	46.4	×	×	Hypothetical protein DKFZp686I04222
IPI00414320	54.4	×	×	Annexin A11
IPI00455315	38.5	×	×	Annexin A2
IPI00645078	117.8	×	×	Ubiquitin-activating enzyme E1

abbreviations: TheoMW(theoretical Molecular weight), ExpMW_pla(experimental molecular weight in plasma), ExpMW_uri(exprimental molecular weight in urine),f(fragments), ptm(post-translational modification)
